# World reclassification of the Cardiophorinae (Coleoptera, Elateridae), based on phylogenetic analyses of morphological characters

**DOI:** 10.3897/zookeys.655.11894

**Published:** 2017-02-13

**Authors:** Hume B. Douglas

**Affiliations:** 1 Canadian National Collection of Insects, Arachnids and Nematodes, Agriculture and Agri-Food Canada, KW Neatby Building, 960 Carling Ave, Ottawa, ON K1A 0C6, CANADA

**Keywords:** Cardiophorinae, Negastriinae, Physodactylinae, Phylogeny, Biogeography

## Abstract

The prior genus-level classification of Cardiophorinae had never been assessed phylogenetically, and not revised since 1906. A phylogeny for Cardiophorinae and Negastriinae is inferred by Bayesian analyses of 163 adult morphological characters to revise the generic classification. Parsimony analysis is also performed to assess the sensitivity of the Bayesian results to the choice of optimality criterion. Bayesian hypothesis testing rejected monophyly for: Negastriinae; Cardiophorinae (but monophyletic after addition of four taxa); Cardiophorini; cardiophorine genera *Aphricus* LeConte, 1853; *Aptopus* Eschscholtz, 1829; *Cardiophorus* Eschscholtz, 1829; *Cardiotarsus* Eschscholtz, 1836; *Paracardiophorus* Schwarz, 1895; *Phorocardius* Fleutiaux, 1931; *Dicronychus* *sensu* Platia, 1994; *Dicronychus* *sensu* Méquignon, 1931; *Craspedostethus* *sensu* Schwarz, 1906 (i.e., including *Tropidiplus* Fleutiaux, 1903); *Paracardiophorus* *sensu* Cobos, 1970, although well-supported alternative classifications were available for only some. Based on taxonomic interpretation of phylogenetic results: Nyctorini is **syn. n.** of Cardiophorini; *Globothorax* Fleutiaux, 1891 (Physodactylinae), *Margogastrius* Schwarz, 1903 (Physodactylinae), and *Pachyelater* Lesne, 1897 (Dendrometrinae) are transferred to Cardiophorinae. The following changes are proposed for cardiophorine genera: *Aptopus* Eschscholtz, 1829 is redefined to exclude *Horistonotus*-like species; *Coptostethus* Wollaston, 1854 is subgenus of *Cardiophorus*; *Dicronychus* Brullé, 1832 and *Diocarphus* Fleutiaux, 1947, *Metacardiophorus* Gurjeva, 1966, *Platynychus* Motschulsky, 1858, and *Zygocardiophorus* Iablokoff-Khnzorian and Mardjanian, 1981 are placed at genus rank; *Paracardiophorus* Schwarz, 1895 is redefined based on North American and Eurasian species only; *Horistonotus* Candèze, 1860 redefined to include species with multiple apices on each side of their tarsal claws; *Patriciella* Van Zwaluwenburg, 1953 is **syn. n.** of *Aphricus* LeConte, 1853; *Teslasena* Fleutiaux, 1892 (Physodactylinae) is **syn. n.** of *Globothorax* Fleutiaux, 1891. The following new genera are described: *Austrocardiophorus* (type species: *Cardiophorus humeralis* Fairmaire and Germain, 1860); *Chileaphricus* (type species: *Aphricus chilensis* Fleutiaux, 1940); *Floridelater* (type species: *Coptostethus americanus* Horn, 1871, transferred from Negastriinae to Cardiophorinae). *Paradicronychus* (*nomen nudum*), is **syn. n.** of *Cardiophorus* Eschscholtz, 1829. Generic reassignments to make *Cardiodontulus*, *Cardiophorus*, *Cardiotarsus*, *Paracardiophorus* consistent with phylogenetically revised genus concepts resulted in 84 new combinations. Lectotypes are designated for 29 type species to fix generic concepts: *Anelastes femoralis* Lucas, 1857; *Aphricus chilensis* Fleutiaux, 1940; *Athous argentatus* Abeille de Perrin, 1894; *Cardiophorus adjutor* Candèze, 1875; *Cardiophorus florentini* Fleutiaux, 1895; *Cardiophorus inflatus* Candèze, 1882; *Cardiophorus luridipes* Candèze, 1860; *Cardiophorus mirabilis* Candèze, 1860; *Cardiophorus musculus* Erichson, 1840; *Cardiotarsus capensis* Candèze, 1860; *Cardiotarsus vitalisi* Fleutiaux, 1918; *Craspedostethus rufiventris* Schwarz, 1898; *Elater cinereus* Herbst, 1784; *Elater minutissimus* Germar, 1817; *Elater sputator* Linnaeus, 1758; *Elater thoracicus* Fabricius, 1801; *Eniconyx pullatus* Horn, 1884; *Esthesopus castaneus* Eschscholtz, 1829; *Gastrimargus schneideri* Schwarz, 1902; *Globothorax chevrolati* Fleutiaux, 1891; *Horistonotus flavidus* Candèze, 1860; *Horistonotus simplex* LeConte, 1863; *Lesnelater madagascariensis* Fleutiaux, 1935; *Oedostethus femoralis* LeConte, 1853; *Phorocardius solitarius* Fleutiaux, 1931; *Platynychus indicus* Motschulsky, 1858; *Platynychus mixtus* Fleutiaux, 1931; *Triplonychus acuminatus* Candèze, 1860; *Tropidiplus tellinii* Fleutiaux, 1903. A key to genera and diagnoses are provided for all genera and subgenera. A bibliographic synonymy includes references for all taxonomic changes to genera and new species through 2015.

## Introduction

The Cardiophorinae are known from all continents except Antarctica and from most large temperate and tropical islands. While larvae of *Horistonotus uhleri* Horn, 1871 attack roots of corn, cotton, oats, peanuts and tobacco (Gibson 1916), most species are probably carnivores. This, because of observed insectivorous behaviour (Devetak and Arnett 2012), and long paddle-like mandibles, which appear better adapted for locomotion and puncturing prey than chewing plant materials. Most larval cardiophorines inhabit soil (many in sandy soil), and dead or hollow trees (Palm 1972). Larvae move by pushing soil particles aside with their mandibles and maxilla-labial complex, while the thoracic legs and hydrostatic extension and contraction of the abdomen propel the larva forward. Traction for hydrostatic motion is partly by expansion and contraction of digitate anal lobes and smaller lateral abdominal projections (video available upon request). Cardiophorines are probably sometimes trophically important: adults are among the most abundant insects attracted to lights in some desert habitats (e.g. spp. of *Horistonotus* Candèze, 1860, *Esthesopus* Eschscholtz, 1829 and *Aptopus* Eschscholtz, 1829 during the rainy season, Sonoran Desert, USA). They are also important pollinators: for example one South African orchid is pollinated primarily by a *Cardiophorus* Eschscholtz, 1829 species (Peter and Johnson 2005). Many Cardiophorinae are rare or localized to particular sand deposits or montane forests (e.g. Douglas 2003, Girard 2003, and Platia and Gudenzi 2000b) and some are probably at extinction risk. Presently only *Cardiophorus gramineus* Scopoli, 1763 has formal conservation protection (as one of twelve beetle species protected by law in Sweden (Ljungberg et al. 2010).

Prior to this study Cardiophorinae had 29 described extant genera worldwide including about 1100 extant species, and two fossil species (Cockerell 1925, Eocene: USA; Hawkswood et al. 2009, Pleistocene: Madagascar), with one genus known only from fossils (*Mionelater* Becker, Miocene: Mexico). However, it remains unknown whether the subfamily and its genera are monophyletic.

### History of genera and tribes

Eschscholtz (1829) named three cardiophorine genera (*Aptopus*, *Cardiophorus*, and *Esthesopus*) in his initial division of genus *Elater* Linnaeus, 1758. Fifteen additional Cardiophorine genera were described between 1800 and 1900, of which four to seven were in synonymy at the outset of this study according to various authors (listed in synonymy). Candèze (1860) wrote the first genus level revision of the Cardiophorinae, in his four-volume total revision of Elateridae (Candèze 1857–63). The most recent genus level revision was published by Schwarz (1906). The monophyly and membership of all genus level groups remain untested hypotheses because phylogenetic analysis has never been applied to any of the genera.

The Cardiophorinae were divided into two tribes when Gurjeva (1974a) transferred the monotypic tribe Nyctorini from the Elaterinae into the Cardiophorinae. This transfer was made without comment, suggesting Gurjeva did not realize Nyctorini was incorrectly described as Elaterinae. So, division of the Cardiophorinae into tribes Cardiophorini and Nyctorini was perhaps accidental. Dolin (1975) placed Nyctorini in synonymy under Cardiophorini, effectively eliminating tribal level structure. Stibick (1979a) removed Nyctorini from synonymy because, although he dismissed two diagnostic characters as weak, he did not know of other Cardiophorinae with absent [short] adult prosternal lobes. This study will use phylogenetic results to assess whether tribe Nyctorini should be a synonym of Cardiophorini.

### Phylogeny and monophyly of subfamily, tribes and genera

Although little-tested phylogenetically, the monophyly of the Cardiophorinae has never been questioned in the literature. Subfamily-level non-monophyly is however possible due to inconsistencies in the characters used to separate Cardiophorinae from Negastriinae. Several apparent synapomorphies unite Negastriinae and Cardiophorinae: closed mesocoxal cavities; hind wing without anal cell; and basally-fused parameres, articulated at their midlength (Douglas 2011). The characters used to distinguish these two subfamilies since their description (Candèze 1860, Negastriinae as part of Cryptohypnites) are the short cardiophorine prosternal process, the broad prosternum of most Negastriinae, and the heart-shaped scutellum of most Cardiophorinae. However, Stibick (1979a) noticed these were not universal and has omitted the prosternal width character and qualified the other two characters with the terms “usually” and “normally.”

Other putative evidence for cardiophorine monophyly comes from the distinctive cardiophorine larvae (Hyslop 1921, Ôhira 1962, Stibick 1979a, Calder 1996). Potentially synapomorphic characteristics include: deeply cleft mandibles, thread-like abdomen with extra pseudosegmentation, and digitate anal lobes (Stibick 1979a). Although this larval type is known from Europe (Palm 1972), Central Asia (Atamuradov 1993), northeast Asia (Dolin and Gurjeva 1975), Japan (Ôhira 1962), Australia (Calder 1996), New Zealand (collections only, without digitate anal lobes), North America (Tenhet 1941) and South America (Costa et al. 1988), larvae remain unknown for most genera and species. Thus, it remains unknown whether these probable synapomorphies are of the Cardiophorinae alone, of the Cardiophorinae and other taxa, or of only some Cardiophorinae. Although these strong larval morphological characters exist and are possible evidence for cardiophorine monophyly, too few larvae are known for them to yet be used to test monophyly.

A previous study (Douglas 2011), analyzing elaterid phylogeny using adult morphology, found: the included Cardiophorinae were closest to *Margogastrius* Schwarz, 1903 and *Teslasena* Fleutiaux, 1892 (Physodactylinae) and then Negastriinae. There the included Cardiophorinae were monophyletic, excluding *Exoeolus* Broun, 1893 and three fossil genera (*Crioraphes* Iablokoff-Khnzorian, 1961; *Pseudocardiophorites* Dolin, 1976 and *Protocardiophorus* Dolin, 1976). It also showed that Cardiophorinae may render Negastriinae paraphyletic. Furthermore, *Tropihypnus* Reitter, 1905 and a paraphyletic Hypnoidini (Dendrometrinae) were the sequential sister groups to Cardiophorinae + Negastriinae.

Douglas’ (2011) finding close relationship between the Cardiophorinae and the Negastriinae agrees with DNA sequence data-based results (Sagegami-Oba et al. 2007; Oba 2007; Kundrata and Bocak 2011 [with *Platiana* Schimmel, 1993 as sister to included Cardiophorinae, assigned to Dimini]; and Kundrata et al. 2016). Douglas (2011) also found strong support for Dendrometrinae: Hypnoidini as sister to Cardiophorinae + Negastriinae + *Tropihypnus*. This study uses Negastriinae, Hypnoidini, and *Tropihypnus* as outgroups for phylogenetic analysis of the Cardiophorinae, as the taxa identified as most likely (Douglas 2011) to render the Cardiophorinae non-monophyletic. Cardiophorine monophyly has not been demonstrated through analyses of larval or adult morphology to date, and requires testing.

## Research plan

No one has phylogenetically tested hypotheses about the membership or internal groupings of the Cardiophorinae. Additionally, no work including keys and diagnoses for all cardiophorine genera has been published since Schwarz (1906). Thirteen new genera, six subgenera and hundreds of species have been described since Schenkling’s (1925) catalog. For these reasons, it is difficult to identify many cardiophorines and the current nomenclature is unlikely to reflect evolutionary history. Additionally, inconsistencies in the use of genus level names also make genus level identifications difficult using literature alone.

I present here the only phylogenetic analysis of the Cardiophorinae to date, using 80 exemplar-species including much of the available morphological variation. These include 56 species from 27 of 29 described cardiophorine genera. The type species of 27 genus-level cardiophorine taxa and 20 outgroup taxa were included to ensure that included species truly represent named genera. Some additional morphologically-divergent or geographically distant members of genera are added as preliminary tests of generic monophyly. Outgroups represented most elateroid taxa most expected to confound elaterid monophyly. This study also tests the hypothesis that *Nyctor* Semenov-Tian-Shanskij & Pjatakova, 1936 is sister to the remainder of the Cardiophorinae, and thereby also testing the validity of subfamily Nyctorini.

Objectives of this study are: to test the monophyly of Cardiophorinae, its tribes, and genera. These results are used to redescribe the Cardiophorinae and its tribes and provide keys and diagnoses to define all included genera. Taxa are transferred as required to reflect phylogenetic findings and accepted taxonomic concepts.

## Materials and methods

### Taxon sampling and Specimens examined

Specimens examined for morphological coding belonged to 29 insect collections (Table 1). Codens listed here follow Arnett et al. (1993), except where collections preferred other codens. Among these specimens were 61 primary types, or paratype specimens, representing 41 species (Appendix III). Appendix III also includes lectotype designations for 29 species. These are designated to fix generic concepts and to ensure their universal and consistent interpretation. Types of 307 more species, which were not coded for phylogenetic analysis, were photographed at NHM (London), ISNB and MNHN. Types of 85 more North American cardiophorine species (listed in Douglas 2003) were also examined to ensure taxon sampling reflected much of the group’s morphological variation and assess new taxonomic placements.

Non-type specimens were identified by comparison with types (types were examined for 40 species) or specimens identified by experienced workers (three species, Appendix I). All non-type specimens examined were labelled with unique identifier numbers (Appendix II). Three distinctive undescribed species were included to better represent the Cardiophorinae. All identifications of non-types were evaluated using published keys and descriptions (Appendix I), and five species were identified using literature alone. Information from type specimens was often used in coding species. In most cases where a single name-bearing type did not already exist, a lectotype was designated for each species name (Appendix III).

**Table 1.** Codens for insect collections from which specimens were examined*.

**AMNH**	American Museum of Natural History, New York, New York, USA
**NHM**	The Natural History Museum [formerly British Museum (Natural History), BMNH], London, England
**BPBM**	Bernice P. Bishop Museum, Honolulu, Hawaii, USA
**CASC**	California Academy of Science, Department of Entomology, San Francisco, California, USA
**CMNC**	Canadian Museum of Nature, Ottawa, Canada
**CNCI**	Canadian National Collection of Insects, Arachnids, and Nematodes, Agriculture and Agri-Food Canada, Ottawa, Canada
**CUIC**	Cornell University Insect Collection, Department of Entomology, Cornell University, Ithaca, New York, USA
**DEBU**	University of Guelph Insect Collection, Guelph, Ontario, Canada
**DEIC**	Institut fur Pflanzenschutzforschung, Eberswalde, Germany
**FSCA**	Florida State Collection of Arthropods, Division of Plant Industry, Florida Department of Agriculture and Consumer Services, Gainesville, Florida, USA
**ISNB**	Institut Royal des Sciences Naturelles de Belgique, Brussels, Belgium
**LSUK**	Linnean Society, London, United Kingdom
**MCZC**	Museum of Comparative Zoology, Harvard University, Cambridge Massachusetts, USA
**MNHN**	Muséum National d’Histoire Naturelle, Entomologie, Paris, France
**MSUC**	Michigan State University, Department of Entomology Museum, East Lansing Michigan, USA
**MZHF**	University of Helsinki, Zoological Museum, Helsinki, Finland
**MZSP**	Museu de Zoologia da Universidade de São Paulo, São Paulo, Brazil
**NZAC**	New Zealand Arthropod Collection, Landcare Research, Auckland, New Zealand
**SANC**	South African National Collection of Insects, Pretoria, South Africa
**SEMC**	Snow Entomological Museum, Kansas State Biological Survey, University of Kansas, Lawrence, Kansas, USA
**TAMU**	Insect Collection, Department of Entomology, Texas A&M University, College Station, Texas, USA
**TARI**	Taiwan Agricultural Research Institute, Taichung, Taiwan
**UCMP**	University of California Museum of Palaeontology, Berkeley, California, USA
**USNM**	United States National Museum (Natural History), Washington, District of Columbia, USA
**ZMAS**	Russian Academy of Sciences, Zoological Institute, St. Petersburg, Russia
**ZMHB**	Museum für Naturkunde der Humboldt-Universität, Berlin, Germany
**ZMUC**	University of Copenhagen, Zoological Museum, Copenhagen, Denmark
**ZMUM**	Zoological Museum of Moscow Lomonosov State University, Moscow, Russia

* MZHF, TARI, UCMP are depositories (or expected depositories) of type specimens unavailable for examination.

For this study, 51 ingroup and 26 outgroup species were coded for phylogenetic analysis (Appendices I, IV). Of these, 21 were missing genitalic data for one sex. For two species, material was unavailable for both the aedeagal and female genitalic characters. Non-genitalic characters were coded from male specimens, except for six species, for which only females were available. *Agrypnella* was coded using males of the type species *Agrypnella eburnea* Champion, 1895, and a female identified as *Agrypnella squamifer* (Candèze, 1895), although it is unknown whether these two species are really distinct. Some species for which some characters could not be examined were coded partially from literature (Calder 1996, Gurjeva 1966, Ôhira 1963). A generic catalog including references and synonymies was assembled for the Cardiophorinae (Revised Synonymy below) to clarify genus level nomenclature.

Ingroup taxa included 53 cardiophorine species, including 5 taxa found to be near Cardiophorinae by Douglas (2011) (*Pachyelater* Lesne, 1897 (Physodactylinae or Dendrometrinae); *Margogastrius* (Physodactylinae); *Negastrius americanus* (Horn, 1871, Negastriinae); *Teslasena* (Physodactylinae), and an undescribed species from New Zealand). The type species of 25 of 32 valid and subjectively synonymised genus level names for the Cardiophorinae were included to ensure accurate inclusion of named genera and subgenera (Revised Synonymy below). Four genus-level names (*Aptopus*, *Coptostethus* Wollaston, *Dicronychus*, *Triplonychoidus*) were represented by non-type species only, and five genus level taxa were entirely unavailable for inclusion (*Allocardiophorus* Ôhira, *Cardiophorellus*: subgenus Parapleonomus Cobos, *Cardiophorus*: subgenus Lasiocerus Buysson, *Mionelater* Becker [fossil], *Ryukyucardiophorus* Ôhira (examined post-analysis)). Taxon sampling was most comprehensive for the genera with species occurring on multiple continents.

The outgroup (24 taxa) includes subfamilies Negastriinae, Elaterinae, Agrypninae, and Dendrometrinae. Previous studies indicated the Negastriinae were the sister group to (Sagegami-Oba et al. 2007; Oba 2007; Kundrata and Bocak 2011; and Kundrata et al. 2016), or rendered paraphyletic by (Douglas 2011) the Cardiophorinae. Because of this, exemplars of the type species 15 of 30 world negastriine genera, and one non-type species, were included to further test cardiophorine monophyly. These genera include seven of Stibick’s (1971) eight unnamed genus groups. Three genera of Hypnoidini including the type genus *Hypnoidus* Dillwyn, 1829, and *Adrastus* Eschscholtz, 1829, were included because Douglas (2011) found these were the most likely sister groups to Cardiophorinae + Negastriinae. Type species of *Elater* Linnaeus, 1758; *Agriotes* Eschscholtz, 1829; *Athous* Eschscholtz, 1829; and *Agrypnus* Eschscholtz, 1829 were also included, although only *Elater* was formally defined as outgroup in the analyses to root the trees.

### Specimen preparation and examination

Specimens were relaxed for examination by placement in nearly boiling distilled water for 10–30 minutes. Wings were photographed in water under a cover slip on a glass microscope slide. Male and female genitalia were prepared and examined as outlined in Calder (1996). Specimens were examined using a Leica Wild M-10 dissecting microscope and all structures examined were photographed using an attached Nikon Coolpix 995 digital camera. Measurements were made using either an ocular micrometer or from digital photographs using Corel Photo-Paint 12 software. Drawings were made using these digital photographs. Vestiture has been omitted from drawings except where taxonomically informative. Structural terms follow Douglas (2011). Figures of single proximal sclerites of the bursa copulatrix are meant to be of the right-sclerite viewed as from inside the bursa (internal view), unless stated otherwise. Sclerites of the bursa copulatrix are illustrated in internal view unless lateral view is specified.

### Morphological character coding

Morphological characters were coded using majority coding of polymorphisms in order to use all available information and avoid bias. For qualitatively defined characters, majority coding was practiced by coding the character state most commonly observed in each species. For 27 quantitatively coded morphometric characters (Table 2), the value entered for each species was the mean of ratios of length measurements or mean angle. These values were ranked assigned to character-state bins “0” or “1” based on whether the measured value for that character for each species was above or below the median value for the character among all species.

**Table 2.** Phylogenetic characters. Morphological characters used for phylogenetic analysis. Quantitative characters are indicated by the term “Quantitative.”; described here are the measurements and ratios used to obtain data for quantitative coding. Length refers to the portion of the distance between two points parallel to the longitudinal axis of the specimen’s body (e.g., measurements a–c, Fig. 5). Species named in brackets are designated as references to typify character states. Reference species used to typify character states in Douglas (2011) were re-used here even where the reference species was not included in this study. O = ordered multistate character.

**1**	Antennae: 0) with 11 antennomeres [*Cardiophorus gramineus*]; 1) with 12 antennomeres [*Pityobius anguinus*]
**2**	Quantitative. Ratio of lengths of antennomeres: two: three
**3**	Quantitative. Ratio of lengths of antennomeres: four: three
**4**	Quantitative. Ratio of dimensions of antennomere 11: (length): (maximum height)
**5**	Antennomere 1 with dorsolateral carina: 0) absent [*Elater ferrugineus*]; 1) present [*Zorochros demustoides*]
**6**	Antennae with sensory elements beginning on antennomere: 0) 3 [*Cardiophorus gramineus*; 1) 4 [*Elater ferrugineus*]
**7**	Quantitative. Ratio of dimensions of antennal fossa: (width of fossa):(minimum distance from fossa to eye)
**8**	Head with area between antenna fossa and compound eye: 0) unsculptured [*Cardiophorus gramineus*]; 1) with carina connecting fossa and eye [*Esthesopus castaneus*], or with 2 pits with non-depressed area between them [*Cardiotarsus mjobergi*]; 2) with a single pit [*Aptopus agrestis*]
**9**	Mandibular apex: 0) unidentate (simple) [*Cebrio gigas*]; 1) bidentate [*Paracardiophorus musculus*] 2) tridentate [*Buckelater argutus*] (O)
**10**	Labrum: 0) evenly convex dorsally [*Cardiophorus gramineus*]; 1) character state not assigned; 2) flat [*Cebrio gigas*] or broadly concave [*Craspedostethus rufiventris*]
**11**	Supra-antennal carina with split next to eyes: 0) absent (Fig. 1) [*Elater ferrugineus*]; 1) present (Fig. 2) [*Cardiophorus gramineus*]
**12**	Quantitative. Ratio of dimensions of frontoclypeal region: (distance between supra-antennal carina and labrum at midline): (minimum distance between antennal fossae)
**13**	Frontoclypeal region with carinae from bases of mandibles extending meso-dorsad to supra-antennal carina: 0) absent [*Cardiophorus gramineus*]; 1) present [*Dicrepidius ramicornis*]
**14**	Fronto-clypeus at midline in side view, with concavity between antero-ventral edge (adjacent to labrum) and supra-antennal carina: 0) absent [*Agriotes sputator*]; 1) present [*Cardiophorus gramineus*]
**15**	Fronto-clypeus with crenulations on anterior margin: 0) absent [*Elater ferrugineus*]; 1) present [*Neoarhaphes americanus*]
**16**	Frons with mesal groove: 0) absent [*Elater ferrugineus*]; 1) present [*Negastrius americanus*]
**17**	Frons with supra-orbital groove: 0) absent [*Elater ferrugineus*]; 1) present (Figs 1, 2) [*Cardiophorus gramineus*]
**18**	Quantitative. (Ocular index) Ratio of dimensions of compound eyes: (maximum distance between outer edges of compound eyes)/ (minimum distance between inner edges of eyes)
**19**	Apical segment of maxillary palp: 0) securiform to subtriangular (apex truncate, widest near apex) [*Cardiophorus gramineus*]; 1) oblong-ovate (apex rounded, sides equally curved, widest near midlength) [*Aphricus australicus*]; 2) lanceolate [*Adrastus pallens*]; 3) character state not assigned; 4) bottle shaped (lageniform) [*Arhaphes diptychus*]
**20**	Apical segment of labial palp: 0) securiform or subtriangular [*Cardiophorus gramineus*]; 1) oblong-ovate [*Aphricus australicus*]; 2) lanceolate [*Adrastus pallens*]; 3) bottle shaped [*Arhaphes diptychus*]
**21**	Mentum: 0) without macrosetae [*Cardiophorus gramineus*]; 1) with macrosetae [*Elater ferrugineus*]
**22**	Pronotum with space between punctures on disc: 0) flat [*Cardiophorus gramineus*]; 1) with tubercles [*Zorochros demustoides*], or ridges [*Negastrius pulchellus*]
**23**	Pronotum with scale-like setae, *i.e.*, dorsoventrally compressed, and/or wider at midlength than at base: 0) absent [*Cardiophorus gramineus*]; 1) present [*Agrypnella eburnea*]
**24**	Head and pronotum with integument: 0) unicoloured [*Negastrius pulchellus*]; 1) with contrasting light and dark areas [*Cardiophorus gramineus*]
**25**	Pronotum with hind angles: 0) not truncate dorsally (Fig. 3) [*Cardiophorus gramineus*]; 1) truncate dorsally (Fig. 4) so only narrow hypomeral portion of hind angle is visible in dorsal view [*Cardiophorus cardisce* (Say)]; 2) notched dorsally with dorsal surface of pronotum reaching apex of hind angle [*Agrypnella eburnea*]
**26**	Pronotum with lateral edge of hind angle with tubercle: 0) absent [*Cardiophorus gramineus*]; 1) present [*Platynychus indicus*]
**27**	Hind angles of pronotum with dorsal carina (beside lateral pronotal carina): 0) absent [*Athous vittatus*]; 1) present, but not reaching anterior edge of pronotum [*Elater ferrugineus*]; 2) present, reaching anterior edge of pronotum [*Quasimus minutissimus*] (O)
**28**	Pronotum at middle of posterior edge with: 0) arcuate indentation between 2 apices [*Elater ferrugineus*]; 1) point or lobe between 2 apices (= 3 apices) [*Cardiophorus gramineus*]; 2) single arcuate lobe (= 1 apex) [*Athous vittatus*]
**29**	Pronotum with sublateral incisions: 0) absent, and longitudinal carinae absent [*Elater ferrugineus*]; 1) present, without longitudinal carinae [*Dicrepidius ramicornis*]; 2) present, and with longitudinal carinae [*Cardiophorus gramineus*] (O)
**30**	Pronotum with lateral carina: 0) reaching from anterior edge to posterior or lateral edge of hind angle [*Elater ferrugineus*]; 1) absent anteriorly (ventrad of edge of pronotum in some) [*Cardiophorus gramineus*]
**31**	Pronotum extending laterally beyond lateral carina: 0) for entire length (Figs 3, 4) [*Cardiophorus gramineus*]; 1) not at all [*Elater ferrugineus*]; 2) only in anterior half [*Agriotes sputator*]; 3) only in posterior half [*Melanotus castanipes*]
**32**	Posterior edges of hypomeron mesad of hind angles: 0) with rectangular or semicircular indentations (Fig. 3) [*Cardiophorus gramineus*]; 1) straight or shallowly sinuate (Fig. 4) [*Elater ferrugineus*]; 2) convex [*Prosternon tessellatum*]
**33**	Hypomera with loop shaped carinae near procoxae: 0) absent [*Elater ferrugineus*]; 1) present [*Arhaphes diptychus*]
**34**	Prosternum with anterior edge: 0) short, exposing labium [*Physodactylus henningi*]; 1) not short, produced as lobe, concealing labium when head not extended [*Cardiophorus gramineus*]
**35**	Prosternum with anterior edge at midline: 0) arcuate [*Elater ferrugineus*]; 1) notched [undescribed species, New Zealand, transferred in this study to *Aphricus*]; 2) with tubercle [*Esthesopus castaneus*]
**36**	Prosternum with sides near midlength: 0) straight [*Cardiophorus gramineus*] to weakly concave [*Elater ferrugineus*]; 1) convex [*Negastrius pulchellus*]
**37**	Pronotosternal sutures with anterior ends: 0) closed [*Cardiophorus gramineus*]; 1) open, produced into grooves large enough to guide, but not conceal, antennae [*Agriotes sputator*]; 2) excavated deeply enough to conceal part of length of antennae [*Agrypnus murinus*] (O)
**38**	Pronotosternal sutures: 0) not interrupted, pronotum and prosternum not fused [*Elater ferrugineus*]; 1) partly interrupted, pronotum and prosternum fused [*Arhaphes diptychus*]
**39**	Prosternal process with “V” shaped carina on sides of ventral surface in ventral view: 0) absent or interrupted [*Elater ferrugineus*]; 1) complete, connected basally to carinae surrounding procoxal cavities [*Paracardiophorus musculus*]
**40**	Quantitative. Ratio of dimensions of prosternal process: (length of portion of prosternal process extending posterad of procoxae): (maximum length of exposed part of procoxae in ventral view)
**41**	Quantitative. Ratio of dimensions of prosternal process (Fig. 5, c/a): (length from posterior edge of procoxae to ventral apex of prosternal process): (length from posterior edge of procoxae to dorsal apex of prosternal process)
**42**	Quantitative. Ratio of dimensions of prosternal process (Fig. 5, b/a): (length from posterior edge of procoxae to posterior end of prosternal process, halfway between dorsal and ventral apices): (length from posterior edge of procoxae to dorsal apex of prosternal process)
**43**	Quantitative. Ratio of dimensions of Prosternal process (Fig. 5, d/a): (vertical distance between dorsal and ventral apices of prosternal process): (length from posterior edge of procoxae to dorsal apex of prosternal process)
**44**	Prosternal process (anterad of ventral apex) with angle between ventral surface and ventral surface of middle of prosternum anterior to procoxae: 0) less than 30° (horizontal) [*Elater ferrugineus*]; 1) more than 30° (inclined dorsally) [*Dima elateroides*]
**45**	Procoxal cavities: 0) open posteriorly [*Elater ferrugineus*]; 1) fully closed posteriorly [*Paracardiophorus musculus*]
**46**	Protibiae near apex with posterior surface: 0) convex, not modified for digging [*Cardiophorus gramineus*]; 1) flattened, concave, or broadened apically, apparently modified for digging [*Cebrio gigas*]
**47**	Pronotum with punctures on disc: 0) elongate [*Athous vittatus*]; 1) not elongate [*Cardiophorus gramineus*]; 2) absent, setae on tubercles [*Anelastes druryi*]
**48**	Scutellum with middle of anterior edge: 0) straight [*Pyrophorus noctilucus*] or convex [*Elater ferrugineus*] (Fig. 6); 1) broadly concave [*Athous vittatus*] (Figs 7, 9); 2) abruptly emarginate [*Cardiophorus gramineus*] (Fig. 8)
**49**	Scutellum with anterolateral edges: 0) convex throughout [*Cardiophorus gramineus*]; 1) straight [*Athous vittatus*], or concave posterior to anterolateral corners [*Agriotes sputator*]
**50**	Scutellum with posterolateral edges: 0) with straight [*Cardiophorus gramineus*] (Fig. 8) or concave portion [*Rivulicola variegatus* (Macleay)], meeting at acute to obtuse angle; 1) convex throughout [*Elater ferrugineus*] evenly rounded or with convex sides meeting at an obtuse angle; 2) with straight portion, apex truncate [*Blaiseus bedeli*] (Fig. 9); 3) concave, apex bilobed [*Negastrius americanus*] (Fig. 7)
**51**	Sides of mesosternal cavity with antero-ventral angles in lateral view: 0) prominent and rounded (Fig. 14), [*Cardiophorus gramineus*]; 1) prominent and angulate, acute to 140° (concave ventrad of antero-ventral angle in some, appearing able to fit procoxae) [*Esthesopus castaneus*]; 2) 160°–180° and hidden by mesocoxae in side view [*Elater ferrugineus*]; 3) excavated in amphitheatre shape [*Arhaphes diptychus*]
**52**	Sides of mesosternal cavity posterior to anterior edge of mesocoxae in ventral view: 0) U shaped [*Cardiophorus gramineus*]; 1) straight, forming a V [*Elater ferrugineus*]; 2) with anteromesal projection [*Arhaphes diptychus*]; 3) not assigned; 4) with 3 obtuse angles and 4 sides [*Cardiophorus convexulus*]
**53**	Sides of mesosternal cavity anterior to mesocoxae: 0) sinuate [*Cardiophorus gramineus*]; 1) straight (parallel or anteriorly convergent) [*Semiotus furcatus*]
**54**	Anterior edge of mesosternum in ventral view: 0) concave lateral to anterior protrusions of mesosternal fossa [*Elater ferrugineus*]; 1) convex lateral to anterior protrusions of mesosternal fossa [*Negastrius pulchellus*], or evenly convex, uninterrupted by mesosternal fossa [*Zorochros demustoides*]
**55**	Mesepisternum with anterior projection of anteromesal corners extending beyond junction with mesosternum: 0) concave mesally forming an acute point (Fig. 5, upper left) [*Cardiophorus gramineus*]; 1) rounded (Fig. 11) [*Elater ferrugineus*] to obtusely angulate, but not concave mesally [*Ampedus sanguineus*]
**56**	Mesepisternum with circular pit at anteromesal corner: 0) absent [*Elater ferrugineus*] (some with groove [*Athous vittatus*]); 1) present [*Agrypnus murinus*]
**57**	Angle of anterolateral corner of mesepisternum (angle between tangents of edge of sclerite at lowest part of concavity immediately mesad of angle, and an equidistant point on lateral edge): 0) approximately right angled (80°–100°, Fig. 10) without notch immediately mesad of angle [*Cardiophorus gramineus*]; 1) approximately right angled with notch immediately mesad of corner [*Tropihypnus bimargo*]; 2) obtuse or evenly curved (Fig. 12) [*Ampedus sanguineus*]
**58**	Quantitative. Ratio of dimensions of mesepimeron: (maximum width, measured parallel to anterior edge)/ (maximum length, perpendicular to width)
**59**	Mesotrochantin: 0) visible [*Elater ferrugineus*], or partly concealed by mesosternum; 1) not visible [*Cardiophorus gramineus*]
**60**	Mesocoxal cavity: 0) open to both mesepimeron and mesepisternum (Fig. 11) [*Elater ferrugineus*]; 1) open to mesepimeron only (Fig. 12) [*Agriotes sputator*]; 2) closed to mesepimeron and mesepisternum by extension of mesosternum (Fig. 13) [*Cardiophorus gramineus*] (O)
**61**	Quantitative. Ratio of dimensions of midleg: (trochanter length): (femur length)
**62**	Quantitative. Ratio of dimensions of exposed portion of metepisternum: (length): (width). Width measured at midlength, not including portion covered by closed elytra
**63**	Metasternum behind mesocoxal cavities: 0) without postcoxal lines [*Elater ferrugineus*]; 1) with postcoxal lines, either arc-shaped [*Lissomus bicolor*] or loop-shaped [*Quasimus minutissimus*]
**64**	Metasternum with width ratio of lateral carina (raised flattened lateral edge of metasternum, Fig. 14, c) to distance between it and carina surrounding mesocoxal cavities at level of posterior third of mesocoxae (Fig. 14, d): 0) less than 0.5 [*Cardiophorus gramineus*]; 1) greater than 0.5 [*Quasimus minutissimus*]
**65**	Edge of elytra in dorsal view between anterior-most point and humeral angle: 0) sinuate or with tubercle [*Cardiophorus gramineus*] (Fig. 15); 1) arcuate (Fig. 16) or straight [*Elater ferrugineus*]; 2) cleft by anterior extension of striae, with small tubercles lateral to cleft [*Rivulicola variegatus*]
**66**	Number of puncture rows or striae on elytra: 0) 0 (absent on basal half) [*Cebrio gigas*]; 1) 9 [*Elater ferrugineus*]
**67**	Elytra with intervals 1–8 on basal third: 0) flattened or rounded [*Elater ferrugineus*]; 1) partly or completely costate [*Negastrius pulchellus*]
**68**	Elytra with apical half of intervals 1–8: 0) flattened or rounded throughout[*Elater ferrugineus*]; 1) with at least some costate [*Aphricus californicus*]
**69**	Elytra with apical half of interval 9: 0) flattened or rounded [*Elater ferrugineus*]; 1) costate [*Triplonychoidus trivittatus* (Champion)]
**70**	Elytra: 0) without spots or markings [*Elater ferrugineus*] (some with basal markings or longitudinal stripe); 1) with distinct transverse markings or spots not confluent with anterior edge [*Negastrius pulchellus*]
**71**	Upper edge of elytral epipleura: 0) with minute regular serrations [*Cardiophorus gramineus*]; 1) without serrations [*Elater ferrugineus*]
**72**	Elytral apex with punctures at least 1.5X diameter of largest on anterior half: 0) absent [*Elater ferrugineus*]; 1) present, single [*Paracardiophorus subcruciatus*], or multiple [*Agrypnella eburnea*]
**73**	Elytron with apical shelf like extension: 0) absent [*Elater ferrugineus*]; 1) present [*Cardiophorus nigratissimus*].
**74**	Hind wing: 0) with venation well developed, wing area greater than elytral area [*Elater ferrugineus*]; 1) with veins weakly sclerotised, wing area less than half of elytral area [*Dima elateroides*].
**75**	Hind wing membrane: 0) not notched in anal area [*Elater ferrugineus*]; 1) notched in anal area (between AA3+4 and AP) [*Negastrius pulchellus*]
**76**	Hind wing with apical concavity: 0) absent [*Elater ferrugineus*]; 1) present [*Berninelsonius hyperboreus*]
**77**	Quantitative. Ratio of dimensions of hind wing, radial cell: (length): (width)
**78**	Quantitative. Ratio of dimensions of Hind wing: proximal, posterior angle of radial cell (measured in degrees) between tangents of: anterior edge of cell, at 1/3 distance from posterior angle to anterior edge of cell; and posterior edge of cell at an equal distance from angle)
**79**	Hind wing with vein AA3: 0) joining CuA posterior to divergence from AA4 [*Elater ferrugineus*]; 1) joining CuA at divergence from AA4 [*Esthesopus castaneus*]; 2) joining CuA, AA4 not continuing posteriorly [*Negastrius pulchellus*]
**80**	Hind wing with wedge cell: 0) present (Fig. 17, contains letters CuA) [*Elater ferrugineus*]; 1) absent (Fig. 18) [*Cardiophorus gramineus*]
**81**	Hind wing vein MP3 and MP4 separating: 0) distal to intersection with CuA1 [*Cardiophorus gramineus*]; 1) proximal [*Elater ferrugineus*] to or at same level as [*Macropogon piceus*] intersection with CuA1. (CuA1 interrupted in some)
**82**	Hind wing with vein CuA1: 0) uninterrupted (Fig. 18) [*Elater ferrugineus*]; 1) interrupted or not reaching MP3+4 or MP4 [*Cardiophorus gramineus*]
**83**	Hind wing with CuA1 0) not forked [*Elater ferrugineus*]; 1) forked at junction with MP3+4 forming additional closed cell [*Blaiseus bedeli*] (Fig. 18)
**84**	Hind wing with vein MP3+4 with proximal extension of crossvein mp1+2-mp3+4: 0) present [*Elater ferrugineus*]; 1) absent (Figs 17, 18) [*Cardiophorus gramineus*]
**85**	Apex of hind wing with anterior field sclerotisation (Muona 1993): 0) absent [*Negastrius pulchellus*]; 1) single (Fig. 18) [*Elater ferrugineus*]; 2) double (Fig. 17) [*Adrastus pallens*] (O)
**86**	Apex of hind wing with median field (Muona 1993): 0) unsclerotised [*Hypnoidus riparius*]; 1) sclerotised (Figs 17, 18) [*Elater ferrugineus*]
**87**	Apex of hind wing with linear sclerites of posterior field (Muona 1993): 0) unsclerotised [*Hypnoidus riparius*]; 1) sclerotised (Figs 17, 18) [*Elater ferrugineus*]
**88**	Quantitative. Ratio of dimensions of metacoxal plate: (width): (length at widest point mesad of trochanter attachment)
**89**	Metacoxae with posterior excavation: 0) sufficient to cover at least 2/3 area of trochanter with hind legs withdrawn (Figs 19, 20) [*Cardiophorus gramineus*]; 1) insufficient to cover 2/3 of trochanter with hind legs withdrawn [*Athous vittatus*]
**90**	Metacoxal plate: 0) not reaching metepisternum, or shorter than 1/3 posterior width of metepisternum lateral to intersection with metepisternum (Fig. 20) [*Cardiophorus gramineus*]; 1) reaching more than halfway across metepisternum and longer than 1/3 posterior width of metepisternum lateral to intersection with metepisternum (Fig. 19) [*Aulonothroscus punctatus*]
**91**	Metacoxal plate with mesally directed hook: 0) absent [*Elater ferrugineus*]; 1) present [*Cardiohypnus mirabilis*]
**92**	Metatibia with number of apical spurs: 0) 2 [*Elater ferrugineus*]; 1) 0 [*Lissomus bicolor*]
**93**	Quantitative. Ratio of dorsal lengths of tarsomeres of hind leg: (1): (2)
**94**	Metatarsi with only the following tarsomeres lobed or lamellate: 0) none [*Elater ferrugineus*]; 1) not assigned; 2) 4 [*Cardiotarsus capensis* Candèze]; 3) 3, 4 [*Monadicus* sp.]; 4) 1, 2, 3 [*Athous vittatus*]
**95**	Metatarsal claws with basal setae: 0) absent [*Elater ferrugineus*]; 1) present, multiple in some [*Agrypnus murinus*]
**96**	Metatarsal claws with number of apices per side (including flange *sensu* Stibick 1971): 0) 1 [*Elater ferrugineus*]; 1) 2 [*Dicronychus cinereus* (Herbst)], (Fig. 21); 2) 3 or more [*Melanotus castanipes*]
**97**	Urosternites 3–6 with size of adjacent punctures: 0) approximately uniform [*Elater ferrugineus*]; 1) of 2 size classes [*Paracardiophorus musculus*]
**98**	Urosternites 3–7 with elongate punctures: 0) absent [*Athous vittatus*] or limited to urosternites 6 and 7; 1) throughout [*Ampedus sanguineus*]
**99**	Lateral edges of urosternites 3–7 with serrations: 0) present on at least some [*Cardiophorus gramineus*]; 1) absent [*Elater ferrugineus*]
**100**	Urosternites 6 and 7 with multiple longitudinal ridges: 0) absent [*Elater ferrugineus*]; 1) present [*Tropidiplus tellinii*]
**101**	Urosternite 7 (and in some 6) with second carina mesad of lateral carina: 0) absent [*Cardiophorus gramineus*]; 1) present [*Tropidiplus tellinii*]
**102**	Male urosternite 8 with mesal third of anterior sclerotised band (anterior margin between bases of anterior lobes): 0) straight, bisinuate [*Arhaphes diptychus*] or arcuate [*Paracardiophorus musculus*], width uniform or interrupted mesally [*Cardiophorus gramineus*]; 1) anteriorly angulate, width uniform [*Zorochros demustoides*]; 2) broadened mesally, anteriorly arcuate [*Ampedus sanguineus*], interrupted laterally in some [*Physorhinus erythrocephalus* (Fabricius)]; 3) indistinguishable from posterior lobes, because sclerite of uniform length across width, or longest mesally [*Aphricus australicus*]; 4) indistinguishable from posterior lobes, because posterior lobes connate basally [*Pachyelater madagascariensis*]; 5) absent (entirely unsclerotised) although posterior lobes present [*Dicrepidius ramicornis*]
**103**	Sclerotised basal band of male urosternite 8: 0) not extending laterally beyond posterior lobes (*i.e.*, posterior edge of sclerite convex from apex of lobes to sides of sclerite) [*Elater ferrugineus*]; 1) extending laterally beyond slope of lobes (posterior edge of sclerite sinuate from apex of lobes to sides of sclerite) [*Paracardiophorus musculus*]
**104**	Tergite and sternite of male abdominal segment 9 articulated at: 0) sides [*Elater ferrugineus*]; 1) base [*Blaiseus bedeli*]
**105**	Male urosternite 9 with anterior end: 0) not pointed [*Elater ferrugineus*]; 1) pointed [*Paradonus pectoralis*]
**106**	Quantitative. Ratio of lengths of male urotergites: (9 [at point of greatest length])/ (10 [at midline])
**107**	Male urotergites 9 and 10: 0) not fused mesally [*Elater ferrugineus*]; 1) fused mesally [*Fleutiauxellus maritimus* (Curtis)]
**108**	Male urotergite 9 with shape of apical emargination: 0) “U” [*Elater ferrugineus*]; 1) between “U” and “V” [*Ampedus sanguineus*]; 2) “V” [*Agrypnus murinus*]
**109**	Aedeagus with antero-dorsal (basal) concavity of phallobase in dorsal view: 0) simple (Fig. 24) [*Elater ferrugineus*]; 1) bisinuate (with mesal convexity) [*Cardiophorus gramineus*], (Fig. 84)
**110**	Aedeagus with posterior (apical) emargination of phallobase: 0) deep and evenly concave [*Elater ferrugineus*]; 1) reduced, concavity nearly absent [*Pityobius anguinus*]; 2) produced mesally [*Athous vittatus*]
**111**	Quantitative. Ratio of dimensions of aedeagus: (width of posterodorsal concavity of phallobase (concavity at apical end of phallobse) [0 if absent]): (maximum width of phallobase)
**112**	Quantitative. Ratio of dimensions of aedeagus: (length of phallobase): (maximum width of phallobase)
**113**	Quantitative. Ratio of dimensions of median lobe of aedeagus: (length of apical portion [portion posterad of concavity between basal struts]): (length of concavity between basal struts)
**114**	Quantitative. Ratio of dimensions of aedeagus: (length of entire median lobe [including basal struts]): (maximum width of phallobase)
**115**	Quantitative. Ratio of dimensions of aedeagus: (length of parameres): (maximum width of phallobase)
**116**	Quantitative. Ratio of dimensions of aedeagus: (length of overlap between phallobase and parameres): (maximum width of phallobase)
**117**	Aedeagus with parameres: 0) articulated with median lobe basally, pivoting at base [*Elater ferrugineus*] (Fig. 23); 1) not assigned; 2) articulated apicad of base, bases fused together into a tube (parameres rigid in some) [*Cardiophorus gramineus*], (Figs 24, 139)
**118**	Parameres in species with parameres articulated beyond bases with abrupt narrowing immediately apicad of point of articulation: 0) absent [*Cardiophorus gramineus*], (Fig. 24); 1) present [*Cardiophorus cardisce*]; ?) parameres articulated basally
**119**	Aedeagus with parameres with pre-apical or apical expansions: 0) absent [*Elater ferrugineus*]; 1) present, lateral or ventral [*Agriotes sputator*]; 2) not assigned; 3) present, mesal side [*Cardiophorus luridipes* Candèze]
**120**	Parameres with ratio of lateral width to dorsoventral depth of free portion: 0) less than 2 [Cardiophorus gramineus]; 1) greater than 2 [Cardiophorus luridipes]
**121**	Parameres with number of apices: 0) 1 [*Elater ferrugineus*], (Fig. 23); 1) 2 [*Blaiseus bedeli*], (Fig. 25)
**122**	Parameres in dorsal view with profile of mesal edge of apices: 0) not concave [*Elater ferrugineus*]; 1) concave [*Dicrepidius ramicornis*]
**123**	Parameres with number of setae on each: 0) 0 [*Agriotes sputator*]; 1) 1 [*Hypnoidus riparius*], (Fig. 23); 2) 2 [*Cardiophorus gramineus*], (Fig. 24); 3) 3 or more [*Elater ferrugineus*], (Fig. 25) (O)
**124**	Parameres with setae: 0) restricted to sides, basad of apicolateral expansions [*Dicronychus cinereus*] or of apical arc in species without expansions [*Esthesopus parcus* Horn]; 1) restricted to apex, apicad of apicolateral expansions if present [*Athous vittatus*] or to apical arc if expansions absent [*Selonodon speratus*]; 2) both apicad and basad of apicolateral expansions [*Pyrophorus noctilucus*] or apical arc; ?) with position uncertain because apical arc not distinguishable and apicolateral expansions absent
**125**	Parameres with apices: 0) opaque or otherwise not abruptly translucent [*Cardiophorus gramineus*]; 1) abruptly translucent [*Dicrepidius ramicornis*]
**126**	Aedeagus with apex of median lobe in lateral view: 0) without globular expansion [*Elater ferrugineus*], straight or down turned; 1) with globular expansion [*Esthesopus castaneus*], straight or down turned; 2) without expansion, but abruptly upturned [*Agrypnella eburnea*]
**127**	Female urosternite 8 with lateral sclerotisations: 0) joined at or near apex [*Elater ferrugineus*]; 1) not joined apically [*Zorochros demustoides*]
**128**	Female urosternite 8 with lateral sclerotisation: 0) joined to sclerotised spiculum [*Elater ferrugineus*]; 1) separated from sclerotised spiculum by membranous tissue or intermittent sclerotisation [*Cardiophorus gramineus*]
**129**	Female urosternite 8 with spiculum: 0) present [*Elater ferrugineus*]; 1) absent [*Pachyelater madagascariensis*]
**130**	Ovipositor with sclerites of coxites: 0) narrow, occupying less than 1/2 width of coxites at midlength [*Cardiophorus gramineus*], (Fig. 26); 1) heavy, occupying more than half of width of coxites at midlength [*Elater ferrugineus*]
**131**	Quantitative. Ratio of dimensions of ovipositor: (paraproct length): (coxite length)
**132**	Ovipositor with apical stylus: 0) absent [*Cardiophorus gramineus*]; 1) present [*Agriotes sputator*], (Fig. 26)
**133**	Vagina with colleterial glands: 0) absent [*Cardiophorus gramineus*], (Fig. 27) or small, less than hemispherical [*Rivulicola variegatus*]; 1) hemispherical, not pedunculate [*Elater ferrugineus*]; 2) globose, and pedunculate [*Athous vittatus*] (O)
**134**	Vagina with colleterial glands attached: 0) on either side of common oviduct [*Elater ferrugineus*]; 1) anterior to common oviduct [*Anisomerus sylvestris*]; 2) posterior to common oviduct [*Athous vittatus*]
**135**	Bursa copulatrix with spermathecal gland duct opening (or shared spermatheca-spermathecal gland duct): 0) away from base of anterior blind sac [*Cardiophorus gramineus*]; 1) adjacent to base of blind sac [*Paracardiophorus musculus*], (Fig. 27)
**136**	Spermathecal gland (or shared) duct opening: 0) at distal (anterior) end of bursa copulatrix [*Elater ferrugineus*], (Fig. 27); 1) between vagina [*Esthesopus parcus*] and midlength of bursa [*Cardiophorus gramineus*]
**137**	Bursa copulatrix with spermathecal gland duct attached to: 0) main bursa [*Cardiophorus gramineus*], (Fig. 27), or extension of bursa: non-tubular [*Cebrio gigas*], or tubular [*Elater ferrugineus*]; 1) spermatheca [*Anelastes druryi*] or shared duct [*Oedostethus femoralis*]
**138**	Female spermathecal gland duct with single row of diverticulae: 0) present [*Cardiophorus gramineus*]; 1) absent (but duct present) [*Elater ferrugineus*]
**139**	Number of coil-type spermathecae: 0) 0 [*Cardiophorus gramineus*]; 1) 1 [*Agriotes sputator*]; 2) 2 [*Pyrophorus noctilucus*]
**140**	Female with number of sclerotised capsule-type spermathecae: 0) 0 [*Cardiophorus gramineus*]; 1) 1 [*Elater ferrugineus*]
**141**	Bursa copulatrix with blind anterior sac attached near: 0) apex of bursa [*Cardiophorus gramineus*], (Fig. 27); 1) base of bursa (near median oviduct) [*Onichodon orchesoides*]
**142**	Bursa copulatrix with number of blind pedunculate sacs: 0) 0; 1) 1 [*Cardiophorus gramineus*], (Fig. 27); 2) 2 [*Horistonotus simplex*]
**143**	Bursa copulatrix with number of non-pedunculate blind tubular extensions: 0) 0 [*Cardiophorus gramineus*], (Fig. 27); 1) 1 [*Elater ferrugineus*]. E.g., two extensions in lower- left corner of Calder`s (1996) illustration of bursa of *Ophidius elegans* Candèze, 1863
**144**	Bursa copulatrix with free spines (*i.e.*, not part of sclerite with multiple spines): 0) absent [*Cardiophorus gramineus*], (Fig. 27); 1) present [*Melanotus castanipes*]
**145**	Bursa copulatrix with free spines: 0) not combined into paired, discrete, ovoid patches [*Melanotus castanipes*]; 1) present and combined into paired discrete ovoid patches, with individual spines separated by membranous tissue [*Exoeolus rufescens*]; 2) present and combined into flexible paired discrete ovoid patches, but with some spines partially fused [*Physorhinus erythrocephalus*] (O)
**146**	Bursa copulatrix with rugose, spineless, partially sclerotised patches: 0) absent [*Cardiophorus gramineus*], (Fig. 27); 1) present [*Horistonotus simplex*] (Fig. 162)
**147**	Bursa copulatrix with single dorsal and ventral sclerites both: 0) absent [*Negastrius pulchellus*], (Fig. 27); 1) present [*Oedostethus femoralis*]
**148**	Bursa copulatrix with single dorsal and ventral sclerites both: 0) bilaterally symmetrical [*Oedostethus femoralis*]; 1) not symmetrical [*Quasimus minutissimus*]
**149**	Bursa copulatrix, of species with single symmetrical dorsal and ventral sclerite of bursa copulatrix, with ventral sclerite: 0) fully sclerotized at midline [*Oedostethus femoralis*]; 1) divided or weakly sclerotised along midline [*Neoarhaphes americanus*]
**150**	Bursa copulatrix with single dorsal and ventral sclerites: 0) both not ring-like, opaque at center [*Oedostethus femoralis*]; 1) with at least dorsal sclerite ring-like, and transparent at center [*Arhaphes diptychus*]
**151**	Quantitative. (meristic) Ratio of dimensions of bursa copulatrix with paired proximal sclerites: (count # of rows of spines, including outer row, which surrounds most of sclerite in most species)
**152**	Bursa copulatrix with proximal sclerites (defined as pair of spine-bearing sclerites closest to vagina (for species with only 1 pair of sclerites: defined as proximal if not surrounding base of spermathecal gland duct): 0) absent [*Craspedostethus rufiventris*]; 1) present [*Cardiophorus gramineus*], (Fig. 27); 2) absent but with 3 asymmetrical spine-bearing sclerites [*Agriotes sputator*]
**153**	Bursa copulatrix with points of proximal sclerites: 0) simple [*Cardiophorus gramineus*], (Fig. 83); 1) pinnate, with spines on spines [*Cardiotarsus capensis*], (Fig. 118)
**154**	Bursa copulatrix with proximal sclerites: 0) ovoid [*Cardiophorus gramineus*], (Fig. 28); 1) bilobed [*Paraplatynychus mixtus*], (Fig. 29); 2) parallel sided [*Globothorax chevrolati*], (Fig. 30); 3) with multiple acute lobes [*Esthesopus parcus*], (Fig. 31)
**155**	Bursa copulatrix with placement of proximal sclerites: 0) symmetrical [*Cardiophorus gramineus*]; 1) asymmetrical [*Cardiophorus brunnipennis*]
**156**	Quantitative. Ratio of dimensions of bursa copulatrix: (length of largest spines of a proximal sclerite [measured as smallest possible distance between a line connecting 2 adjacent apices and the deepest part of the concavity between them]): (length of sclerite) (Fig. 28)
**157**	Bursa copulatrix with paired distal sclerites (pair farthest from vagina, at base of spermathecal gland duct or shared duct): 0) absent [*Elater ferrugineus*]; 1) present [*Cardiophorus inflatus*], fused together in some at wall of bursa [*Cardiophorus gramineus*] (Figs 32–35)
**158**	Bursa copulatrix with two distal sclerites: 0) separate [Cardiophorus inflatus], (Fig. 32); 1) fused together (at wall of bursa) into a “U” [Cardiophorus gramineus], (Fig. 33); 2) fused at both ends as a loop [*Aptopus pullatus*]
**159**	Bursa copulatrix with distal sclerites: 0) smooth [*Cardiophorus gramineus*], (Fig. 33); 1) rugose [*Dicronychus cinereus*], (Fig. 91)
**160**	Bursa copulatrix with distal sclerites: 0) flexible and at least in part weakly sclerotised and membranous [*Cardiophorus convexus*] (Fig. 96); 1) entirely sclerotised [*Cardiophorus gramineus*], (Fig. 33)
**161**	Bursa copulatrix with tube-like sclerotisation of base of spermathecal gland duct: 0) absent [*Cardiophorus gramineus*], (Fig. 33); 1) present, without paired plate like appendages [*Paracardiophorus musculus*], (Fig. 35); 2) present, with paired plate like appendages [*Cardiophorus cardisce*], (Fig. 34) (O)
**162**	Habitat: 0) restricted to riparian areas [*Negastrius pulchellus*]; 1) not restricted to riparian areas [*Elater ferrugineus*] (Not used to infer phylogeny)
**163**	Bursa copulatrix with multiple parallel linear sclerites: 0) absent [*Elater ferrugineus*]; 1) present [*Athous vittatus*]

Morphological character selection and coding were performed together. All observed variation was evaluated as a potential character source following one procedure. To be considered suitable, variation between homologous structures must allow diagnosis between at least one pair of species. All 136 characters that could not be described as length ratios, or counts, were treated qualitatively as binary or multistate characters. Qualitative characters included the presence or absence of structures, or objective shape descriptors (e.g., notched vs. uniformly convex). An exemplar species was assigned for each qualitatively defined character state in an effort to produce repeatable, standardized character state definitions (many follow Douglas 2011). Following these criteria, all qualitative characters identified as showing non-overlapping variation between at least two species were considered for possible use. Subsequent characters with apparent developmental or genetic non-independence were then excluded. Autapomorpic characters were also encoded because they provide branch length information for Bayesian analysis and diagnostic characters.

Phylogenetic analysis was conducted using 163 characters (Table 2), of which 27 were coded quantitatively (into binary pairs) and 136 qualitatively. These characters included 376 character states (after binary coding of quantitative characters), of which 40 were autapomorphic (Table 2). Qualitatively coded characters 6, 9, 22, 27, 29, 37, 44, 60, 85, 123, 133, 145, and 161 were treated as ordered multistate characters. Three characters, common to many fossorial Elateridae (Douglas 2011), were omitted from the analyses presented here, to avoid phylogenetic bias due to convergent evolution. These were characters 9, 34 and 46 which included the following character states apparently associated with fossorial adults: mandibular apex unidentate; prosternum with anterior edge short, exposing labium; and protibiae near apex with posterior surface flattened, concave, or broadened apically, apparently modified for digging. Character 162, riparian habitat association was also excluded from the analysis. Analyses including these characters (not presented) had similar topologies to those with them omitted but Bayesian posterior probability values (PP) were lower throughout the tree, supporting the hypothesis of convergence.

### Choice of optimality criteria

Although both parsimony and Bayesian analyses (as implemented by MrBayes v.3.1.2, Huelsenbeck and Ronquist 2001, Ronquist and Huelsenbeck 2003, using the model by Lewis 2001) were used here to infer phylogeny, results of the model-based Bayesian analyses were preferred for taxonomic inference. The major expected advantage of using Bayesian analyses for morphological data is that the Mkv model of Lewis uses branch length information while parsimony does not. Empirically, Wiens (2005) found that accuracy of Bayesian analyses equalled or exceeded that of parsimony. Parsimony analyses were also performed because parsimony remains widely accepted.

### Model selection

Bayes factors were used, as outlined by Sikes et al. (2006), to infer which of two evolutionary models best fit the data. These were the generalized Jukes-Cantor model for *k* states (Mkv), corrected for acquisition bias (Lewis 2001), with or without gamma distributed rate variation between characters (Mkv vs. Mkv + Γ). A Bayes factor of 1118 (2X ln L = -12837 for -Gamma and = -11719 for +Gamma) showed strong support (assessed as outlined by Kass and Raftery 1995) for models including gamma-distributed rate variation between characters over models that did not include gamma variation.

### Phylogenetic analyses

Phylogenetic analysis was performed using both parsimony and Bayesian criteria. For Bayesian analyses, prior probability distributions were at default values of MrBayes. Gamma distribution was approximated using the default setting of four rate classes. Settings for likelihood parameters used were Mkv (nst=1, coding=variable) and Mkv (nst=1, coding=variable, rates=gamma). Searches began with randomly selected starting trees and were run for 8 million cycles (until the average standard deviation of split frequencies between four parallel runs was below 0.01). Samples of trees from the MCMC chain were taken every 100 cycles, which resulted in 80 thousand trees. All but the first 20 thousand trees were used to compute a majority rule consensus tree assigning posterior probabilities of tree topology. The matrix was analysed three times to test repeatability. Because these differed slightly, the analysis with the highest average harmonic mean log likelihood was used for phylogenetic inference.

Parsimony analysis was performed using PAUP* (Swofford 2001). The heuristic search procedure was used with 1000 random replications of stepwise-addition, with the branch-swapping algorithm (maxtrees set to auto-increase, multrees option in effect, Appendix 5). Bootstrap values were generated through 1000 replicates of bootstrapping using the same settings but with maxtrees set to 1000 and the number of replicates of stepwise addition reduced to 10 to reduce processing time. Decay index scores were calculated using PRAP (Müller 2004) for decay analysis of a strict consensus of all trees found in initial parsimony analysis.

Zander (2004) found that Bayesian posterior probability (PP) values are predictably liberal at branch lengths typical for morphological studies (fewer than 35 changes). Because of this bias, PP values and other branch support metrics were corrected using Zander’s table 4 when assessing clade credibility for hypothesis testing.

### Tests of monophyly

Testing hypotheses of monophyly was done by determination of the PP of the focal clade. The hypotheses tested are either ones stated explicitly as such, or ones implied by the description of taxa.

### Generic diagnoses, figures, and key to genera

A key to the genera of cardiophorine, and corresponding diagnoses were developed using: existing diagnostic characters, the phylogenetic matrix (Appendix IV), and examination of other species from each genus.). Where phylogenetic results were informative, classification was revised to reflect phylogenetic history through synonymy, description of new genera, changes of rank, and new generic placements. Existing generic concepts were maintained where phylogenetic results were inconclusive.

## Results

Bayesian analysis of the Cardiophorinae, Negastriinae and Hypnoidini matrix resulted in trees largely agreeing with results of morphological analysis of Elateridae (Douglas 2011). They agree in finding a well-supported monophyletic Cardiophorinae (as redefined below, Fig. 36, Node d, posterior probability PP = 0.96) and Cardiophorinae + Negastriinae (Fig. 36, Node a, PP = 1.00) which together render Hypnoidini paraphyletic (Fig. 36). Among these strongly supported clades, only Cardiophorinae + Negastriinae had support above 95% after correction for branch length (branch length = 28.2, correction according to Zander 2004, table 4). As in previous analyses, the most likely sister group of this hypnoidine-cardiophorine clade was *Agriotes*. However, unlike previous analyses, the Cardiophorinae here render the Negastriinae paraphyletic. Rejection of monophyly of the Negastriinae was strong (Table 3) although Negastriinae remains largely unresolved here (Fig. 36).

**Table 3.** Tests of hypotheses of monophyly for Cardiophorinae and Negastriinae based on Bayesian posterior probabilities (Fig. 36). *Negastrius americanus* was treated as wild to increase the generality. Monophyly was considered tested if two or more taxon members were included in the analysis.

Hypothesis and citation	posterior probability
*Subfamilies*	
Cardiophorinae *auctorum*	<0.000008
Cardiophorinae (w *Negastrius americanus* and Physodactylinae spp.)	<0.000008
Negastriinae (sensu Stibick, 1979a)	0.000108
*Tribes*	
Cardiophorini Candèze, 1859 (excluding Nyctorini)	0.000267
*Genera of Cardiophorinae*	
*Aphricus* LeConte, 1853 (+/- undescribed sp. from New Zealand)	0.0017
*Aptopus* Eschscholtz, 1829	0.000033
*Blaiseus* Fleutiaux, 1931	0.9
*Cardiophorus* Eschscholtz, 1829	<0.000008
*Cardiophorus*: subgenus Cardiophorus	<0.000008
*Cardiophorus*: subgenus Perrinellus Buysson, 1899	0.065
*Cardiotarsus* Eschscholtz, 1836 (+/- *Cardiotarsus mjobergi*)	0.0036
*Esthesopus* Eschscholtz, 1829	0.016
*Horistonotus* Candèze, 1860	0.33
*Paracardiophorus* Schwarz, 1895b	<0.000008
*Phorocardius* Fleutiaux, 1931	0.00058
*Synonymies* (putative synonyms of *Cardiophorus* were not tested because its generic monophyly was rejected)	
*Dicronychus* (= *Paradicronychus* (*nomen nudum*)): Platia 1994	0.069549
*Dicronychus* (= *Platynychus*): Méquignon 1931^1^	<0.000008
*Craspedostethus* (= *Tropidiplus*): Schwarz 1906	0.000008
*Paracardiophorus* (= *Craspedostethus*): Cobos 1970a.	<0.000008

1 with or without *Cardiotarsus*, *Coptostethus*, *Phorocardius* spp. and *Paraplatynychus*.

Within the Cardiophorinae, there were several clades with moderately high support (e.g. Fig. 36, Nodes e–i), but these do not subdivide the tree into even-sized major clades. The somewhat pectinate shape of this tree makes the terms basal and apical useful here to refer to taxa nearer to or farther from the root. Resolution was low in the tree’s mid-region, especially within the paraphyletic *Cardiophorus*. Clades with more than 90% support within the Cardiophorinae include genus *Blaiseus* Fleutiaux (*Blaiseus bedeli* Fleutiaux, 1931 plus *Blaiseus nothoafricanus* Douglas, 2009, PP = 0.90), *Paracardiophorus* + *Cardiophorus cardisce* (Say, 1834) + *Cardiophorus luridipes* Candèze, 1860 (PP = 1.00, ≥ 95% after correction for branch length) and the Brazilian genera *Globothorax* Fleutiaux, 1891 + *Teslasena* (PP = 1.00, ≥ 95% after correction for branch length).

Support for *a priori* hypotheses of monophyly was mostly weak to absent (Table 3). Hypotheses of monophyly of the Negastriinae and the Hypnoidini were rejected (Table 3). Probabilities that the 11 genera tested were truly monophyletic ranged from <0.000008 to 0.90, and hypotheses of monophyly were rejected for all except *Cardiophorus*: *Perrinellus*, *Horistonotus* and *Blaiseus* (Table 3). Similarly, all tested published hypotheses of generic synonymy were found to have low support and three of four were clearly rejected

Parsimony analysis mainly corroborated results of Bayesian analysis, also with low resolution near *Cardiophorus* (Fig. 37), except where branch support was low. Unlike the Bayesian tree, *Adrastus pallens* was included in the hypnoidine-cardiophorine clade, between the Negastriinae (here monophyletic excluding *Negastrius americanus*, D =1, BS < 50%) and the Hypnoidini (monophyletic D =1, BS < 50%). The Cardiophorinae were again monophyletic (D=2, BS < 50%) in the parsimony analysis with the addition of *Negastrius americanus*, *Margogastrius*, *Pachyelater*, and *Teslasena*. In both trees *Blaiseus*, *Aphricus* and *Patriciella*, an undescribed species from New Zealand, *Pachyelater*, *Negastrius americanus*, *Nyctor*, *Neocardiophorus*, and *Margogastrius* are near the base of Cardiophorinae. The remainder of the Cardiophorinae were weakly resolved by parsimony analysis, except that as in the Bayesian analysis *Paracardiophorus* grouped with *Cardiophorus cardisce*, and *Cardiophorus luridipes*. Both analyses also included a clade of 11–13 genera (Node j of Bayesian analysis), whose genera are entirely or mainly in the southern hemisphere or northern tropical regions. These “southern clade” taxa are: *Esthesopus*; *Odontocardus*; *Triplonychoidus*; *Aptopus agrestis* Erichson, 1840; *Horistonotus*; *Paraplatynychus* Fleutiaux, 1931; *Triplonychus* Candèze, 1860; *Cardiotarsus mjobergi* (Elston, 1930); *Cardiodontulus* Van Zwaluwenburg, 1963; *Craspedostethus* Schwarz, 1898; *Paracardiophorus* species from Australia and Chile; and *Buckelater* Costa, 1973. Only parsimony analysis included genera *Globothorax* and *Teslasena* in the southern clade.

## Discussion

### Monophyly of Cardiophorinae

Bayesian (Fig. 36, Table 3) and also parsimony analyses (Fig. 37) show that the Cardiophorinae are a well-supported clade if several taxa are transferred into Cardiophorinae. Here, the Cardiophorinae can be corrected by adding a few species and genera from subfamilies Physodactylinae, Dendrometrinae and Negastriinae (*Margogastrius*, *Negastrius americanus*, *Teslasena*, and the undescribed species from New Zealand, and *Pachyelater* Lesne). Three of these also require further taxonomic alterations, as discussed below.

The resulting Bayesian tree (Fig. 36) also showed Negastriinae as paraphyletic (Nodes a–c) and strong support for monophyly of Cardiophorinae + Negastriinae (Node a). However parsimony analysis found weak support for a monophyletic core Negastriinae that is sister to the Cardiophorinae (Fig. 37). In the Bayesian analysis, support for negastriine monophyly was only 0.0001 (Table 3). The strength of this rejection is surprising, given that Bayesian analysis (Fig. 36) left Negastriinae mostly unresolved (Node a), and that support for the two nodes showing paraphyly of the Negastriinae was only 0.65 & 0.72 (Nodes b & c). Further phylogenetic analysis, including more genera of the Negastriinae, is important to further test the validity of the Negastriinae and membership of both subfamilies.

### Tribal classification of the Cardiophorinae

The existing tribal classification of the Cardiophorinae is incorrect according to both Bayesian and parsimony analyses. This is because the monotypic Nyctorini rendered the only other tribe, the Cardiophorini, paraphyletic (Table 3, Fig. 36). The only diagnostic characters of Nyctorini (Semenov-Tian-Shanskij and Pjatakova 1936), i.e., the short anterior prosternal lobe and sexual size dimorphism, are homoplastic characters found in many fossorial elaterids. For these reasons Nyctorini should be a junior synonym of Cardiophorini, effectively eliminating tribal level classification of the Cardiophorinae. Because of the generally pectinate shape of trees for Cardiophorinae (Figs 36, 37), no natural divisions were found for a convenient tribal level classification, and all Cardiophorinae should be placed in tribe Cardiophorini.

### Genera of the Cardiophorinae and Negastriinae

The only taxonomic change to Negastriinae is the transfer of *Negastrius americanus* from *Negastrius* Thomson, to Cardiophorinae (as a new genus). Within Negastriinae, three genera previously transferred from Cardiophorinae form a well-supported clade in both Bayesian (PP > 0.95 after correction for branch length, length = 27, uncorrected probability = 1.00) and parsimony (D = 5, BS = 75) analyses (Figs 36, 37). These distinctive genera, *Agrypnella*, *Cardiohypnus* and *Rivulicola* live in riparian habitats in the Neotropics, South Asia and Australia respectively. *Rivulicola* is unusual as the only Negastriinae known from Australia. These are recognizable among the Negastriinae because of their scale-like setae. All three genera were once placed in the Cardiophorinae because of their heart shaped scutella (with emarginate anterior edge), ovoid pronota and elytra, and short prosternal processes. They were transferred independently to the Negastriinae by three different authors (Dolin 1992, Golbach 1994, Calder 1996), at least in part, because of their convex prosternal sides. However, despite the similarities that these three genera share with Cardiophorinae, this group was not found sister to the Cardiophorinae here. *Fleutiauxellus* Méquignon, a negastriine appearing less like *Cardiophorus* is the most likely sister group to the Cardiophorinae according to Bayesian analysis, sharing with many Cardiophorinae the pedunculate anterior sac of the bursa copulatrix.

The required changes of classification among the Cardiophorinae are discussed beginning at the root of Cardiophorinae in the Bayesian tree. Some paraphyletic and polyphyletic genera are recognised here, in cases where phylogenetic results did not provide well-supported alternative to the prior classification. The most basal cardiophorine node is an eight-way polytomy (Fig. 36, Node d). Here, genus *Aphricus* is made paraphyletic in both analyses by at least the fossorial Australian genus *Patriciella* Van Zwaluwenburg, 1953 and an undescribed species similar to *Aphricus* from New Zealand. In order to avoid recognising a non-monophyletic genus, new genus *Chileaphricus* is established for *Aphricus chilensis* Fleutiaux. Since *Aphricus* (from California, USA) + *Patriciella* and the undescribed species from New Zealand, form a clade with moderate support (PP = 0.82, D = 1) they should be treated as a single genus (by synonymising *Patriciella* under *Aphricus*, its type species becoming *Aphricus australicus* Van Zwaluwenburg, 1947).

*Blaiseus* Fleutiaux, another basal cardiophorine, was found monophyletic here. This genus has 10 species distributed in Southeast Asia, South Africa, and Central and North America, (Douglas 2009). The type species (*Blaiseus Bedeli* Fleutiaux, 1931), and a male of the South African *Blaiseus nothoafricanus* Douglas, 2009 were included here. Although support for their monophyly was only 0.90 (also supported by parsimony, Fig. 37. Bremer support, (D) = 3, bootstrap support, (BS) = 81), the characters uniting them are distinctive. One such synapomorphy is their unique, split parameres (Fig. 25). The widespread distribution of the few known species, and the basal position of this genus within Cardiophorinae suggest *Blaiseus* is a long-separated lineage with a possibly relictual distribution.

*Pachyelater* Lesne, 1897 is a robust-bodied fossorial elaterid genus from Madagascar with sexually dimorphic males and females (Figs 50–53). Because this genus falls within the Cardiophorinae in both analyses, it should be transferred from Dendrometrinae to Cardiophorinae. Because females of *Pachyelater* are flightless and fossorially adapted the undiscovered females of the closely-related *Aphricus* may also share these traits (Fig. 36). Furthermore, females of *Aphricus* spp. may also be similarly larger than males, with reduced eyes and have the ovipositor and bursa copulatrix without sclerites. *Margogastrius* Schwarz, a genus known from only two damaged female type specimens from coastal Tanzania (also flightless and fossorial) was found with weak branch support as the sister to *Blaiseus* (although they are not closely related according to parsimony, Fig. 37). Examining internal genitalia of the remaining undissected type specimen might yield further phylogenetic information. No associated males have been identified with external morphology or distribution like these females. While the historically enigmatic species *Negastrius americanus* clearly belongs to the Cardiophorinae, the characters examined in both male and female specimens did not suggest placement in any other genus (Figs 36, 37). Therefore I propose to place it in the new monotypic genus *Floridelater* gen. n.

The remaining taxa in the polytomy of the Bayesian analysis (Fig. 36, Node d): *Neocardiophorus* Gurjeva, 1966; *Nyctor* Semenov-Tian-Shanskij & Pjatakova, 1936; and Cardiophorus subgenus Metacardiophorus Gurjeva, 1966 are all known from central Asia. Among these, only *Nyctor* is known from both sexes, so the discovery of females of the other two genera would provide important data for improved phylogenetic placement and on the evolution of flightlessness in the Cardiophorinae. Since subgenus Metacardiophorus is distantly related to subgenus Cardiophorus (Fig. 36, Node d, not h; Fig. 37), it should be raised to genus rank.

Among the genera historically confounded with *Cardiophorus*, the most basal is *Paracardiophorus*. This genus was found polyphyletic (Table 3, Fig. 36: Nodes e, f & i, Fig. 37) because it includes superficially similar species from Australia and Chile. It is argued below that those should be part of a new genus. The type species of *Paracardiophorus* forms a fully supported clade with two North American *Cardiophorus* species (PP > 0.95 after correction for branch length (12), uncorrected probability =1.00), which is also indicated by parsimony analysis (Fig. 27, D = 2, BS = 57). These and all other North American species with the same apparent synapomorphies should be transferred to *Paracardiophorus*. These are the North American *Cardiophorus* with the base of the female spermathecal gland duct sclerotised (Figs 34, 35), some species also have truncate pronotal hind angles (Fig. 4), and the aedeagal parameres spatulate (Fig. 65). *Paracardiophorus* is the most likely (PP = 0.67) sister group of the remainder of Cardiophorinae.

Beyond confusion with *Paracardiophorus*, genus *Cardiophorus*: subgenus Cardiophorus was paraphyletic at four nodes (Fig. 36, f–i). It is also paraphyletic at 2 or more nodes in parsimony analysis, with most forming a polytomy in the parsimony analysis (Fig. 37). This large genus, which contains half the described cardiophorine species, is paraphyletic because it also includes 21 other genera (Fig. 36, Node h). Unfortunately, because of this poor phylogenetic resolution, there is little basis yet for an improved definition of *Cardiophorus*. The monotypic *Cardiophorus*: subgenus Zygocardiophorus Iablokoff-Khnzorian & Mardjanian, 1981 was found to be sister to *Cardiophorus* + the remainder of Cardiophorinae (Nodes f, g) and thus should be raised to genus rank. The position of *Cardiophorus*: subgenus Lasiocerus Buysson is unknown, because no specimens were available for examination.

Dolin and Gurjeva (1975) described genus *Paradicronychus* based on larval characters only (although conspecific adults were also known), and without a formal designation of a type species. Because of IZCN regulations for genera described after 1930 (Art. 13.3), *Paradicronychus* is not an available name. Although larvae of many cardiophorines from the former USSR are known, larval morphology of the world fauna remains too poorly documented to define genera based on larvae alone. Both analyses placed *Cardiophorus inflatus* Candèze, 1882, (considered *Paradicronychus* by Dolin and Gurjeva (1975)) within the broadly paraphyletic *Cardiophorus*. Because of this result, and because no adult characters were identified to distinguish it from *Cardiophorus*, the *nomen nudum* name *Paradicronychus* should be placed as synonym of the nominate subgenus of *Cardiophorus* (with its included species to *Cardiophorus* as *Cardiophorus inflatus* Candèze, 1882, and *Cardiophorus nothus* Candèze, 1865).

Two other *Cardiophorus* subgenera, *Coptostethus* Wollaston and *Perrinellus* Buysson are each based on a single evolutionarily labile character (reduction of flight wings, and narrowed base of scutellum respectively), and are probably not monophyletic (although not synonymised here). The first, *Coptostethus*, is a name historically applied to various short-winged Cardiophorinae. Some *Cardiophorus* from Africa and Eurasia possibly adapted for fossorial life have been grouped into the subgenus Perrinellus, which was not recovered as monophyletic in either analysis (PP = 0.06, Table 3). Evidence that numerous other cardiophorines have similar modifications for digging may be further evidence these characters are convergent and this assemblage is artificial. While these genera remain non-monophyletic and weakly defined, I do not recommend taxonomic changes until their positions are better resolved.

*Globothorax* Fleutiaux and *Teslasena* Fleutiaux (Physodactylinae) are a strongly supported (Fig. 36. Uncorrected PP = 1.00, branch length = 2; Fig. 37, Bremer support = 2) clade within *Cardiophorus* and the other genera rendering it paraphyletic (Node g). Like the other genera here, these two should not be synonymised under *Cardiophorus*. *Teslasena* should be considered a junior synonym of *Globothorax*, because of the well-supported monophyly of these two species. This synonymy means that included species *Teslasena femoralis* (Lucas, 1857), *Teslasena foucarti* Chassain, 2005, and *Teslasena lucasi* Fleutiaux, 1899 are transferred to *Globothorax* as *Globothorax femoralis* (Lucas, 1857, *Anelastes*); *Globothorax foucarti* Chassain, 2005; and *Globothorax lucasi* Fleutiaux, 1899 respectively. Their sympatry in Brazil further supports the hypothesis that the known specimens of *Teslasena* and *Globothorax* are dimorphic males and females of one genus (although not necessarily conspecific). I recommend this despite characters presented by Rosa (2014) distinguishing the two genera: these may be variation between species, or sexual dimorphism but their presence does not refute the hypothesis that they are best understood as congeneric.

*Dicronychus* Brullé was coded here based on *Dicronychus cinereus* Brullé, which was considered the senior synonym of the type species at the beginning of this study. Although within the Paraphyletic *Cardiophorus* according to both analyses (Figs 36, 37), *Dicronychus* should not be a synonym of *Cardiophorus*, at least until a monophyletic *Cardiophorus* can be defined. However, since *Dicronychus* is only distinguished from *Cardiophorus* by the presence of a second tarsal claw tooth (Fig. 21), there may be no basis on which to distinguish it from *Cardiophorus* even at the species level because of apparent intraspecific dimorphism. Such dimorphic claws may underlie the sympatric *Cardiophorus aptopoides* Candèze, 1865; and *Cardiophorus brevis* (Candèze, 1859) from Mexico, which appear identical except the presence or absence of a basal claw tooth (including aedeagal shape and regional colour variants). A similar otherwise apparently identical *Cardiophorus*-*Dicronychus* pair of species (*Cardiophorus varius*, Cate et al., 2002 and *Dicronychus hoberlandti* Cate et al., 2002) from Iran also may be a single species with dimorphic claws. There is no evidence for the monophyly of genera *Dicronychus* and *Platynychus* Motschulsky, 1858 (PP = 0.07, Figs 36, 37, Table 3), therefore *Platynychus* should be removed from synonymy under *Dicronychus*, where it has been placed by some authors. *Platynychus* is distinguished from both *Cardiophorus* and *Dicronychus* by its closed procoxal cavities.

Genus *Cardiophorellus* Cobos also falls within the paraphyletic nominate subgenus of *Cardiophorus* (Fig. 36, Node h, not contracted by parsimony, Fig. 37). The type species of *Cardiophorellus* is much like *Cardiophorus* except the anterior edge of its scutellum is broadly concave and not angulately emarginate, and its mandibles are simple. Due to phylogenetic uncertainty, there is no evident best taxonomic placement for *Cardiophorellus*. Because of this uncertainty, and because *Cardiophorellus* is readily diagnosed, it seems best to continue to consider *Cardiophorellus* a valid genus. The type specimen of the monotypic subgenus Cardiophorellus (Parapleonomus) Cobos, 1970 was not found at MNHN (Paris), so I cannot comment on its validity or rank.

Although the hypothesis of *Aptopus* Eschscholtz monophyly was rejected (Table 3, Fig. 36 (Nodes h & k), Fig. 37), this only affects the placement of the species *Aptopus agrestis* (Erichson). Apart from its pectinate claws, this species is like *Horistonotus* species with costate elytral intervals. Because parsimony phylogenetic analysis suggested *Aptopus agrestis* was the most likely sister taxon to *Horistonotus simplex* LeConte, such *Aptopus* species with carinae following the lateral edge of the pronotum should be transferred to *Horistonotus*. However, because the type specimen of *Aptopus agrestis* was not examined, this species is not transferred to *Horistonotus* here. The concept of *Aptopus* used here is from modern authors (e.g. Aranda 1998, also Section 1 of Candèze 1860) because the type specimens of the type species, *Aptopus tibialis* Eschscholtz, 1829 are lost or were unavailable for examination and because the only published species description lacks detail (eight words only).

Genus *Phorocardius* Fleutiaux was described to include *Cardiophorus*-like species with apically bidentate tarsal claws (Fig. 22, not Fig. 21), however the nominate subgenus + subgenus Diocarphus Fleutiaux are not monophyletic (Table 3). Therefore *Phorocardius* and *Diocarphus* should be recognized as distinct genera despite uncertainty about their positions in the poorly resolved nodes near *Cardiophorus* (Figs 36, 37). *Tropidiplus* Fleutiaux, 1903 is a distinctive east African genus among the genera rendering *Cardiophorus* paraphyletic. The hypothesis (Schwarz 1906) that *Tropidiplus* is a synonym of *Craspedostethus* was clearly rejected (Table 3, Fig. 36, also Fig. 37). Similarly *Displatynychus* Ôhira was a subgenus of *Platynychus* until Ôhira (1987) raised it to genus rank. Bayesian analysis (Fig. 36) supports separation of *Displatynychus* from *Platynychus* (not contradicted by parsimony, Fig. 37).

Genus *Cardiotarsus* includes species from Africa, Mauritius, S. and E. Asia and Australia. These analyses included the type species (*Cardiotarsus capensis* Candèze, 1860, known here from females only), another (undescribed) African species and *Cardiotarsus mjobergi*, Australia’s only known species. Bayesian hypothesis testing (Table 2) rejected the hypothesis that even the two African species were monophyletic (also not recovered by parsimony, Fig. 37). *Cardiotarsus mjobergi* was placed at Node k of the Bayesian tree (Fig. 36) within the southern clade (Fig. 36 node j). I propose transfer of *Cardiotarsus mjobergi* to genus *Cardiodontulus*, from Papua New Guinea, because of this non-monophyly and it matches the Van Zwaluwenburg’s definition of that genus. This placement is also plausible, because both are from the Australian biogeographic region. Although the type specimen of *Cardiotarsus mjobergi* was not examined, I am confident in the identification of the specimens examined because they were from near the type locality, which is in a well-collected area near a major insect collection, and this species was also illustrated in Calder’s (1996) guide to Australian Elateridae. Otherwise, I propose no changes to the biologically inaccurate (but easily diagnosable) genus *Cardiotarsus* until the phylogeny of Cardiophorinae is better resolved.

The remaining apical southern clade (PP = 0.81, Fig. 36: Node j) is composed mainly of Australian and Neotropical species, plus two South Asian genera and one from Africa. This clade was also inferred by parsimony (Fig. 37, but with *Globothorax* and *Teslasena* added), and includes mostly species with bilobed or multilobed proximal sclerites of the bursa copulatrix, and many of the species with closed procoxal cavities, and lacking lateral expansions of the parameres. Among these, the monophyly of each of *Odontocardus* Fleutiaux, 1931; *Triplonychoidus* Schwarz, 1906; *Paraplatynychus* Fleutiaux, 1931; *Triplonychus* Candèze, 1860; *Cardiodontulus* Van Zwaluwenburg, 1963; *Craspedostethus*; and *Buckelater* were not tested. These genera remain unaltered, except as discussed for *Cardiodontulus*. Two large, mainly Neotropical genera (extending into temperate North America) *Esthesopus* and *Horistonotus* are both not monophyletic (Table 3). However their definitions and status should be maintained until better resolution is available. The definition of *Horistonotus* is broadened here to include species with multiple claw points.

Of the five species in the weakly supported apical clade (Fig. 36, Node l), two belong to the polyphyletic genus *Paracardiophorus*. These two species from Australia and Chile are rendered paraphyletic (also at low posterior probability) by *Buckelater*, from Brazil. Because the included Australian and South American *Paracardiophorus* are identical in most characters including the male and female genitalia, I propose placement of them in a new genus along with other species from both continents sharing their diagnostic characters. The type species of this new genus, *Austrocardiophorus*, is *Cardiophorus humeralis* Fairmaire & Germain, 1860 from Chile (recently in *Paracardiophorus*). This solution is considered preferable to placement in the currently monotypic *Buckelater* because its female genitalic characters remain unknown, which contributes to taxonomic uncertainty.

### Character evolution

This section outlines some character state changes implied by the trees (Figs 36, 37), which may be diagnostically helpful. While these characters may be true synapomorphies of their groups, Bayesian analysis does not rely on identifying them unambiguously as such.

Three characters unite the Negastriinae + Cardiophorinae. The fusion of the parameres at their midlength into a tube (Char. 117, Figs 24, 25) appears unique among the Coleoptera (Iablokoff-Khnzorian and Mardjanian 1981), and universal among Cardiophorinae and Negastriinae. Examination of two other possible synapomorphies revealed more intrageneric variability than found by Douglas (2011). Firstly, the hind-wing membrane has an anal notch (Fig. 17, at AA4) in all examined Negastriinae except *Migiwa* Kishii, 1966, but this notch is present in only most Cardiophorinae (Char. 75). Secondly, all included Negastriinae, except *Arhaphes* Candèze, 1860, but only most cardiophorine genera had a tridentate lobe at the midline of the posterior edge of the pronotum. The only character to distinguish the Cardiophorinae from the Negastriinae, was an apparent reversal to straight-sided prosternum (alternative = convex, Char. 36). No variation from this character-state was found in Cardiophorinae.

No clear evidence was found for basal synapomorphies of Negastriinae not also shared by Cardiophorinae. As found by Douglas (2011), they were distinguished from Cardiophorinae by their convex lateral edges of the prosternum (near midlength). However, this character is an apparent symplesiomorphy shared with *Hypnoidus* and *Tropihypnus* according to the most likely topologies identified by Douglas (2011).

Several synapomorphies unite three brightly patterned riparian negastriine genera from the Neotropics (*Agrypnella*), the Himalayan foothills (*Cardiohypnus*), and Australia (*Rivulicola*). These are the only Negastriinae with sublateral pronotal incisions and carinae (Char. 29). They are also the only Negastriinae, except for *Monadicus*, with: scale-like setae (Char. 23); and the posterior edges of hypomeron mesad of hind angles with rectangular or semicircular indentations (Char. 32). Two of these, *Agrypnella* and *Cardiohypnus*, also have sides of pronotum overhanging the lateral carinae like in *Cardiophorus*.

*Quasimus* Gozis, 1886 + *Yukoana* Kishii 1959 (both Negastriinae, Quasimusini) share several possible synapomorphies: tarsomere 4 (and no others) is lobed on all legs (shared in Negastriinae by only *Neoarhaphes* Costa 1966, Char. 94); pronotal hind angles with dorsal angle carina reaching anterior edge of pronotum (shared in Negastriinae with *Monadicus* and *Agrypnella*, Char. 27); parameres with two setae each (shared in Negastriinae with *Arhaphes*, *Cardiohypnus*, and *Agrypnella*, Char. 123); and pronotosternal sutures with anterior ends grooved (shared in Negastriinae with *Monadicus* Candèze, 1860, and *Zorochros* Thomson, 1859, Char. 37). *Arhaphes* + *Neoarhaphes* share two unique characters: bottle-shaped (lageniform) apical segments of the labial and maxillary palpi (Char. 19); partially or completely fused prosternum and pronotum (Char. 38); and also a tubercle at the posterior end of the mesosternal cavity (shared in Negastriinae with *Migiwa* only, Char. 52).

The Cardiophorinae have only two apparent synapomorphies not shared with at least some Negastriinae: the straight-sided prosternum (Char. 36); and presence of paired proximal sclerites in the bursa copulatrix (absent in *Blaiseus*, *Craspedostethus*, *Floridelater* (formerly *Negastrius americanus*), and *Pachyelater*, Char. 152). A third possible synapomorphy, the presence of one or two pedunculate anterior sacs of the bursa copulatrix (Char. 143) is shared by all examined Cardiophorinae and their apparent sister-taxon, *Fleutiauxellus*.

Within Cardiophorinae, only a few groups were united by moderate to high branch support. *Pachyelater* + *Aphricus* + undescribed species from New Zealand + *Patriciella* share straight sides of the mesosternal cavity posterior to anterior edge of mesocoxae (Char. 52). The Palaearctic *Paracardiophorus* + the Nearctic *Cardiophorus cardisce* + *Cardiophorus luridipes* all share dorsally truncate pronotal hind angles.

## Future research

Additional phylogenetic research with more taxon sampling is needed throughout Cardiophorinae to test generic monophyly and better understand intergeneric relationships. Additional collecting and taxon sampling would be useful among the basal cardiophorines, for which only two of nine genera are known from both sexes.

Some areas of the tree have low clade support and short branch lengths. These may approximate a hard polytomy, and thus it might be impossible to infer branching patterns using morphology alone. Combined analysis of multiple gene regions plus morphology might resolve these regions, once specimens suitable for DNA sequencing have been collected. Discovery of undescribed females or males from several genera would also provide useful data. Meanwhile I recommend continuing to recognize some heterogeneous genera until phylogenetic knowledge improves.

## Key to genera of extant Cardiophorinae, based on adults

### Key does not include *Cardiophorellus*: subgenus Parapleonomus Cobos 1970

**Table:** 

1	Prosternum with sides near midlength convex, or partly fused with pronotum; if scutellum emarginate anteromesally, then with dorsal vestiture of scale-like or apically broadened setae; some with tubercles between setal punctures on pronotum; bursa copulatrix with symmetrical pair of spine-bearing sclerites absent, or connected to each other by semi-sclerotised tissue	Not Cardiophorinae: Negastriinae (revised by Stibick (1971), with subsequent changes by Calder 1996, Dolin 1976, Dolin 1992, Dolin and Girard 1998, Golbach 1994, Kishii 1976)
–	Prosternum with sides near midlength straight or concave, not fused with pronotum. Most with scutellum emarginate anteromesally; setae evenly tapered in all; none with tubercles between pronotal setae; most with minute serrations along upper edge of elytral epipleurae and/or at sides of urosternites 3–7; bursa copulatrix of most with symmetrical pair of separate, spine-bearing sclerites (proximal sclerites); Cardiophorinae	**2**
2 (1)	Pronotum with complete carina at lateral edge or on hypomeron, reaching from hind angle to anterior edge (reaches only 9/10 to anterior edge in two Southeast Asian *Paraplatynychus* species). From Oriental and Ethiopian realms)	**3**
–	Pronotum with lateral carina not reaching anterior edge (ventrad of lateral edge of pronotum in some, called submarginal line in earlier publications), [rest of Cardiophorinae]	**4**
3 (2)	Tarsal claws with or without basal point; tarsal claws with basal setae (Fig. 106, possibly absent in some); bursa copulatrix with proximal sclerites ovoid (Fig. 28). In type species (*Tropidiplus tellinii*), urosternite 7 has longitudinal grooves and second longitudinal carina near lateral edge (Eritrea, Ethiopia, Mozambique, Kenya)	***Tropidiplus* Fleutiaux, 1903**
–	Tarsal claws with both basal and apical points, without basal setae; bursa copulatrix with proximal sclerites bilobed (Fig. 142), (Southeast Asia)	***Paraplatynychus* Fleutiaux, 1931**
4 (2)	Pronotum with lateral carina present (short in some) but below edge of dorsal part of pronotum (Fig. 3); proximal (largest) sclerites of bursa copulatrix ovoid (Figs 28, 66, 72, 90, 95, 103, 113, 118), unispinose (Fig. 154) or absent, not rigid with membranous extensions	**5**
–	Pronotum with lateral carina extending anterad from hind angles following lateral edge (Fig. 149) or completely absent; proximal (largest) sclerites of bursa copulatrix ovoid (Figs 28), bilobed (Figs 29, 156, 162), multilobed (Fig. 31), parallel sided (Fig. 30), partially membranous (Figs 138, 162), or absent	**20**
5 (4)	Tarsomere 4 with ventral lobe or pad extending beyond base of tarsomere 5 (Fig. 128)	**6**
–	Tarsomere 4 without ventral lobe or pad extending beyond base of tarsomere 5	**7**
6 (5)	Tarsal claws one apex per side (Africa, Mauritius, S. and E. Asia, Japan, Taiwan)	***Cardiotarsus*Eschscholtz 1836**, part (type species, not monophyletic).
–	Tarsal claws with both basal and apical points (Figs 21, 22, Cambodia, Vietnam, Laos, Philippines)	***Odontocardus* Fleutiaux, 1931**
7 (5)	Tarsal claws with 3 or more points per side (as in Fig. 134, SW North America to Argentina)	***Aptopus* Eschscholtz, 1829**
–	Tarsal claws with single apical point or both basal and apical points on each side	**8**
8 (7)	Tarsal claws with 2 points per side (Figs 21, 22)	**9**
–	Tarsal claws with only 1 point per side	**14**
9 (8)	Procoxal cavities open	**10**
–	Procoxal cavities closed	**11**
10 (9)	Tarsal claws with second point near apex on each side (Fig. 22); hind wing not notched in anal area (S. and S.E. Asia)	***Phorocardius* Fleutiaux, 1931**
–	Tarsal claws with second point at base of each side (Fig. 21); hind wing notched in anal area (Eurasia, Africa)	***Dicronychus* Brullé, 1832**. Some brachypterous spp. of *Cardiophorus* s.g. *Coptostethus* key to here
11 (9)	Head with area between antennal fossa and compound eye unsculptured; bursa copulatrix with paired distal sclerites (pair farthest from vagina) present and fused into a “U” shape (Fig. 33); base of spermathecal gland duct inside bursa without tube-like sclerotisation (Eurasia)	***Platynychus* Motschulsky, 1858** (monophyly unknown)
–	Head with area between antennal fossa and compound eye with carina connecting fossa and eye, or with 2 pits with non-depressed area between them or with a single pit; bursa copulatrix without distal sclerites (*i.e.* a second pair, farther from vagina, at base of spermathecal gland duct); base of spermathecal gland duct with tube-like sclerotisation (Figs 111, 113)	**12**
12 (11)	Head with area between antennal fossa and compound eye with carina connecting fossa and eye, or with 2 pits with non-depressed area between them; tarsal claws with ventral surface convex mesad of basal apex (as in Fig. 21) (Japan)	***Displatynychus* Ôhira, 1987**
–	Head with area between antennal fossa and eye with a single pit; tarsal claws with ventral surface concave mesad of basal apex (Fig. 22) (Vietnam)	***Diocarphus* Fleutiaux, 1947**
14 (8)	Mandibular apex unidentate (simple)	**15**
–	Mandibular apex bidentate or tridentate	**17**
15 (14)	Head with supra-orbital groove absent; posterior edges of hypomeron mesad of hind angles without indentations (Uzbekistan, only males known)	***Metacardiophorus* Gurjeva, 1966**
–	Head with supra-orbital groove (Fig. 2); posterior edges of hypomeron mesad of hind angles with rectangular (Fig. 3) or semicircular indentations	**16**
16 (15)	Scutellum with anterior edge broadly concave (Fig. 7); prosternum with anterior edge short, exposing labium; tibiae with posterior surfaces convex, only weakly modified for digging (South Africa, only males known)	***Cardiophorellus* Cobos, 1970** (3 spp., Congo). Subgenus Parapleonomus not examined here.
–	Scutellum with anterior edge abruptly emarginate (Fig. 6); prosternum with anterior edge not short, produced as lobe, concealing labium; tibiae with posterior surfaces flattened and broadened apically (Fig. 42), apparently strongly modified for digging (Tanzania, only females known)	***Margogastrius* Schwarz, 1903** (monotypic, *Margogastrius schneideri* Schwarz)
17 (14)	Edge of elytra in dorsal view between anterior-most point and humeral angle arcuate or straight, without sinuation (Fig. 16); bursa copulatrix containing a tube-like sclerotisation of base of spermathecal gland duct (Figs 34, 35)	**18**
–	Edge of elytra in dorsal view between anterior-most point and humeral angle sinuate or tuberculate (Fig. 15); base of spermathecal gland duct not sclerotised	**19**
18 (17)	Head with supra antennal carina not elevated, with area between carina and base of labrum not concave in lateral view, carina not forked beside compound eye (Fig. 1). Elytra all-black, with or without apical shelf-like apical extensions (Fig. 71); sclerotisation of base of spermathecal gland duct without paired plate-like appendages (S.W. Asia)	***Zygocardiophorus* Iablokoff-Khnzorian & Mardjanian, 1981, stat. n**. (monotypic, Z. *nigratissimus* (Buysson 1891)
–	Head with supra antennal carina elevated, with area between carina and base of labrum concave in lateral view, carina forked beside compound eye (Fig. 2). Elytron without apical shelf-like extension; elytra with or without pale spots; bursa copulatrix with tube-like basal sclerotisation of spermathecal gland duct (Figs 34, 35), some also L-shaped or with paired plate-like (Fig. 34) appendages (Holarctic)	***Paracardiophorus* Schwarz, 1895**, part
19 (17)	Pronotum with dorsal hind angle carinae extending to anterior quarter; dorsal surface of labrum flat in side view; bursa copulatrix with a pair of flexible concave sclerites (Fig. 154), or none (Cameroon to Iran)	***Craspedostethus* Schwarz, 1898**
–	Pronotum with dorsal hind angle carina not reaching anterior third (Fig. 3); labrum convex in side view; bursa copulatrix with spiny ovoid proximal sclerites (Fig. 83, right), most also with separate or fused distal sclerites (a second pair next to spermathecal gland duct, fig. 83, left), (North America, Eurasia, Africa)	***Cardiophorus* Eschscholtz, 1829** (paraphyletic)
20 (4)	Tarsomere 4 lobed or lamellate (Fig. 128), apex of tarsomere 4 reaches under base of tarsomere 5	**21**
–	No tarsomeres lobed or lamellate, apex of tarsomere 4 vertical	**26**
21 (20)	Tarsal claws with 2 points per side (Fig. 21)	**22**
–	Tarsal claws with 1 point per side	**24**
22 (21)	Scutellum with middle of anterior edge straight (Fig. 6), (South and North America)	***Esthesopus* Eschscholtz, 1829**
–	Scutellum with middle of anterior edge concave: broadly or emarginate (Figs 7, 8, 9)	**23**
23 (22)	Elytra with apical half of interval 9 flattened or rounded (Australia, Papua New Guinea)	***Cardiodontulus* Van Zwaluwenburg, 1963**
–	Apical half of elytral interval 9 costate	***Triplonychoidus*Schwarz 1906** (Mexico to South America)
24 (21)	Mandibles with apices simple and aedeagus with free portion of parameres split vertically into dorsal and ventral lobes (Fig. 25). Male abdominal segment 9 with tergite and sternite articulated at base. Most species with tibiae broadened (Fig. 49), apparently for digging (PR China to Malaysia, South Africa, Mexico to Honduras)	***Blaiseus* Fleutiaux, 1931**, part
–	Mandibles with 2–3 points; tergite and sternite of male abdominal segment 9 articulated at sides; aedeagus with parameres not split (Fig. 157); tibiae not modified for digging	**25**
25 (24)	Distance between antennae only equal to ¼ width of head (across of compound eyes), nasale facing ventrally; procoxal cavities closed; hind wing notched in anal area (only males known, Brazil)	***Buckelater*Costa 1973** (monotypic, *Buckelater argutus* Costa 1973)
–	Distance between antennae more than ¼ head width; nasale facing anteroventrally; procoxal cavities open; hind wing not notched in anal area (South Africa)	***Cardiotarsus*** part
26 (20)	Tarsal claws with multiple points per side (including basal tooth); bursa copulatrix with 1 pair of sclerites	**27**
–	Tarsal claws with one apex per side; bursa copulatrix with 0–4 sclerites	**31**
27 (26)	Tibiae flattened and broadened apically (Figs 78, 80), apparently modified for digging; elytral intervals 1–8 rounded; sclerites of bursa copulatrix parallel sided (Fig. 30). Females with compound eyes and antennae reduced (Bolivia, Brazil)	***Globothorax* Fleutiaux, 1891**
–	Tibiae not modified for digging; some with apical half of elytral intervals 1–9 costate (Fig. 145); bursa copulatrix with proximal sclerites ovoid or parallel sided (Figs 28, 30)	**28**
28 (27)	Tarsal claws with only 2 points per side; bursa with proximal sclerites ovoid or bilobed	**29**
–	Tarsal claws with more than two points per side; known females with bursa with proximal sclerites elongate, parallel sided	**30**
29 (28)	Bursa copulatrix with proximal sclerites not bilobed (Fig. 138), (USA to Argentina, not monophyletic)	***Horistonotus* Candèze, 1860**, part (type species keys here)
–	Bursa copulatrix with proximal sclerites bilobed (Fig. 162), (Japan and Taiwan)	***Ryukyucardiophorus* Ôhira, 1973**
30 (28)	Tarsal claws with 3 points per side; elytra with apical half of intervals 1–8 costate (Fig. 145); head with area between antenna fossa and compound eye with either carina connecting fossa and eye, or with 2 pits with non-depressed area between; urosternite 7 with second carina mesad of lateral carina (South and Central America)	***Triplonychus* Candèze, 1860**
–	Tarsal claws with 7 or more points per side (Fig. 134); head with area between antenna fossa and edge of compound eye with a single pit; elytra with apical half of intervals 1–8 rounded; urosternite 7 without second carina mesad of lateral carina (Brazil)	***Aptopus agrestis*** (Erichson, 1840, within expanded concept of *Horistonotus*)
31 (26)	Scutellum with middle of anterior edge convex (Fig. 6) to broadly concave (Figs 7, 9)	**32**
–	Scutellum with middle of anterior edge emarginate (Fig. 8)	**36**
32 (31)	Procoxal cavities closed; mesepisternum with projection of anteromesal corners concave mesally; anterior edge of elytra in dorsal view between anterior-most point and humeral angle sinuate or tuberculate (Fig. 15); bursa copulatrix with bilobed sclerites (Fig. 156), (Chile and Australia)	***Austrocardiophorus* gen. n.**
–	Procoxal cavities open; mesepisternum with anteromesal corners rounded; anterior edge of elytra evenly rounded (Fig. 16), straight, or sinuate in dorsal view; bursa copulatrix of known females without sclerites	**33**
33 (32)	Scutellum with posterior apex bilobed (Fig. 9); prosternum with anterior edge produced as lobe, concealing labium when head not extended; pronotum with setae on disc on tubercles; hind wing with veins weakly sclerotised, wing area less than half of elytral area (USA, Gulf of Mexico shore)	***Floridelater* gen. n.**
–	Scutellum with posterior apex not bilobed; prosternum with anterior edge short, exposing labium; pronotum with setae on disc not on tubercles; hind wing with venation well developed, some or all with wing area greater than elytral area	**34**
34 (33)	Aedeagus with paramere apices forked (Fig. 25) (PR China to Malaysia, South Africa, Mexico to Honduras)	***Blaiseus* Fleutiaux, 1931** (part)
–	Aedeagus with paramere apices not forked	**35**
35 (34)	Labrum flat in lateral view; tibiae modified for digging, or not (Fig. 55)	***Aphricus* LeConte, 1853** (part)
–	Labrum convex dorsally in lateral view; tibiae modified for digging (Figs 50, 51), posterior surface flattened and broadened apically	***Pachyelater* Lesne, 1897**
36 (31)	Prosternum with anterior edge short, exposing labium	**37**
–	Prosternum with anterior edge not short, produced as lobe, concealing labium when head not extended	**39**
37 (36)	Head with area between antenna fossa and edge of compound eye unsculptured; hind wing notched in anal area (between AA3+4 and AP); parameres with 2 setae each (central Asia)	***Nyctor expallidus* Semenov-Tian-Shanskij & Pjatakova, 1936**
–	Head with area between antenna fossa and compound eye with carina connecting fossa and eye or with 2 pits with non-depressed area between; hind wing notched or not in anal area; parameres of aedeagus each with 3 (Fig. 41) or more setae	**38**
38 (37)	Labrum flat in lateral view; frons without supra-orbital groove; prosternum with anterior edge notched at midline; mesocoxal cavity closed to mesepimeron and mesepisternum by extension of mesosternum; mesotrochantin hidden ….	***Aphricus* LeConte, 1853** (part)
–	Labrum convex in side view; frons with supra-orbital groove; prosternum with anterior edge at midline arcuate; mesocoxal cavity open to mesepimeron and mesepisternum (Figs 11, 39), mesotrochantin exposed (Chile)	***Chileaphricus* gen. n.**
39 (36)	Mandibles with apices simple, scutellum pointed at posterior apex; urosternites 3–7 with or without serrations along sides (Uzbekistan, monotypic, only males known)	***Metacardiophorus* Gurjeva, 1966, stat. n**. (monotypic, *Metacardiophorus sogdianus* Gurjeva)
–	Mandibles with 2 points, scutellum pointed or rounded at posterior apex; urosternites 3–7 without serrations along sides	**40**
40 (39)	Pronotum with carina along lateral edge reaching more than halfway to anterior edge; bursa copulatrix with paired proximal sclerites partially membranous between spines (Ryukyu Islands, Japan)	***Allocardiophorus* Ôhira, 1989**
–	Pronotum with carina along lateral edge restricted to hind angles, or reaching less than halfway to anterior edge; female bursa copulatrix with proximal sclerites solid (Fig. 72) or unknown, but probably not semi-membranous between spines	**41**
41 (40)	Scutellum with anterolateral edges evenly rounded (Fig. 8)	**42**
–	Scutellum with anterolateral edges straight or concave posterior to anterolateral corners (Fig. 6)	**43**
42 (41)	Scutellum with posterior apex evenly rounded (central Asia, only males known)	***Neocardiophorus* Gurjeva, 1966**
–	Scutellum with posterior apex pointed; wings incapable of flight (cave inhabiting species, Porto Santo Island, Madeira Archipelago)	**Cardiophorus (Coptostethus) Wollaston, 1854** (part). Some *Cardiophorus*: *Coptostethus* spp. from the Canary Islands have tarsal claws with 2 apices per side.
43 (41)	Supra-antennal carina without longitudinal split next to eyes (Fig. 1); pronotum with hind angles not truncate dorsally (Fig. 3); posterior edges of hypomeron mesad of hind angles with rectangular or semicircular indentations (Fig. 3); procoxal cavities open; scutellum narrowed anterad; parameres approximately cylindrical (Fig. 99) near apex (Israel)	**Cardiophorus (Perrinellus) argentatus Buysson, 1899** (other spp from N. Africa, Ceylon, Central Asia, probably not monophyletic with this). Cardiophorus (Lasiocerus) du Buysson, described from Azerbaijan for a species with long antennae with dense setae, and later synonymised under s.g. *Perrinellus* was not located for examination, and may not match these key characteristics.
–	Supra-antennal carina with longitudinal split next to eyes (Fig. 2); most species with pronotal hind angles truncate dorsally (Fig. 4) so apex is composed of only the narrow hypomeral portion; most species with posterior edges of hypomeron mesad of hind angles straight or sinuate; procoxal cavities open or closed; free portion of parameres cylindrical to flattened (Fig. 65); bursa copulatrix with proximal sclerites ovoid (Figs 28, 66), distal sclerites absent; base of spermathecal gland duct sclerotised (Figs 34, 35) inside bursa copulatrix (Holarctic)	***Paracardiophorus* Schwarz, 1895** (part)

## Descriptions of genera and species

### Descriptions of new and redefined genera.

#### Austrocardiophorus gen. n.

Taxon classificationAnimaliaColeopteraElateridae

http://zoobank.org/BA79A019-6951-49E7-B6BB-CA7BA868051E

Figs 155–157

##### Type species.

*Cardiophorus humeralis* Fairmaire & Germain, 1860

##### Diagnosis.

Prothorax. Pronotum with carina along lateral edge visible in dorsal view, not reaching anterior edge; procoxal cavities closed. Pterothorax. Scutellum with anterior edge broadly concave. Legs. Tarsi non-lobed and tarsal claws with one apex per side.

##### Description.

**Figures 1–13.**
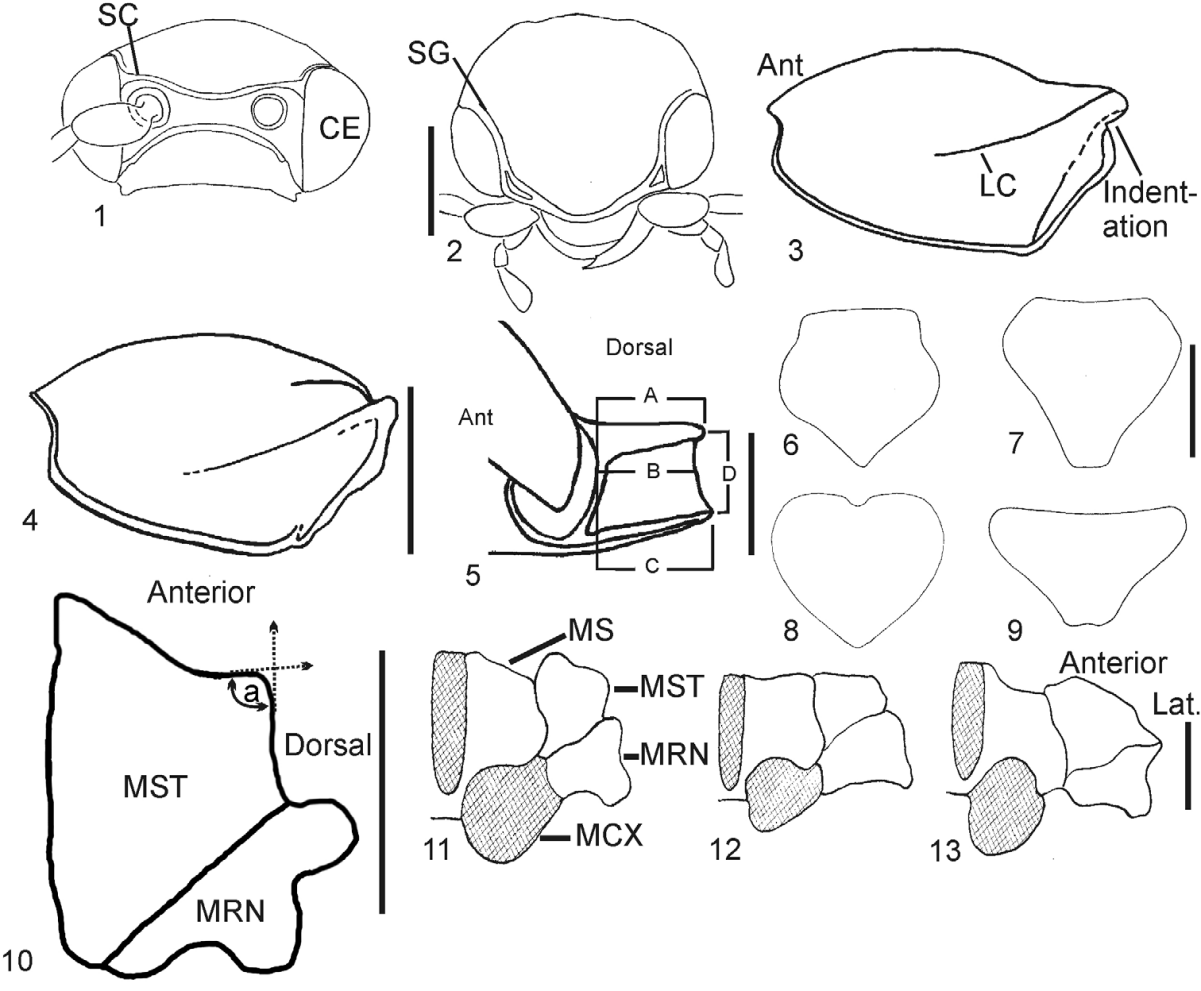
**1–2** Frontoclypeal area *Cardiophorus* Scale bar = 0.5 mm). **1** *Cardiophorus convexus* (Say), (anteroventral view) **2** *Cardiophorus gramineus*, (dorsal view). CE = compound eye; SC = supra-antennal carina; SG = supra-orbital groove. Figures captions include post-revision names **3–4** Latero-ventral view of hypomeron Scale bar = 1 mm) **3** *Cardiophorus gagates* Erichson **4** *Paracardiophorus propinquus* Lanchester. Ant = anterior; LC = lateral carina **5** Lateral view of prosternal process of *Cardiophorus erythropus* Erichson Scale bar = 0.5 mm). **A** length from procoxa to dorsal apex; Ant = Anterior **B** length from procoxa to end at halfway between dorsal and ventral apices **C** length from procoxa to ventral apex **D** vertical distance between dorsal and ventral apices. Dorsal and ventral apices are considered points where profile of respective surface is 45º from horizontal **6–9** Dorsal view of scutellum of Cardiophorinae Scale bar = 0.5 mm) **6** *Esthesopus castaneus* **7** *Blaiseus bedeli* **8** *Cardiophorus gramineus* **9** *Floridelater americanus* **10** Latero-ventral view of mesepimeron and mesepisternum of *Cardiophorus fenestratus* (LeConte) showing measurement of angle (a) of anterolateral corner of mesepisternum Scale bar = 0.5 mm). MST = mesepisternum; MRN = mesepimeron **11–13** Ventral view of left side of mesocoxal cavity of Elateridae (After Arnett 1960, scale bar = 1 mm) **11** Elaterinae sp. **12** *Hypnoidus* sp. **13** Cardiophorinae sp. Lat = lateral; MCX = mesocoxae MRN = mesepimeron; MST = mesepisternum; MS = mesosternum. Captions reflect the revised classification.

Length 3–10 mm. Integument black, brown, or red, some with white, yellow or red markings on elytra or contrasting pronotum and elytra. Head: Antennal sensory elements beginning on antennomere 4; mandibles with apices bidentate or tridentate on each side. Labrum evenly convex; area between antennal fossa and compound eye unsculptured, or with carina connecting them. Frons with supra-antennal carina forked near juncture with compound eye (Fig. 4); frons with supra-orbital groove present (Fig. 1). Prothorax: Pronotum with punctures circular; sublateral incisions present, carinae present in some; posterior edge of pronotum with 3 low apices mesally; hind angles with a single carina reaching to near midlength, it is unknown whether this is the hind angle carina or the lateral carina, single carina not situated ventrad of lateral edge of pronotum; hind angles not truncate dorsally; hypomeral hind edge rectangularly emarginate (Fig. 3) immediately meso-ventrad of hind angles; procoxal cavities closed. Prosternum with sides concave in ventral view; anterior prosternal lobe covering labium; prosternal process not curved dorsad (less than 30°), ventral surface carinate laterally, or not. Mesothorax: Scutellum with anterior edge weakly concave, posterior apex rounded to pointed (Figs 7, 8). Mesosternum with anterior edges weakly concave lateral to mesosternal cavity in lateral view; mesosternal cavity with lateral edges sinuate anterad of mesocoxae. Elytral intervals not costate. Hind wings, notched in anal area. Legs: Tarsi without apically extending lobes or pads; tarsal claws each with 1 apex; metacoxal plate covers 1/2–2/3 of metatrochanter with legs withdrawn. Male genitalia: Abdominal segment 9 with tergite and sternites articulated at sides; parameres without apicolateral or apicomedial expansions, apices not forked, sides with 2 setae; aedeagus with basal struts approximately 1 times median lobe length, median lobe simple, tapered. Female genitalia: Ovipositor with baculae present; coxites flexible. Bursa copulatrix with colleterial glands indiscernible; without sclerotised spermathecae; bilobed spine-bearing sclerites present (Fig. 156); spermathecal gland duct without row of diverticulae, base not sclerotised; anterior end of bursa with 2 pedunculate sacs sharing common attachment to bursa.

##### Etymology.

Masculine. Named for a genus of Cardiophorinae known only from the southern hemisphere.

##### Discussion.

Please see text of discussion above for argumentation for new genus. No unique synapomorphies of this genus were identified. Known from Chile and Australia, 58 spp.

All Australian species are transferred from *Paracardiophorus* to *Austrocardiophorus* as: *Austrocardiophorus alternatus* Carter, 1939, *Austrocardiophorus amabilis* Carter, 1939; *Austrocardiophorus antennalis* Schwarz, 1907; *Austrocardiophorus assimilis* Carter, 1939; *Austrocardiophorus atronotatus* Carter, 1939; *Austrocardiophorus attenuatipennis* Elston, 1930; *Austrocardiophorus australis* (Candèze, 1860, *Horistonotus*); *Austrocardiophorus bicolor* (Candèze, 1878, *Cardiophorus*); *Austrocardiophorus carissimus* Carter, 1939; *Austrocardiophorus compactus* (Candèze, 1882, *Cardiophorus*); *Austrocardiophorus consobrinus* (Candèze, 1878, *Horistonotus*); *Austrocardiophorus consputus* (Candèze, 1878, *Cardiophorus*); *Austrocardiophorus cooki* Carter, 1939; *Austrocardiophorus despectus* (Candèze, 1882, *Cardiophorus*); *Austrocardiophorus dimidiatus* Schwarz, 1902; *Austrocardiophorus dissimilis* Schwarz, 1903; *Austrocardiophorus divisus* (Candèze, 1865, *Horistonotus*); *Austrocardiophorus dulcis* Carter, 1939; *Austrocardiophorus elevatus* (Van Zwaluwenburg, 1947, *Cardiophorus*); *Austrocardiophorus elisus* (Candèze, 1865, *Horistonotus*); *Austrocardiophorus eucalypti* (Blackburn, 1892, *Cardiophorus*); *Austrocardiophorus flavipennis* (Candèze, 1878, *Cardiophorus*); *Austrocardiophorus flavopictus* (Carter, 1939, *Hypnoidus*); *Austrocardiophorus fulvosignatus* (Candèze, 1878, *Cardiophorus*); *Austrocardiophorus hamatus* (Candèze, 1878, *Cardiophorus*); *Austrocardiophorus humilis* (Candèze, 1865, *Horistonotus*); *Austrocardiophorus jugulus* Elston, 1930; *Austrocardiophorus lenis* (Candèze, 1865, *Horistonotus*); *Austrocardiophorus litoralis* Carter, 1939; *Austrocardiophorus longicornis* (Candèze, 1878, *Horistonotus*); *Austrocardiophorus macleayi* (Schwarz, 1907, *Cardiophorus*); *Austrocardiophorus malkini* (Van Zwaluwenburg, 1947, *Cardiophorus*); *Austrocardiophorus mastersii* (Macleay, 1872, *Elater*); *Austrocardiophorus minimus* (Candèze, 1878, *Cardiophorus*); *Austrocardiophorus mjobergi* Elston, 1930; *Austrocardiophorus moseri* Schwarz, 1902; *Austrocardiophorus nigrosuffusus* Carter, 1939; *Austrocardiophorus occidentalis* Carter, 1939; *Austrocardiophorus octavus* (Candèze, 1878, *Cardiophorus*); *Austrocardiophorus octosignatus* Carter, 1939; *Austrocardiophorus pallidipennis* (Candèze, 1878, *Cardiophorus*); *Austrocardiophorus quadripunctatus* (Blanchard, 1853, *Agriotes*); *Austrocardiophorus quadristellatus* Carter, 1939; *Austrocardiophorus rufopictus* Carter, 1939; *Austrocardiophorus sexnotatus* Carter, 1939; *Austrocardiophorus stellatus* Carter, 1939; *Austrocardiophorus subcruciatus* Carter, 1939; *Austrocardiophorus subfasciatus* Carter, 1939; *Austrocardiophorus tumidithorax* (Schwarz, 1907, *Cardiophorus*); *Austrocardiophorus vagus* Schwarz, 1907; *Austrocardiophorus varians* Carter, 1939; *Austrocardiophorus variegatus* Schwarz, 1902; *Austrocardiophorus venustus* (Candèze, 1860, *Cardiophorus*); *Austrocardiophorus victoriensis* (Blackburn, 1892, *Cardiophorus*); *Austrocardiophorus vittipennis* Carter, 1939; *Austrocardiophorus xanthomus* (Candèze, 1865, *Horistonotus*). The following Chilean species (all) are also transferred from *Paracardiophorus* to *Austrocardiophorus*: *Paracardiophorus delfini* (Fleutiaux, 1907, *Cardiophorus*); *Austrocardiophorus elegans* (Solier, 1851, *Cardiophorus*); *Austrocardiophorus humeralis* (Fairmaire & Germain, 1860, *Cardiophorus*).

#### Chileaphricus gen. n.

Taxon classificationAnimaliaColeopteraElateridae

http://zoobank.org/5300C4DB-DB8F-4E61-977D-6C99AB44929D

Figs 38–41

##### Type species.

*Chileaphricus chilensis* (Fleutiaux, 1940)

##### Diagnosis.

Head. Labrum convex in lateral view. Prothorax. Pronotum with lateral carina absent or restricted to basal ¼ or absent; procoxal cavities open. Pterothorax. Scutellum with middle of anterior edge emarginate, posterior apex not bilobed; mesocoxal cavity open to both mesepimeron and mesepisternum (Fig. 39), mesotrochantin not hidden. Legs. Tarsomeres without ventral lobes; tarsal claws with one apex per side. Aedeagus. Aedeagus with paramere apices not forked (Fig. 41).

##### Description.

Length 9.5 mm. Integument brown. Head: Antennal sensory elements beginning on antennomere 4 (Fig. 40); mandibles with apices bidentate. Labrum evenly convex; area between antennal fossa and compound eye broadly carinate. Frons with supra-antennal carina forked near juncture with compound eye; frons with supra-orbital groove present (Fig. 2). Prothorax: Pronotum with punctures circular; sublateral carinae and incisions absent; posterior edge of pronotum with 3 apices mesally; hind angles with a single carina reaching to near midlength, it is unknown whether this is the hind angle carina or the lateral carina. Hind angles not truncate dorsally (Fig. 3); hypomeral hind edge sinuate immediately meso-ventrad of hind angles; procoxal cavities open. Prosternum with sides concave in ventral view; anterior prosternal lobe short, not covering labium; prosternal process curved dorsad more than 30°, ventral surface not carinate laterally. Mesothorax: Scutellum with anterior edge emarginate. Mesosternum with anterior edges concave lateral to mesosternal cavity in lateral view; mesosternal cavity with lateral edges sinuate anterad of mesocoxae. Mesepisternum and mesepimeron reaching mesocoxal cavity; mesotrochantin exposed in most. Elytra with intervals 4–6 costate apically (Fig. 38). Hind wings with venation well-developed, not notched in anal area. Legs: Tarsi without ventral lobes or pads; tarsal claws each with 1 apex; metacoxal plate covers 1/3 of metatrochanter with legs withdrawn. Male genitalia: abdominal segment 9 with tergite and sternites articulated at sides; parameres without apicolateral expansions (Fig. 41), apices not forked, sides with 4 setae; aedeagus with basal struts approximately 0.6 times median lobe length, median lobe simple. Females: not known.

##### Etymology.

Masculine. Named for a genus of Cardiophorinae known only from Chile.

##### Discussion.

Please see text of discussion above for argumentation for new genus. The exposed mesotrochantin is unique among Cardiophorinae. Known from: Chile, 1 sp. The type species is transferred from *Aphricus* to *Chileaphricus* as: *Chileaphricus chilensis* (Fleutiaux, 1940)

#### Paracardiophorus

Taxon classificationAnimaliaColeopteraElateridae

Schwarz, 1895b: 40

Figs 4 , 10 , 16 , 24 , 34 , 35 , 65–68

##### Type species.

*Cardiophorus musculus* Erichson, 1840: 299.

##### Diagnosis.

Head. Mandibular apices bidentate; supra antennal carina elevated with area between carina and base of labrum concave in lateral view. Prothorax. Pronotum with lateral carina not reaching anterior edge, hidden in dorsal view by overhanging edge of dorsal part of pronotum (= submarginal line). Pterothorax. Elytra all-black, or with pale markings, area between anterior-most point and humeral angle arcuate or straight in dorsal view (not sinuate) (Fig. 16), apices without shelf-like extensions. Legs. Tarsi without ventral lobes or pads extending beyond base; claws with only 1 point per side.

##### Description.

Length 4–8 mm. Integument black, some with pale spots or lines on elytra. Head: Antennal sensory elements beginning on antennomere 3; antennae not reaching pronotal hind angles; mandibles with apices bidentate. Labrum evenly convex; area between antennal fossa and compound eye unsculptured. Frons with supra-antennal carina forked near juncture with compound eye (Fig. 2); frons with supra-orbital groove present (Fig. 1). Prothorax: Pronotum with sublateral carinae and incisions present; posterior edge of pronotum with 3 apices mesally; lateral carinae not reaching anterior edge (Figs 3, 4), situated ventrad of lateral edge of pronotum; hind angles complete dorsally (Fig. 3) or truncate so the apex is composed of hypomeron only (Fig. 4); dorsal carina of hind angle present; hypomeral hind edge sinuate (Fig. 4) or rectangularly emarginate (Fig. 3) immediately meso-ventrad of hind angles; procoxal cavities open or closed. Prosternum with anterior prosternal lobe long, covering labium when head is retracted fully; prosternal process straight or curved dorsad, ventral surface carinate laterally, or not. Mesothorax: Scutellum with anterior edge emarginate (Fig. 8), posterior apex pointed (Fig. 8). Mesosternum with anterior edges concave lateral to mesosternal cavity in lateral view; mesosternal cavity with lateral edges sinuate anterad of mesocoxae. Elytral intervals not costate. Hind wings, notched in anal area. Legs: Tarsi without apically extending lobes or pads; tarsal claws each with 1 apex; metacoxal plate covers ½ to 2/3 of metatrochanter with legs withdrawn. Male genitalia: Urosternite 8 straight to bisinuate; abdominal segment 9 with tergite and sternites articulated at sides; parameres of some with apicomedial expansions, others simple, or flattened (Fig. 65), sides with 1–2 setae; aedeagus with median lobe simple (not split or expanded). Female genitalia: Ovipositor with baculae present; coxites flexible. Bursa copulatrix with colleterial glands indiscernible; without sclerotised spermathecae; pair of ovoid spine-bearing sclerites present (Figs 28, 66); spermathecal gland duct with row of diverticulae, base sclerotised inside bursa (Figs 34, 35); anterior end of bursa with 1 pedunculate sac (Fig. 27).

##### Discussion.

Please see text of discussion above for argumentation for new genus. No unique synapomorphies of this genus were identified. Known from throughout the Holarctic region, 49 spp.

Genus membership revised here to include North American spp. and exclude Australian and South American spp.

The following North American species are transferred from *Cardiophorus* to *Paracardiophorus* as: *Paracardiophorus abbreviatus* Blanchard, 1889; *Paracardiophorus acutus* Lanchester, 1971; *Paracardiophorus amplicollis* Motschulsky, 1859; *Paracardiophorus aquilis* Lanchester, 1971; *Paracardiophorus bifasciatus* Blanchard, 1889; *Paracardiophorus breviatus* Lanchester, 1971; *Paracardiophorus cardisce* (Say, 1839, *Elater*); *Paracardiophorus catskillensis* Douglas, 2003; *Paracardiophorus columbianus* Lanchester, 1971; *Paracardiophorus coxalis* Blanchard, 1889; *Paracardiophorus fenestratus* LeConte, 1859; *Paracardiophorus gemmifer* Blanchard, 1889; *Paracardiophorus ignotus* Lanchester, 1971; *Paracardiophorus kooskooskiensis* Lanchester, 1971; *Paracardiophorus luridipes* Candèze, 1860; *Paracardiophorus plebejus* Lanchester, 1971; *Paracardiophorus propinquus* Lanchester, 1971; *Paracardiophorus pullus* Blanchard, 1889; *Paracardiophorus snakensis* Lanchester, 1971; *Paracardiophorus spurius* Lanchester, 1971; *Paracardiophorus stigmaticus* Candèze, 1869; *Paracardiophorus tumidicollis* LeConte, 1853.

#### Floridelater gen. n.

Taxon classificationAnimaliaColeopteraElateridae

http://zoobank.org/D9ECC670-042B-433A-9278-136A47044044

Figs 9 , 45–47

##### Type species.

*Coptostethus americanus* Horn, 1871.

##### Diagnosis.

Pterothorax. Scutellum with anterior edge broadly concave; posterior apex bilobed (Fig. 9).

##### Description.

Length 3.8–4.5 mm. Integument grey-brown to red-brown; setae simple. Head: Antennal sensory elements beginning on antennomere 3 (Fig. 47); mandibles with apices bidentate. Labrum evenly convex; area between antennal fossa and compound eye unsculptured. Frons convex, with supra-antennal carinae concave in lateral view between carinae and labral base; supra-antennal carina forked near juncture with compound eye; frons without supra-orbital groove. Prothorax: Pronotum more than half as long as elytra, with punctures situated on tubercles; hind angles without carinae dorsally; sublateral carinae and incisions present; posterior edge of pronotum with 2 apices mesally; lateral carinae restricted to posterior half; hind angles not truncate dorsally; hypomeral hind edge sinuate (Fig. 4) immediately meso-ventrad of hind angles; procoxal cavities open. Prosternum with sides concave; anterior prosternal lobe long, covering labium in most; prosternal process curved dorsad more than 30°, ventral surface not carinate laterally. Mesothorax: Scutellum with anterior edge broadly concave, posterior apex bilobed (Fig. 9). Mesosternal cavity with lateral edges nearly straight. Elytra with intervals not costate. Hind wings area less than half of elytral area, venation not evident. Legs: Tarsi without apically extending lobes or pads; tarsal claws with one apex per side; metacoxal plate covers 1/6 of metatrochanter with legs withdrawn. Male genitalia: Abdominal segment 9 with tergite and sternites articulated at sides; parameres with apicolateral expansions, apices not forked, sides with 2 setae; aedeagus with basal struts approximately 0.8 times length of simple median lobe. Female genitalia: Ovipositor with baculae present; coxites flexible. Bursa copulatrix with colleterial glands indiscernible; without sclerotised spermathecae or sclerites; spermathecal gland duct without sclerotisation or row of diverticulae; anterior end of bursa with 2 pedunculate sacs sharing common attachment to bursa.

##### Etymology.

Masculine. Named for a genus of Cardiophorinae known only from southeastern USA.

##### Discussion.

Please see text of discussion above for argumentation for new genus. The posteriorly bilobed scutellum is unique among Elateridae examined. Known from: USA, coastal dunes by Gulf of Mexico, 1 sp. Often collected by sifting loose sand among dune vegetation.

The type species is transferred from *Negastrius* to *Floridelater* as: *Floridelater americanus* (Horn, 1871, *Coptostethus*).

### Diagnoses of tribes and genera

#### Combined diagnosis of Cardiophorinae + Negastriinae

If procoxal cavities not closed to mesepisternum and mesepimeron, then scutellum emarginate anteriorly; hind wing without wedge cell; male aedeagus with paramere bases fused together into tube both dorsally and ventrally, articulated apicad of bases, or rigid (Figs 24, 25); female ovipositor without styli.

#### Description of Cardiophorinae

This template includes much of the described morphological range of genera of Cardiophorinae and outlines variable characters for describing new genera or species of uncertain generic assignment.

**Figure 14–22.**
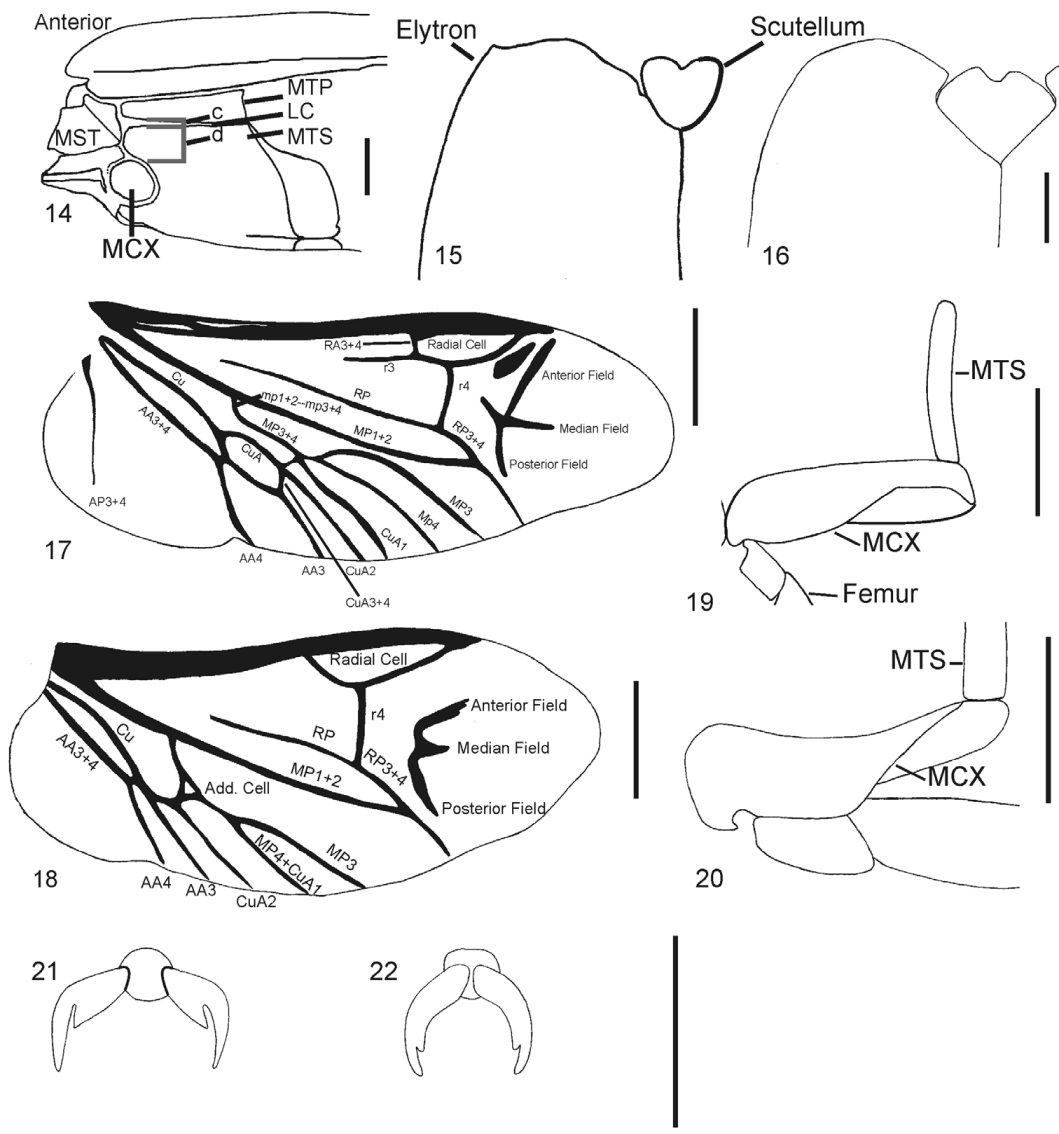
**14** Lateral view of metasternum of *Zygocardiophorus nigratissimus* Buysson Scale bar = 0.5 mm). LC = lateral carina; MTP = metepisternum; MTS = metasternum
**15–16** Dorsal view of anterior end of left elytron of Cardiophorinae Scale bar = 0.5 mm). **15** *Cardiophorus gramineus* **16** *Paracardiophorus cardisce* (Say) **17–18** Hind wing of Elateroidea Scale bar = 1 mm) **17** *Anelastes druryi* (Kirby) **18** *Blaiseus bedeli*. Vein names follow Kukalova-Peck and Lawrence (2004) **19–20** Left metacoxal plate and metepisternum of Elateroidea Scale bar = 0.5 mm). **19** *Aulonothroscus punctatus* (Bonvouloir) **20** *Cardiophorus gramineus*. MCX = metacoxal plate; MTS = metepisternum
**21–22** Metatarsal claws of Cardiophorinae Scale bar = 0.5 mm). **21** *Dicronychus cinereus* **22** *Diocarphus solitarius*. Captions reflect the revised classification.

Length 3–15 mm. Integument black, brown, yellow and/or red, some with spots or lines on pronotum or elytra; setae simple. Body without concavities for reception of tarsi. **Head**: Antennae weakly serrate; antennal sensory elements beginning on antennomere 3 or 4; mandibles with apices unidentate to tridentate. Labrum flat or evenly convex; area between each antennal fossa and adjacent compound eye unsculptured, with carina joining fossa and eye, or with groove or pit(s). Frons convex; with supra-antennal carinae complete across frontoclypeal region (Fig. 1), although not concave between carinae and labral base in some in lateral view, supra-antennal carina forked near junctures with compound eyes in many (Fig. 2); frons with supra-orbital grooves present (Fig. 2) or absent. **Prothorax**: Pronotum with punctures circular or oval, situated on tubercles in one; sublateral incisions and carinae present or absent; posterior edge of pronotum with 1 to 3 apices mesally; lateral carinae complete or not reaching anterior edge (Figs 3, 4) (possibly entirely absent in some), situated ventrad of lateral edge of pronotum in some; hind angles complete dorsally (Fig. 3), or truncate so hind angle is composed of hypomeron only (Fig. 4); dorsal carina of each hind angle single, absent to complete; hypomeral hind edges sinuate (Fig. 4) or rectangularly emarginate (Fig. 3) immediately meso-ventrad of hind angles; procoxal cavities open or closed. Prosternum with sides concave to straight in ventral view; pronotosternal sutures closed (not depressed anteriorly sufficiently to guide antennae); anterior prosternal lobe long or short, covering labium in most; prosternal process straight or curved dorsad, ventral surface carinate laterally, or not. **Mesothorax**: Scutellum with anterior edge convex (Fig. 6) to emarginate (Fig. 8), posterior apex rounded to pointed (Figs 7, 8), or bilobed (Fig. 9). Mesosternum with anterior edges convex (Fig. 14) or concave in lateral view; mesosternal cavity with lateral edges straight or sinuate anterad of mesocoxae in ventral view. Mesepisternum and mesepimeron not reaching mesocoxal cavity except in *Chileaphricus* (Fig. 13); mesotrochantin not exposed except in *Chileaphricus*. Elytra with 9 striae (weak in some), intervals flattened to costate; apices without spines. Hind wings notched in anal area or not, wedge cell absent (Fig. 18). **Legs**: Tibiae with 2 apical spurs; tarsi simple or with tarsomere 4 lobed; tarsal claws each with 1–8 apices per side; metacoxal plate covers 1/8–2/3 of metatrochanter with legs withdrawn. **Male genitalia**: Urosternite 8 straight to anteriorly pointed, with 2 lateral posterior lobes, without medial posterior lobe; abdominal segment 9 with tergite and sternites articulated at sides or base; phallobase of aedeagus not fused with parameres; parameres fused basally (Figs 24, 25), articulated in posterior half, with or without apicolateral or apicomedial expansions, apex forked in some (Fig. 25), sides with 0–30 setae; aedeagus with basal struts approximately 0.2–1.7 times median lobe length; median lobe tapered, or split, or with triangular apical expansion. **Female genitalia**: Ovipositor with baculae present or absent; coxites flexible to heavily sclerotised; styli absent. Bursa copulatrix with colleterial glands indiscernible (hemispherical in *Pachyelater*); with 0–1 coil-like sclerotised spermathecae (present in *Craspedostethus* only); with 0–1 capsule-like sclerotised spermathecae (present in *Margogastrius* only, Fig. 44); spine-bearing sclerites present (Figs 27–31), or absent; common spermathecal gland duct and spermathecal duct unmodified if present; spermathecal gland duct with row of diverticulae in some, base sclerotised inside bursa in some (Figs 34, 35); anterior end of bursa with 0–2 pedunculate sacs (Fig. 27, one sac) if 2, then sharing common attachment to bursa).

#### Diagnosis of Cardiophorinae

If procoxal cavities not closed to mesepisternum and mesepimeron, then scutellum emarginate anteriorly. Prosternum with sides near midlength straight or concave, (pronotum and prosternum also not fused). Hind wing without wedge cell; all setae evenly tapered; males with aedeagus with paramere bases fused together into tube both dorsally and ventrally, articulated apicad of bases or rigid (Figs 24, 25); females with ovipositor lacking styli.

#### Diagnoses of genera

Diagnoses presented here distinguish each genus from all other Cardiophorinae. Diagnoses for newly described and redescribed genera are provided above with corresponding genus description.

##### Allocardiophorus

Taxon classificationAnimaliaColeopteraElateridae

Ôhira, 1989

Allocardiophorus Ôhira 1989: 79. Type species: Paracardiophorus nigroapicalis Miwa, 1927: 109.

###### Diagnosis.

Head. Mandibles bidentate. Prothorax. Pronotum with lateral carina reaching more than halfway to anterior edge, not hidden by lateral expansion of pronotum in dorsal view; prosternum with anterior edge produced as lobe, concealing labium when head not extended; procoxal cavities open. Pterothorax. Scutellum with anterior edge emarginate (Fig. 10), posterior end pointed. Legs. Tarsi without lobes, tarsal claws each with a single point per side. Also. Bursa copulatrix with paired proximal sclerites partially membranous between spines. Known from Japan (Ryukyu Islands), People’s Republic of China: Hubei, Sichuan, Taiwan, 2 spp. Specimens not available for examination.

##### Aphricus

Taxon classificationAnimaliaColeopteraElateridae

LeConte, 1853

Figs 54–56

Aphricus LeConte, 1853: 501. Type species: Aphricus californicus LeConte, 1853: 502.
Patriciella Van Zwaluwenburg, 1953: 20. **Syn. n.**
Patriciella Replacement name for Patricia Van Zwaluwenburg.
Patricia Van Zwaluwenburg, 1947: 113. Type species: Patricia australicaVan Zwaluwenburg 1947: 114.

###### Diagnosis.

Head. Labrum flat in lateral view. Prothorax. Pronotum with lateral carina absent or restricted to basal ¼; procoxal cavities open. Pterothorax. Scutellum with middle of anterior edge convex to broadly concave posterior apex not bilobed. Legs. Tarsomeres without ventral lobes; tarsal claws with one apex per side. Aedeagus. Aedeagus with paramere apices not forked (Fig. 54). Also. Prosternum with anterior edge short, exposing labium. Pronotum with setae on disc not on tubercles. Mesepisternum with anteromesal corners rounded. Hind wing with venation well developed; anterior edge of elytra rounded (Fig. 55), not sinuate, in dorsal view. Known from USA, Australia, New Zealand. Females unknown. 8 spp.

##### Aptopus

Taxon classificationAnimaliaColeopteraElateridae

Eschscholtz, 1829

Figs 93–97

Aptopus Eschscholtz, 1829: 32. Type species: Aptopus tibialis Eschscholtz, 1829: 32.
Aptopus Definition restricted here to exclude species near Aptopus agrestis (Erichson).

###### Diagnosis.

Prothorax. Pronotum with lateral carina not reaching anterior edge, hidden in dorsal view by overhanging edge of dorsal part of pronotum (= submarginal line). Legs. Tarsi without apically extending lobes or pads, tarsal claws with 5 or more points per side. Also. Bursa copulatrix with both proximal (Fig. 95) and distal sclerites (Fig. 96). Known from: Argentina to southwestern North America, 35 spp.

##### Blaiseus

Taxon classificationAnimaliaColeopteraElateridae

Fleutiaux, 1931

Figs 7 , 18 , 25 , 45–47

Blaiseus Fleutiaux, 1931: 307. Type species: Blaiseus bedeliFleutiaux 1931: 308.

###### Diagnosis.

Mandibles simple; aedeagus with parameres split vertically into dorsal and ventral lobes (Fig. 25). Male abdominal segment 9 with tergite and sternite articulated at base. Most species with tibiae broadened (Fig. 49), apparently for digging. Known female brachypterous; bursa copulatrix without sclerotised structures. Known from: Vietnam, PR China, Laos, Malaysia, South Africa, Mexico, Guatemala, Honduras, 6 spp.

##### Buckelater

Taxon classificationAnimaliaColeopteraElateridae

Costa, 1973

Figs 158–160

Buckelater Costa, 1973: 33. Type species: Buckelater argutus Costa, 1973: 35.

###### Diagnosis.

Distance between antennae equal to only ¼ width of head (measured across outsides of compound eyes), nasale oriented ventrally (Fig. 159). Also: procoxal cavities closed; hind wing membrane notched in anal area. Known from: Brazil, males only, 1 sp.

##### Cardiodontulus

Taxon classificationAnimaliaColeopteraElateridae

Van Zwaluwenburg, 1963

Figs 147–150

Cardiodontulus Van Zwaluwenburg, 1963: 341. Type species: Cardiodontulus brandti Van Zwaluwenburg, 1963: 341.

###### Diagnosis.

Prothorax. Pronotum with lateral carina extending anterad from hind angles following lateral edge, not reaching anterior edge; procoxal cavities closed. Pterothorax. Scutellum with middle of anterior edge concave: broadly or abruptly emarginate. Legs. Tarsomere 4 lobed or lamellate; Tarsal claws with 2 points per side (Fig. 150). Also. Apical half of elytral interval 9 not costate. Known from: Papua New Guinea, Australia. Only males known, 2 spp.

##### Cardiophorellus: subgenusCardiophorellus

Taxon classificationAnimaliaColeopteraElateridae

Cobos, 1970

Figs 85–87

Cardiophorellus Cobos, 1970a: 222. Type species: Cardiophorellus gracilicornis Cobos, 1970a: 223.

###### Diagnosis.

Head. Mandibular apex unidentate (simple). Prothorax. Pronotum with lateral carina present but hidden in dorsal view by swollen pronotum (= submarginal line). Pterothorax. Scutellum with middle of anterior edge broadly concave. Also: Head with supra-orbital groove present; posterior edges of hypomeron mesad of hind angles with rectangular or semicircular indentations; prosternum with anterior edge short, exposing labium; tarsomere 4 without ventral lobe or pad extending beyond base of tarsomere 5; tarsal claws with 1 point per side; tibiae with posterior surfaces convex, weakly modified for digging (Figs 85, 86). Females undescribed or unassociated. Known from: Republic of the Congo, 1 sp.

##### Cardiophorellus: subgenusParapleonomus

Taxon classificationAnimaliaColeopteraElateridae

Cobos, 1970

Cardiophorellus : subgenus Parapleonomus Cobos, 1970a: 222. Type species: Cardiophorellus inermis Cobos, 1970a: 222.
Cardiophorellus Diagnosis inferred from Cobos, 1970a. Insufficient information available for inclusion in key to genera.

###### Diagnosis.

Head. Mandibular apex bidentate or multidentate; supra–orbital groove present. Prothorax. Pronotum without lateral carina, or apparently hind-angle carina; prosternum with anterior edge short, exposing labium. Legs. Tarsomere 4 without ventral lobe or pad extending beyond base of tarsomere 5; tarsal claws with 1 point per side; tibiae with posterior surfaces convex, modified for digging. Aedeagus. Parameres with apices undivided. Females undescribed or unassociated. Known from: South Africa, Gauteng Province, 1 sp.

##### Cardiophorus: subgenusCardiophorus

Taxon classificationAnimaliaColeopteraElateridae

Eschscholtz, 1829

Figs 1 , 2 , 3 , 5 , 8 , 15 , 20 , 27 , 32 , 33 , 81–84

Cardiophorus Eschscholtz, 1829: 34. Type species: Elater thoracicus Fabricius, 1801: 236, now a synonym of Cardiophorus gramineus (Scopoli, 1763).
Caloderus Stephens, 1830: 269. Type species: Elater thoracicus Fabricius, 1801: 236.
Melanotus Gistel, 1834: 11, not Eschscholtz, 1829: 32. Type species Elater thoracicus Fabricius, 1801: 236, designated by Sánchez (1996) [not in References].
Paradicronychus Dolin and Gurjeva, 1975: 116 (*nomen nudum*) —Here placed as **syn. n.** of Cardiophorus.

###### Diagnosis.

Head. Mandibles with two or three apices. Prothorax. Pronotum with lateral carina not reaching anterior third (Fig. 3), hidden in dorsal view by overhanging edge of dorsal part of pronotum (= submarginal line). Pterothorax. Scutellum with anterior edge emarginate (Fig. 8); Edge of elytra in dorsal view between anterior-most point and humeral angle sinuate or tuberculate (Fig. 15). Legs. Tarsi without ventral lobes; and tarsal claws with one apex per side. Also. Labrum convex in lateral view. Bursa copulatrix with proximal (largest, or only) sclerites ovoid (Fig. 83); base of spermathecal gland duct not sclerotised (Fig. 33 shows adjacent sclerite). Paraphyletic, known from: North America, Eurasia, Africa, 547 spp.

##### Cardiophorus: subgenusCoptostethus

Taxon classificationAnimaliaColeopteraElateridae

Wollaston, 1854

Fig. 100

Coptostethus Wollaston, 1854: 238. Type species: Cardiophorus femoratus Wollaston, 1854: 240.

###### Diagnosis.

Head. Mandibles with 2 points. Prothorax. Pronotum with carina along lateral edge hidden or not in dorsal view; restricted to hind angles, or reaching less than halfway to anterior edge. Prosternum with anterior edge not short, produced as lobe, concealing labium when head not extended. Pterothorax. Scutellum with middle of anterior edge abruptly emarginate, anterolateral edges evenly rounded, and posterior apex pointed. Legs. No tarsomeres lobed or lamellate; tarsal claws with one apex per side. Also. Pterothorax. Brachypterous. Abdomen. Urosternites 3–7 without serrations along sides.

###### Type locality.

Porto Santo Island, Madeira Archipelago, from a cave, females unknown. The relatedness of this species to brachypterous Cardiophorinae from the Canary Archipelago. and South Africa also placed in subgenus Coptostethus has not been demonstrated. Some *Coptostethus* spp. from the Canary Archipelago have tarsal claws with 2 apices (Fig. 21), and would key here to *Dicronychus*. Includes 41 spp.

##### Cardiophorus: subgenusPerrinellus

Taxon classificationAnimaliaColeopteraElateridae

Buysson, 1899

Figs 98–99

Cardiophorus : subgenus Perrinellus Buysson, 1899: 282. Type species: Athous argentatus Abeille de Perrin, 1894: 92.
Cardiophorus : subgenus Lasiocerus Buysson, 1912: 129. Type species: Cardiophorus schusteri Buysson, 1912: 128.

###### Diagnosis.

Head. Mandibles with 2 apices; supra-antennal carina without longitudinal split next to compound eyes. Prothorax. Pronotum with lateral carina reaching less than halfway to anterior edge (in type species not distinguishable from the dorsal hind angle carina), not hidden by lateral expansion of pronotum in dorsal view; prosternum with anterior edge produced as lobe, concealing labium when head not extended. Pterothorax. Scutellum with anterior edge emarginate, anterolateral edges broadened posterior to anterolateral corners, posterior end pointed. Legs. tarsi without ventral lobes and tarsal claws with one apex per side. Also. Pronotum with hind angles not truncate dorsally; procoxal cavities open. Aedeagus with parameres approximately cylindrical pre-apically (Fig. 99).

###### Type locality.

Israel, other spp from North Africa, Ceylon, Central Asia, probably not monophyletic, 12 spp. Cardiophorus (Lasiocerus) du Buysson was not located for examination, and may not match these key characteristics. Subgenus Lasiocerus was described from Azerbaijan, and distinguished by its long antennae with dense setae.

##### Cardiotarsus

Taxon classificationAnimaliaColeopteraElateridae

Eschscholtz, 1836

Figs 116–118

Cardiotarsus Eschscholtz, 1836: published in identification table opposite p.5, without associated spp. Type species: Cardiotarsus capensis Candèze, 1860: 226.

###### Diagnosis.

Pronotum. Lateral carina not reaching anterior edge (Fig. 117), hidden in dorsal view by overhanging edge of dorsal part of pronotum (= submarginal line). Legs. Tarsomere 4 with ventral lobe or pad extending beyond base of tarsomere 5; Tarsal claws with one apex per side. Also proximal (largest) sclerites of bursa copulatrix ovoid (Fig. 118, right). Known from Africa, Mauritius, southern and eastern Asia, Japan, Taiwan, 51 spp.

##### Craspedostethus

Taxon classificationAnimaliaColeopteraElateridae

Schwarz, 1898

Figs 151–154

Craspedostethus Schwarz, 1898b: 414. Replacement name for Craspedonotus Schwarz.
Craspedonotus Schwarz, 1898a: 148. Type species: Craspedostethus rufiventris Schwarz, 1898a: 148.

###### Diagnosis.

Head. Mandibular apex bidentate or tridentate. Prothorax. Pronotum with lateral carina reaching ¾ of distance to anterior edge, hidden in dorsal view by overhanging edge of dorsal part of pronotum (Fig. 152). Legs: tarsomere 4 without ventral lobe or pad extending beyond base of tarsomere 5; tarsal claws with one apex per side. Elytra: with edge in dorsal view between anterior-most point and humeral angle sinuate or tuberculate. Also: Head: dorsal surface of labrum flat in side view. Female bursa copulatrix with a pair of semi-membranous concave sclerites (Fig. 154), or none; base of spermathecal gland duct not sclerotised. Known from Cameroon to Iran, 19 spp.

##### Dicronychus

Taxon classificationAnimaliaColeopteraElateridae

Brullé, 1832

Figs 21 , 88–89 , 90–92

Dicronychus Brullé, 1832: 138. Type species: Elater obesusBrullé 1832: 138, now referred to by replacement name Dicronychus brulleiPlatia and Gudenzi (2003).
Paramecus Dillwyn, 1829: 32. Type species: Paramecus cordiger Dillwyn, 1829 (= Elater equiseti Herbst, 1784: 114.

###### Diagnosis.

Prothorax. Pronotum with lateral carina not reaching anterior edge, hidden in dorsal view by overhanging edge of dorsal part of pronotum (= submarginal line); procoxal cavities open. Legs. Tarsomere 4 without ventral lobe or pad extending beyond base of tarsomere 5; tarsal claws with second apex at base on each side (Fig. 21). Also hind wing notched in anal area; proximal (largest) sclerites of bursa copulatrix ovoid (Fig. 90). Known from Eurasia, Africa, 134 spp. Monophyly unknown, has been confused with *Platynychus* by some. Some brachypterous spp. currently assigned to *Cardiophorus* s.g. *Coptostethus* may belong here.

##### Diocarphus

Taxon classificationAnimaliaColeopteraElateridae

Fleutiaux, 1947

Figs 22

Diocarphus Fleutiaux, 1947a: 364. **stat. n.**, raised to genus rank.
Phorocardius : subgenus Diocarphus Fleutiaux, 1947a: 364. Type species: Phorocardius solitarius Fleutiaux, 1931: 309.

###### Diagnosis.

Head. Head with single pit between antennal fossa and eye. Prothorax. Pronotum with lateral carina not reaching anterior edge, hidden in dorsal view by overhanging edge of dorsal part of pronotum (= submarginal line); procoxal cavities closed. Pterothorax. Anterior edge of scutellum emarginate. Legs. Tarsomere 4 without ventral lobe or pad extending beyond base of tarsomere 5; tarsal claws with two apices per side.

Also. Legs. tarsal claws with ventral surface concave mesad of basal apex (Fig. 22). Bursa Copulatrix. Proximal (largest) sclerites ovoid (Fig. 113, right); paired distal sclerites absent (*i.e.* a second pair, farther from vagina, at base of spermathecal gland duct); base of spermathecal gland duct with tube-like sclerotisation, (Fig. 113, left), without paired plate-like appendages attached. Known from Vietnam, 1 sp.

##### Displatynychus

Taxon classificationAnimaliaColeopteraElateridae

Ôhira, 1987

Figs 109–112

Displatynychus Ôhira, 1987: 92.
Platynychus : subgenus Displatynychus Ôhira, 1987: 92. Type species: Cardiophorus adjutor Candèze, 1875: 17.

###### Diagnosis.

Head. Area between antennal fossa and compound eye with carina connecting fossa and eye, or with 2 pits with non-depressed area between them. Prothorax. Pronotum with lateral carina not reaching anterior edge, hidden in dorsal view by overhanging edge of dorsal part of pronotum (= submarginal line); procoxal cavities closed. Pterothorax. Anterior edge of scutellum emarginate. Legs. Tarsomere 4 without ventral lobe or pad extending beyond base of tarsomere 5; tarsal claws with two apices per side. Also. Legs. Tarsal claws with ventral surface convex mesad of basal apex. Bursa Copulatrix. Proximal (largest) sclerites ovoid; paired distal sclerites absent (*i.e.* a second pair, farther from vagina, at base of spermathecal gland duct); base of spermathecal gland duct with tube-like sclerotisation (Fig. 111), without paired plate-like appendages. Known from PR China, Japan, and South Korea, 2 spp.

##### Esthesopus

Taxon classificationAnimaliaColeopteraElateridae

Eschscholtz, 1829

Figs 6 , 31 , 126–131

Esthesopus Eschscholtz, 1829: 32. Type species: Esthesopus castaneus Eschscholtz, 1829: 32.

###### Diagnosis.

Prothorax. Pronotum with carina extending anterad from hind angles following lateral edge (ie, not below lateral edge of prothorax, but in some species not distinguishable from the dorsal hind angle carina), not reaching anterior edge. Pterothorax. Scutellum with middle of anterior edge straight (Fig. 6). Legs. Tarsi with tarsomere 4 lobed or lamellate (Fig. 128), claws with 2 points each (Fig. 129). Also. Bursa copulatrix with paired proximal (largest) sclerites bilobed (Fig. 130) to multi-lobed (Fig. 31). Known from South and North America, and the Greater and Lesser Antilles, 50 spp.

##### Globothorax

Taxon classificationAnimaliaColeopteraElateridae

Fleutiaux, 1891

Figs 30 , 77–78 , 79–80

Globothorax Fleutiaux, 1891: ccxxxii. Type species: Globothorax chevrolati Fleutiaux, 1891: ccxxxiii.
Teslasena Fleutiaux, 1892: 410. **Syn. n.** Type species, Anelastes femoralis Lucas, 1857: 71.

###### Diagnosis.

Legs. Tibiae flattened and broadened apically (Figs 78, 80), apparently for digging, tarsi without apically extending lobes or pads, tarsal claws with 2–3 apices per side. Also, posterior edge of pronotum bidentate mesally; anterior edge of scutellum straight, females with compound eyes nearly flat and antennae reaching only 2/3 of distance to pronotal hind angles; bursa copulatrix with proximal (only) sclerites parallel sided (Fig. 30). Known from: Brazil, Bolivia, 3 spp.

##### Horistonotus

Taxon classificationAnimaliaColeopteraElateridae

Candèze, 1860

Figs 132–139

Horistonotus Candèze, 1860: 243. Type species: Horistonotus flavidus Candèze, 1860: 250.

###### Diagnosis.

Prothorax. Pronotum with carina extending anterad from hind angles following lateral edge (ie, not below lateral edge of prothorax, but in some species not distinguishable from the dorsal hind angle carina), not reaching anterior edge. Legs. Tibiae not modified for digging; tarsi with tarsomere 4 not lobed or lamellate, tarsal claws with 2 or 7 points per side (Fig. 134). Also. Bursa copulatrix with paired proximal (largest) sclerites ovoid (Fig. 138). Known from South and North America, and the Antilles, 106 spp.

##### Margogastrius

Taxon classificationAnimaliaColeopteraElateridae

Schwarz, 1903

Figs 42–44

Margogastrius Schwarz, 1903b: 80. Replacement name for Gastrimargus Schwarz, 1902.

###### Diagnosis.

Head. Mandibular apex unidentate (simple); supra-orbital groove present. Prothorax. Pronotum with lateral carina not reaching anterior edge, hidden in dorsal view by overhanging edge of dorsal part of pronotum (= submarginal line). Pterothorax. Scutellum with middle of anterior edge abruptly emarginate. Legs. Tarsal claws with one point per side. Also. Posterior edges of hypomeron mesad of hind angles with rectangular or semicircular indentations; prosternum with anterior edge not short, produced as lobe, concealing labium when head retracted; tibiae with posterior surfaces flattened and broadened apically (Fig. 42), apparently strongly modified for digging; Tarsomere 4 without ventral lobe or pad extending beyond base of tarsomere 5; proximal (largest) sclerites of bursa copulatrix reduced, capsule-like spermatheca attached to ventral surface of bursa by short duct (Fig. 44). Known from Tanzania, females only, 1 sp.

##### Metacardiophorus

Taxon classificationAnimaliaColeopteraElateridae

Gurjeva, 1966

Figs 62 , 63–64

Metacardiophorus Gurjeva, 1966: 91. **stat. n.**, raised to genus rank.
Cardiophorus : subgenus Metacardiophorus Gurjeva, 1966: 91. Type species: Cardiophorus sogdianus Gurjeva, 1966: 91.

###### Diagnosis.

Head. Mandibles with apices unidentate. Prothorax. Pronotum with lateral carina not reaching anterior edge, extending anterad from hind angles following lateral edge (Fig. 63) or completely absent (not below lateral edge); prosternum with anterior edge not short, produced as lobe, concealing labium when head retracted. Pterothorax. Scutellum with middle of anterior edge abruptly emarginate. Legs. No tarsomeres lobed or lamellate; tarsal claws with one apex per side; tarsal claws with one apex per side. Also. Pterothorax. Scutellum pointed at posterior apex. Females unknown. Known from Uzbekistan and Tajikistan, 3 spp.

##### Mionelater

Taxon classificationAnimaliaColeopteraElateridae

Becker, 1963 known from fossil specimen only

Mionelater Becker, 1963: 125. Type species: Mionelater planatus Becker, 1963: 126.

###### Diagnosis.

Head. Supra antennal carina porrect in lateral view; eyes large; antennae serrate with acute apicoventral angles. Prothorax. Hind angles elongate, pronotum abruptly constricted at base. Pterothorax. Mesocoxal cavity possibly open to mesepisternum and mesepimeron. Legs. No tarsomeres lobed or lamellate; tarsal claws with one apex per side; tarsal claws with one apex per side. Not in key to species.

Comment: the serrate antennae, large eyes, shelf-like supra-antennal carina, elongate pronotal hind angles, and open mesocoxal cavities suggest this genus might belong to Dendrometrinae or another subfamily. One known species.

##### Neocardiophorus

Taxon classificationAnimaliaColeopteraElateridae

Gurjeva, 1966

Figs 57–58

Neocardiophorus Gurjeva, 1966: 95. Type species: Neocardiophorus mamajevi Gurjeva, 1966: 95.

###### Diagnosis.

Head. Mandibles with 2 points. Prothorax. Pronotum with carina along lateral edge not hidden in dorsal view, and restricted to hind angles, or not reaching more than halfway to anterior edge. Prosternum with anterior edge not short, produced as lobe, concealing labium when head not extended. Pterothorax. Scutellum with middle of anterior edge abruptly emarginate, anterolateral edges evenly rounded, and posterior apex evenly rounded. Legs. No tarsomeres lobed or lamellate; tarsal claws with one apex per side; tarsal claws with one apex per side. Also. Pterothorax. Abdomen. Urosternites 3–7 without serrations along sides. Two species, known from males from Turkmenistan and Uzbekistan.

##### Nyctor

Taxon classificationAnimaliaColeopteraElateridae

Semenov-Tian-Shanskij & Pjatakova, 1936

Figs 59–61

Nyctor Semenov-Tian-Shanskij & Pjatakova, 1936: 101 Nyctor expallidus Semenov-Tian-Shanskij & Pjatakova, 1936: 102.

###### Diagnosis.

Head. Head with area between antenna fossa and compound eye unsculptured. Prothorax. Pronotum with lateral carina extending anterad from hind angles following lateral edge, not reaching anterior edge; prosternum with anterior edge short, exposing labium. Pterothorax. Scutellum with middle of anterior edge abruptly emarginate. Legs. No tarsomeres lobed or lamellate; tarsal claws with one apex per side; tarsal claws with one apex per side. Also. Hind wing. Membrane notched in anal area (between AA3+4 and AP). Not reduced in female. Female genitalia. Proximal (largest) sclerites of bursa copulatrix ovoid; distal sclerites absent. Male genitalia. Parameres with 2 setae each. Known from Tajikistan, Turkmenistan, and Uzbekistan, 2 spp.

##### Odontocardus

Taxon classificationAnimaliaColeopteraElateridae

Fleutiaux, 1931

Figs 119–121 , 122

Odontocardus Fleutiaux, 1931: 332. Type species: Cardiotarsus vitalisi Fleutiaux, 1918b: 231.

###### Diagnosis.

Prothorax. Pronotum with lateral carina not reaching anterior edge, hidden in dorsal view by overhanging edge of dorsal part of pronotum (= submarginal line). Legs. Tarsomere 4 with ventral lobe or pad extending beyond base of tarsomere 5; tarsal claws with both basal and apical points on each side. Also. Bursa copulatrix with paired proximal (largest) sclerites of bursa copulatrix ovoid. Known from Cambodia, Vietnam, Laos, Philippines, 6 spp.

##### Pachyelater

Taxon classificationAnimaliaColeopteraElateridae

Lesne, 1897

Figs 50–53

Pachyelater Lesne, 1897b: 117 Replacement name for Parelater Lesne, 1897a. Transferred here to Cardiophorinae.
Lesnelater Fleutiaux, 1935a: 116. Type species Lesnelater madagascariensisFleutiaux 1935a, a synonym of Pachyelater madagascariensis Lesne, 1897 (Douglas 2011)
Parelater Lesne 1897a: 102. Pachyelater madagascariensis Lesne, 1897: 102. Preoccupied by Parelater Candèze, 1882: 70 (Coleoptera: Elateridae).

###### Diagnosis.

Head. Mandibles bidentate; labrum convex in lateral view. Prothorax. Lateral carina not reaching anterior edge, not hidden in dorsal view; procoxal cavities open; prosternum with anterior edge short, exposing labium; pronotum with setae on disc not on tubercles. Mesothorax. Scutellum with anterior edge broadly concave, posterior end not bilobed; mesepisternum with anteromesal corners rounded; anterior edge of elytra rounded or straight in dorsal view. Legs. Protibiae flattened and broadened apparently for digging (Figs 50, 51); tarsi without ventral lobes and tarsal claws with one apex per side. Also, males with aedeagus parameres undivided (Fig. 53). Females with compound eyes reduced (Fig. 50); antennae reaching only halfway to pronotal hind angles; ovipositor reduced, with baculae shorter than coxites; bursa copulatrix without sclerites. Known from: Madagascar, Southern Africa, 6 spp.

##### Paraplatynychus

Taxon classificationAnimaliaColeopteraElateridae

Fleutiaux, 1931

Figs 29 , 140–143

Platynychus : subgenus Paraplatynychus Fleutiaux, 1931: 315. Type species: Paraplatynychus mixtus Fleutiaux, 1931: 326.

###### Diagnosis.

Prothorax. Pronotum with complete carina at lateral edge (Fig. 141), reaching from hind angle to anterior edge of prothorax. Pterothorax. Scutellum with middle of anterior edge broadly concave. Also. Tarsal claws with both basal and apical points on each side, without basal setae; bursa copulatrix with proximal sclerites bilobed (Fig. 142). Diagnosis is based on type species. *Platynychus fuscipennis* Candèze, 1860, and *Platynychus incostatus* Fleutiaux, 1931 have lateral carina of pronotum reaching only 9/10 to anterior edge of pronotum, and scutellum mesally notched. Known from PR China, and Southeast Asia, 4 spp.

##### Phorocardius

Taxon classificationAnimaliaColeopteraElateridae

Fleutiaux, 1931

Figs 101–103

Phorocardius Fleutiaux, 1931: 308. Type species: Cardiophorus florentini Fleutiaux, 1895a: 687.

###### Diagnosis.

Prothorax. Pronotum with lateral carina not reaching anterior edge (Fig. 102), hidden in dorsal view by overhanging edge of dorsal part of pronotum (= submarginal line); procoxal cavities open. Legs. Tarsomere 4 without ventral lobe or pad extending beyond base of tarsomere 5; tarsal claws with second point near apex on each side. Also hind wing not notched in anal area; proximal (largest) sclerites of bursa copulatrix ovoid (Fig. 103). Known from Burma, PR China, India, Laos, Nepal, Taiwan, Thailand, and Vietnam, 13 spp.

##### Platynychus

Taxon classificationAnimaliaColeopteraElateridae

Motschulsky, 1858

Figs 74–76

Platynychus Motschulsky, 1858: 58. Type species: Platynychus indicus Motschulsky, 1858: 59.

###### Diagnosis.

Head. Head with area between antenna fossa and compound eye unsculptured. Prothorax. Pronotum with lateral carina not reaching anterior edge (Fig. 75), hidden in dorsal view by overhanging edge of dorsal part of pronotum (= submarginal line); procoxal cavities closed. Pterothorax. Scutellum with anterior edge sharply emarginate. Legs. Tarsomere 4 without ventral lobe or pad extending beyond base of tarsomere 5; tarsal claws with second point (near base). Also. Bursa copulatrix with paired proximal (largest) sclerites ovoid (Fig. 76); paired distal sclerites (pair farthest from vagina, at base of spermathecal gland duct) present and fused into a “U” shape; base of spermathecal gland duct inside bursa without tube-like sclerotisation. Known from the Russian Far East to Japan, India, and Irian Jaya, 18 spp.

##### Ryukyucardiophorus

Taxon classificationAnimaliaColeopteraElateridae

Ôhira, 1973

Figs 161–162

Ryukyucardiophorus Ôhira, 1973a: 32. Type species: Paracardiophorus loochooensis, Miwa 1934: 255.

###### Prothorax.

Pronotum with carina extending anterad from hind angles following lateral edge (ie, not below lateral edge of prothorax, but in some species not distinguishable from the dorsal hind angle carina), not reaching anterior edge. Legs. Protibiae not modified for digging; tarsi with tarsomere 4 not lobed or lamellate, claws with 2 points per side. Bursa copulatrix with paired proximal (largest) sclerites bilobed with attached semi-sclerotised membrane with spines (Fig. 162). Also. Procoxal cavities apparently closed; aedeagus parameres apices without lateral expansions. Known from Japan, and Taiwan, 4 spp.

##### Triplonychoidus

Taxon classificationAnimaliaColeopteraElateridae

Schwarz, 1906

Figs 123–125

Triplonychoidus Schwarz, 1906: 181. Type species: Triplonychus trivittatus Champion, 1895: 427.

###### Diagnosis.

Prothorax. Pronotum with lateral carina extending anterad from hind angles following lateral edge, not reaching anterior edge; procoxal cavities open. Pterothorax. Scutellum with middle of anterior edge concave: (broadly or abruptly emarginate). Legs. Tarsomere 4 lobed or lamellate; tarsal claws with two apices per side. Also. Apical half of elytral interval 9 costate. Known from Mexico to South America, females unknown, 2 spp.

##### Triplonychus

Taxon classificationAnimaliaColeopteraElateridae

Candèze, 1860

Figs 144–146

Triplonychus Candèze, 1860: 236. Type species: Triplonychus acuminatus Candèze, 1860: 238.

###### Diagnosis.

Prothorax. Pronotum with carina extending anterad from hind angles following lateral edge (ie, not below lateral edge of prothorax, but in some species not distinguishable from the dorsal hind angle carina), not reaching anterior edge. Legs. Protibiae not modified for digging; tarsi with tarsomere 4 not lobed or lamellate, claws with 3 points per side. Also. Head with area between antenna fossa and compound eye with either carina connecting fossa and eye, or with 2 pits with non-depressed area between. Elytra with apical half of intervals 1–8 costate (Fig. 145). Abdomen. Urosternite 7 (ventrite 5) with second carina mesad of lateral carina. Bursa copulatrix with paired proximal (largest) sclerites elongate, parallel sided. Known from South and Central America, 17 spp.

##### Tropidiplus

Taxon classificationAnimaliaColeopteraElateridae

Fleutiaux, 1903

Figs 104 , 105–108

Tropidiplus Fleutiaux, 1903: 251. Type species: Tropidiplus tellinii Fleutiaux, 1903: 251.

###### Diagnosis.

Prothorax. Pronotum with complete lateral carina (Fig. 105), reaching from hind angle to anterior edge of prothorax (displaced ventrad in some). Legs. Tarsal claws with or without basal point on each side. Also. Scutellum with anterior edge abruptly emarginate; tarsal claws with basal setae (Fig. 106, possibly absent in some); bursa copulatrix with proximal sclerites elongate-ovoid (Fig. 107). In type species (*Tropidiplus tellinii*) urosternite 7 has multiple longitudinal grooves and second longitudinal carina near lateral edge. Known from Eritrea, Ethiopia, Mozambique, and Kenya, 4 spp.

##### Zygocardiophorus

Taxon classificationAnimaliaColeopteraElateridae

Iablokoff-Khnzorian & Mardjanian, 1981

Figs 14 , 69–73

Zygocardiophorus Iablokoff-Khnzorian & Mardjanian, 1981: 247. Type species, Cardiophorus nigratissimus Buysson, 1891: 134. **Stat. n.** raised to genus rank.

###### Diagnosis.

Head. Mandibular apices tridentate; supra antennal carina low with area between carina and base of labrum not concave in lateral view, carina not forked beside compound eye (Fig. 1). Prothorax Pronotum with lateral carina not reaching anterior edge, hidden in dorsal view by overhanging edge of dorsal part of pronotum (= submarginal line). Pterothorax. Elytra all-black, area between anterior-most point and humeral angle arcuate or straight in dorsal view (not sinuate), apices with or without shelf-like epipleural extensions (Fig. 71). Legs. Tarsi without ventral lobes or pads extending beyond base; claws with only 1 point per side. Also. Bursa copulatrix with paired proximal (largest) sclerites ovoid with long spines; base of spermathecal gland duct sclerotised, but without paired plate-like appendages. Known from Georgia, and Turkey, east to Turkmenistan and Iran, 1 sp.

## Plates

**Figures 23–35.**
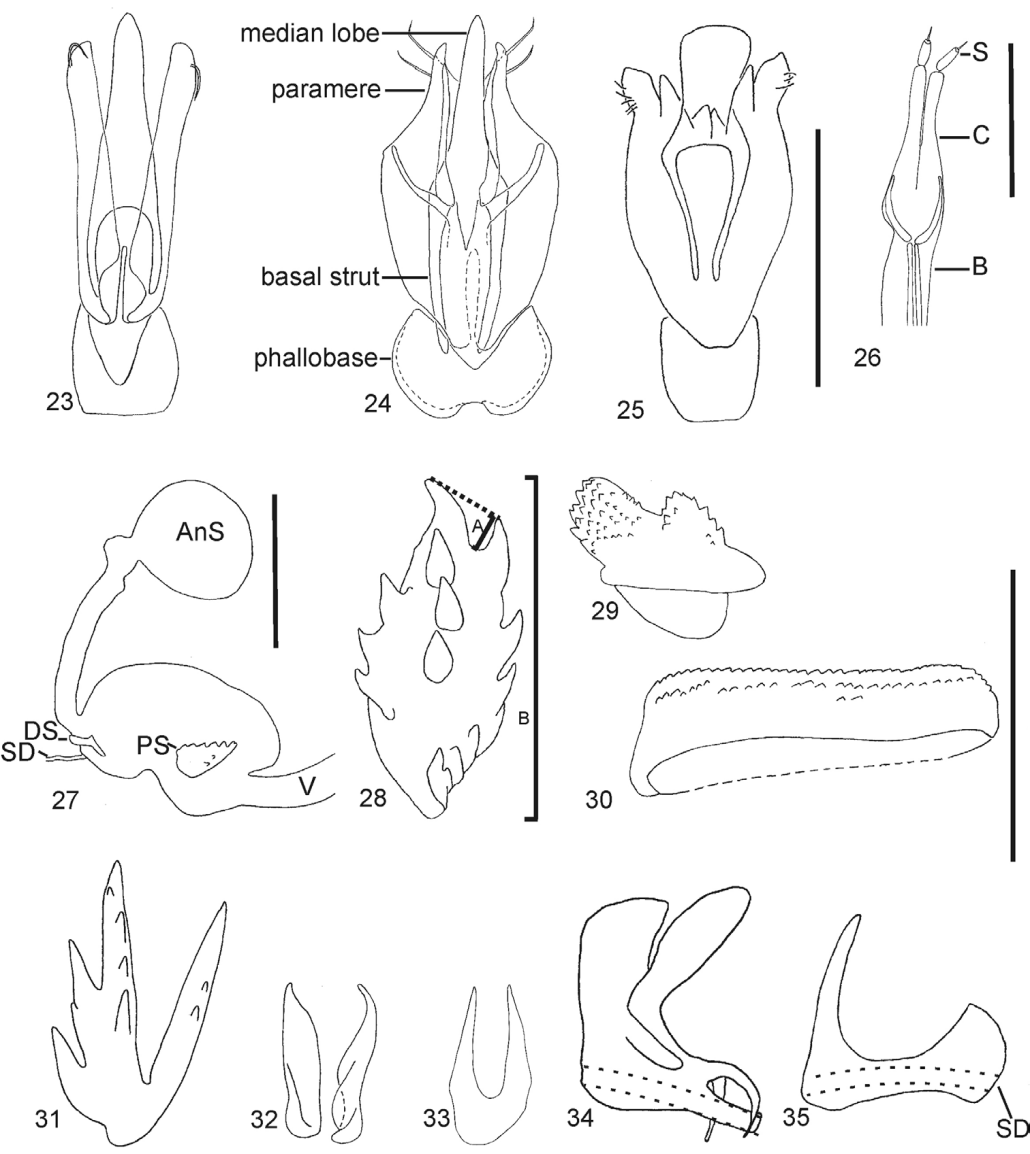
**23–25** Dorsal view of aedeagus of Elateridae Scale bar = 0.5 mm). **23** *Hypnoidus riparius* Fabricius, 1792 [not Cardiophorinae] **24** *Paracardiophorus cardisce* **25** *Blaiseus bedeli* **28–31** Proximal sclerites of the bursa copulatrix of Cardiophorinae, interior view (top end of sclerite extends furthest into bursa) Scale bar = 0.5 mm). **28** *Paracardiophorus cardisce* **29** *Paraplatynychus mixtus* **30** *Globothorax chevrolati* **31** *Esthesopus parcus* Horn **32–33** Distal sclerites of the bursa copulatrix of Cardiophorinae, interior view Scale bar = 0.5 mm). **32** *Cardiophorus inflatus* **33** *Cardiophorus gramineus* **34–35** Sclerotised base of spermathecal gland duct of Cardiophorinae, lateral view Scale bar = 0.5 mm). **34** *Paracardiophorus cardisce* **35** *Paracardiophorus musculus*. SD = spermathecal gland duct. Captions reflect the revised classification.

**Figure 36.**
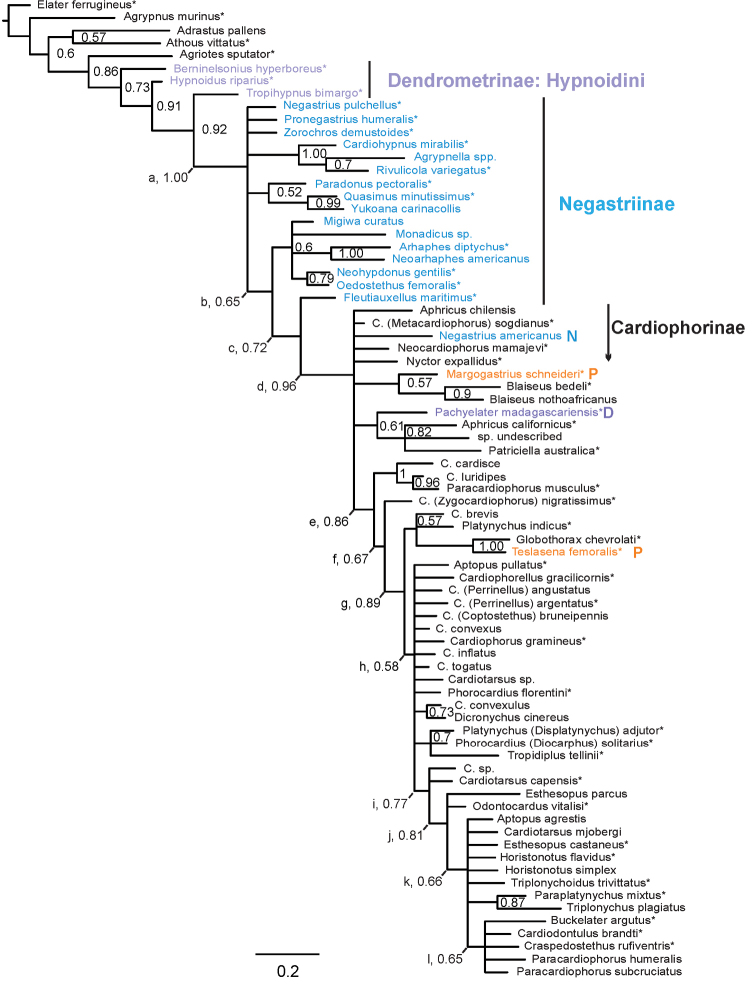
Inferred phylogeny of Cardiophorinae and Negastriinae based on 159 adult morphological characters. Tree is a 50% majority-rule phylogram with branch lengths estimated by MrBayes, of 120000 post-burnin trees. Model = Mkv+G. Values are posterior probabilities. Scale bar indicates 0.1 changes per site. Several nodes are labelled a-l to simplify discussion. Taxon labels D, P, and N are members of Dendrometrinae, Physodactylinae and Negastriinae within the Cardiophorine. Genus name “Cardiophorus” indicates *Cardiophorus*.Names are pre-revision. Type species of genera are marked with “*”.

**Figure 37.**
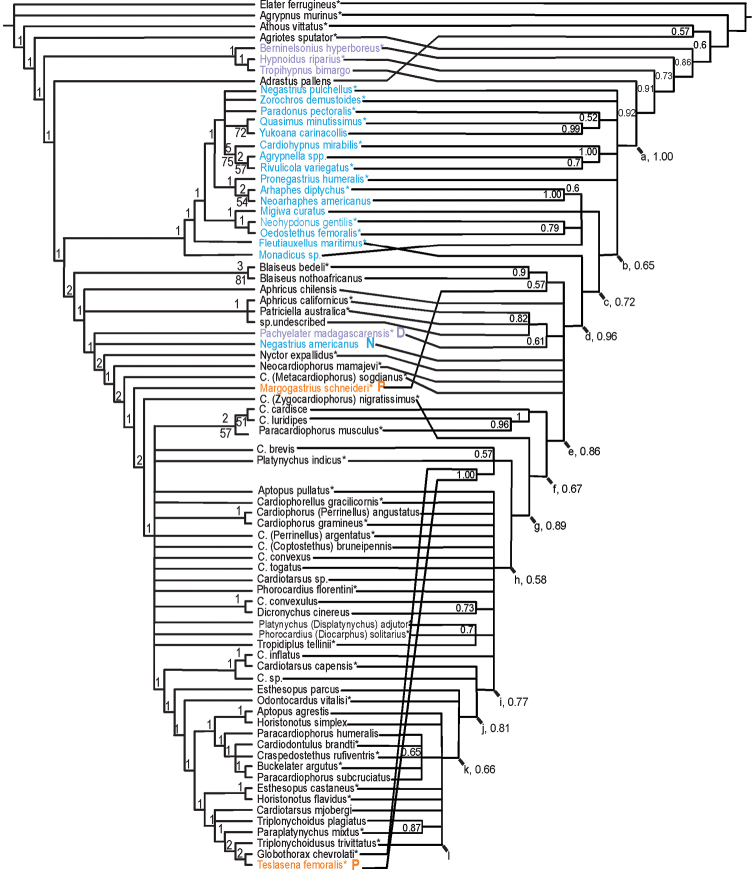
Inferred phylogeny of Cardiophorinae and Negastriinae based on 139 parsimony-informative adult morphological characters (left), with Bayesian topology (adapted from Fig. 36) on right. Parsimony tree is a strict consensus of 412 most parsimonious trees of 1083 steps, CI = 0.17. Values above branches are decay indices, values below branches are bootstrap indices (above 50%). D (and colour purple), P (and colour orange), and N (and colour blue) are members of Dendrometrinae , Physodactylinae and Negastriinae respectively within the Cardiophorine clade (shown by colour alone elsewhere). Genus name “Cardiophorus” indicates *Cardiophorus*. Names are pre-revision. Type species of genera are marked with “*”.

**Figures 38–49.**
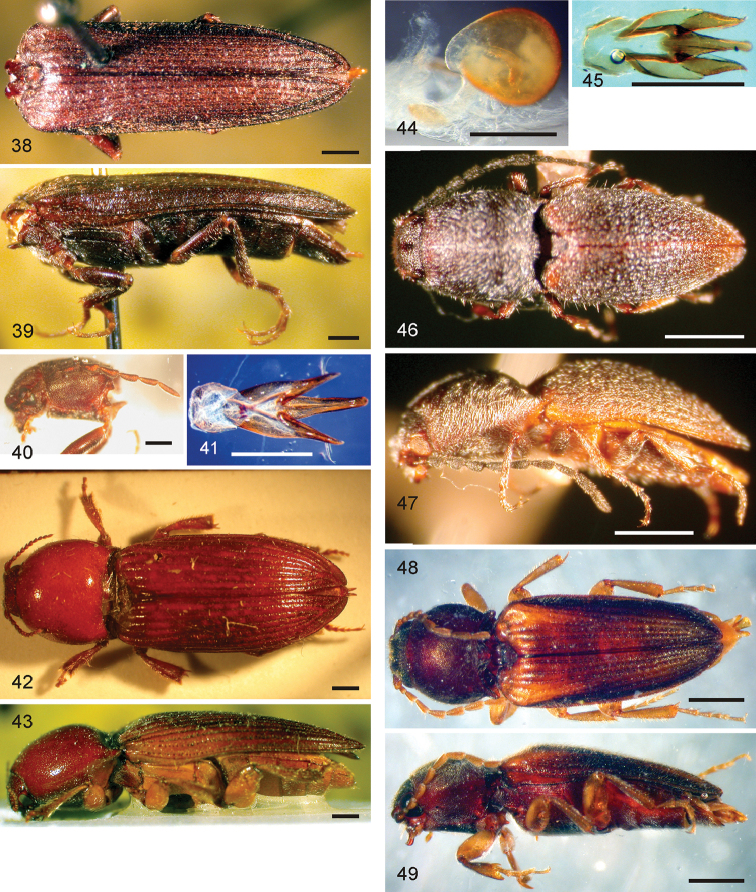
**38–41** *Chileaphricus chilensis* lectotype male **38, 39** pterothorax **40** head and prothorax **41** aedeagus **42–44** *Margogastrius schneideri* paralectotype female. **42, 43** habitus **44** spermatheca, lateral view **45–47** *Floridelater americanus*. **45** aedeagus **46, 47** male **48–49** *Blaiseus bedeli* lectotype male. Scale bars: 1 mm, 0.5 mm for detail photos. Captions reflect the revised classification.

**Figures 50–62.**
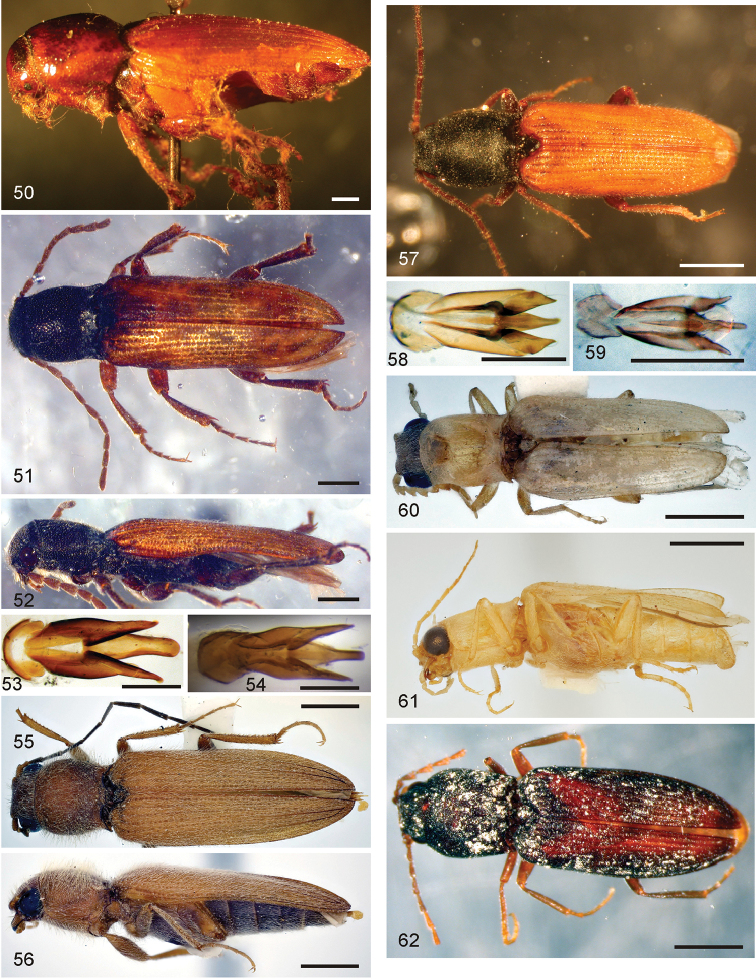
**50–53** *Pachyelater madagascariensis* **50** female **51, 52** male **53** aedeagus **54–56** *Aphricus* sp. **54** aedeagus **55, 56** male **57–58** *Neocardiophorus mamajevi*. **57** male **58** aedeagus **59–61** *Nyctor expallidus*. **59** aedeagus **60, 61** male **62** *Metacardiophorus sogdianus*, male paratype. Scale bars: 1 mm for habiti, 0.5 mm for detail photos. Captions reflect the revised classification.

**Figures 63–78.**
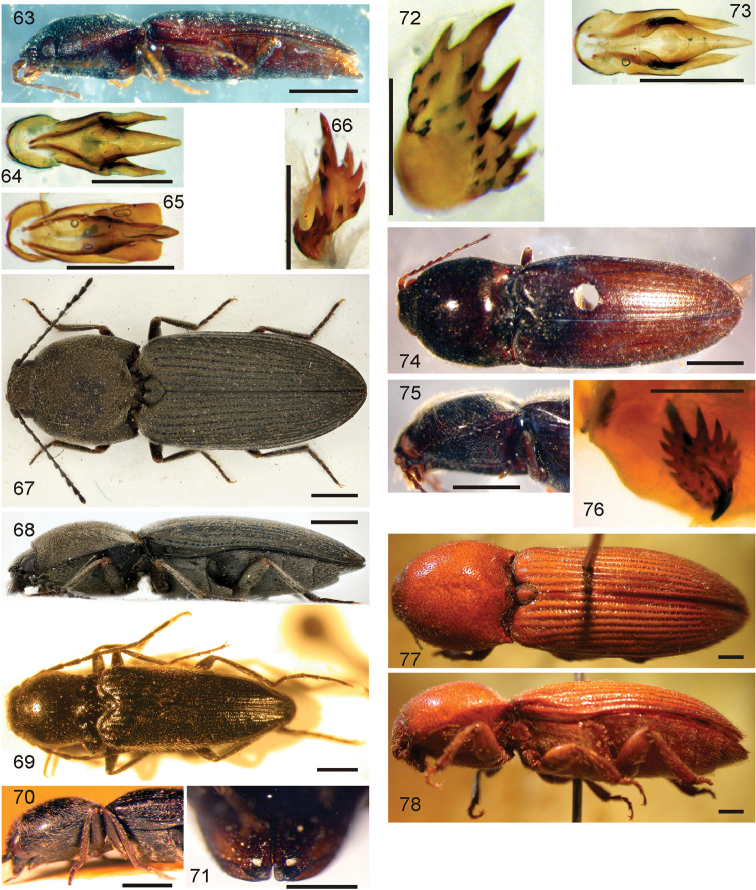
**63–64** *Metacardiophorus sogdianus*, paratype **63** male **64** aedeagus **65–68.** *Paracardiophorus musculus*. **65** aedeagus **66** proximal sclerite **67, 68** female **69–73.** *Zygocardiophorus nigratissimus*. **69, 70** male **71** elytral apex **72** proximal sclerite **73** aedeagus **74–76** *Platynychus indicus*, female lectotype **74–75** adult **76** distal and proximal sclerites of bursa copulatrix, lateral view **77, 78** *Globothorax chevrolati* lectotype female. Scale bars: 1 mm for full habiti, 0.5 mm for detail photos. Captions reflect the revised classification.

**Figures 79–89.**
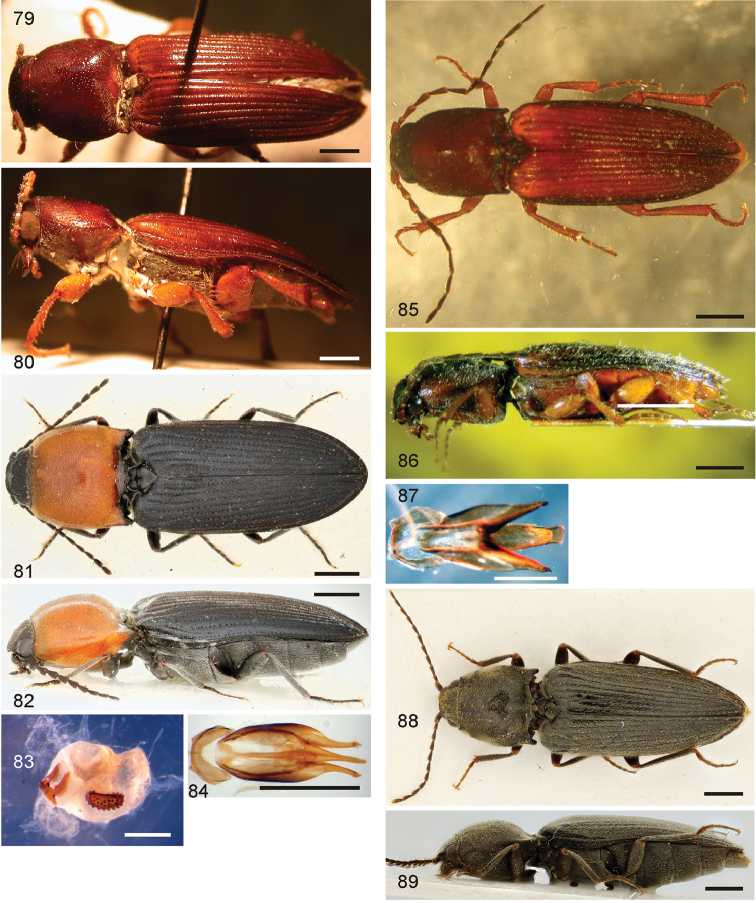
**79, 80** *Globothorax femoralis* lectotype male **81–84** *Cardiophorus gramineus* **81, 82** female **83** bursa copulatrix with distal and proximal sclerites, lateral view **84** aedeagus **85–87** *Cardiophorellus gracilicornis* paratype. **85, 86** male **87** aedeagus **88–89** *Dicronychus cinereus* female. Scale bars: 1 mm for habiti, 0.5 mm for detail photos. Captions reflect the revised classification.

**Figures 90–104.**
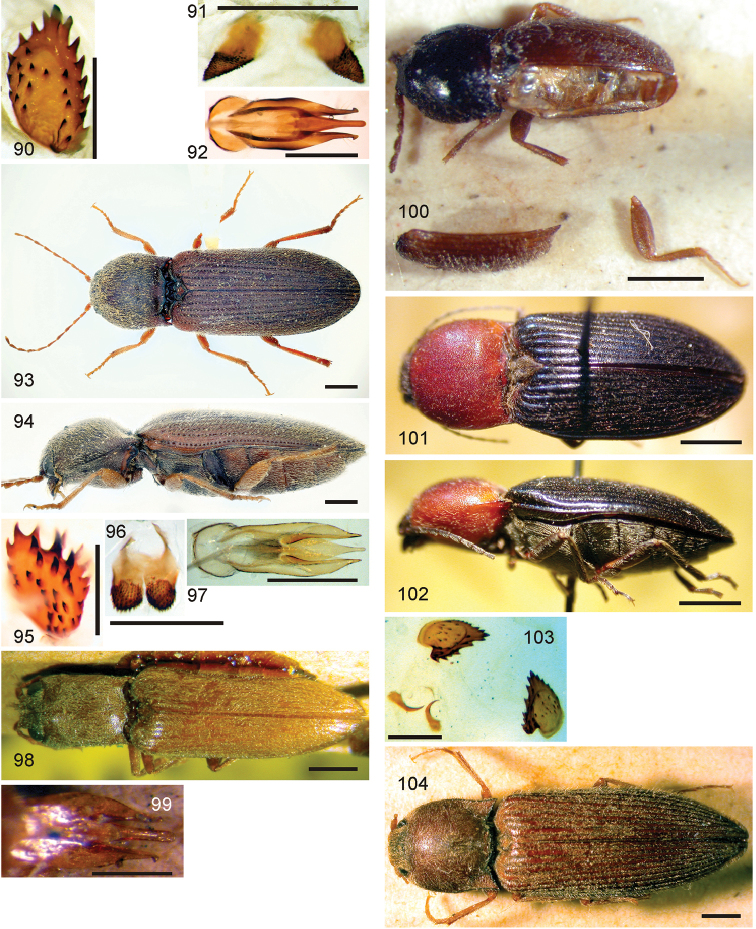
**90–92** *Dicronychus cinereus*. **90** proximal sclerite **91** distal sclerites **92** aedeagus **93–97** *Aptopus rugiceps* **93, 94** adult **95** proximal sclerite **96** distal sclerite **97** aedeagus **98–99** Cardiophorus (Perinellus) argentatus, Lectotype. **98** male **99** aedeagus **100** Cardiophorus (Coptostethus) femoratus, Lectotype **101–103** *Phorocardius florentini* **101, 102** Lectotype **103** bursal sclerites **104** *Tropidiplus tellinii*, female. Scale bars: 1 mm for habiti, 0.5 mm for detail photos. Captions reflect the revised classification.

**Figures 105–121.**
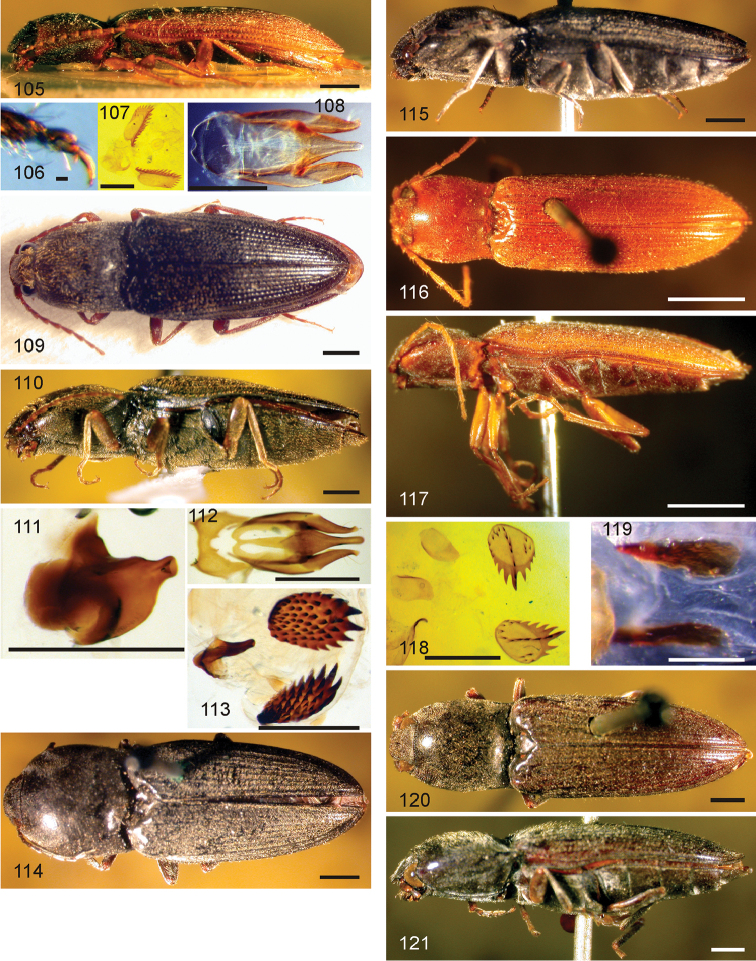
**105–108** *Tropidiplus tellinii* **105** female **106** tarsal claw with basal seta, lateral view (Lectotype male) **107** distal & proximal sclerites, internal view **108** aedeagus (Lectotype male) **109–112** *Displatynychus adjutor* **109, 110** female **111** distal sclerite **112** aedeagus. **113–115** *Diocarphus solitarius*. **113** distal and proximal sclerites in bursa copulatrix, internal view **114, 115** female **116–118** *Cardiotarsus capensis* **116, 117** male **118** bursal sclerites lectotype **119–121** *Odontocardus vitalisi* **119** distal sclerites of bursa copulatrix, internal view **120, 121** male. Scale bars: 1 mm for habiti, 0.5 mm for detail photos, 0.1 mm for tarsal claw. Captions reflect the revised classification.

**Figures 122–139.**
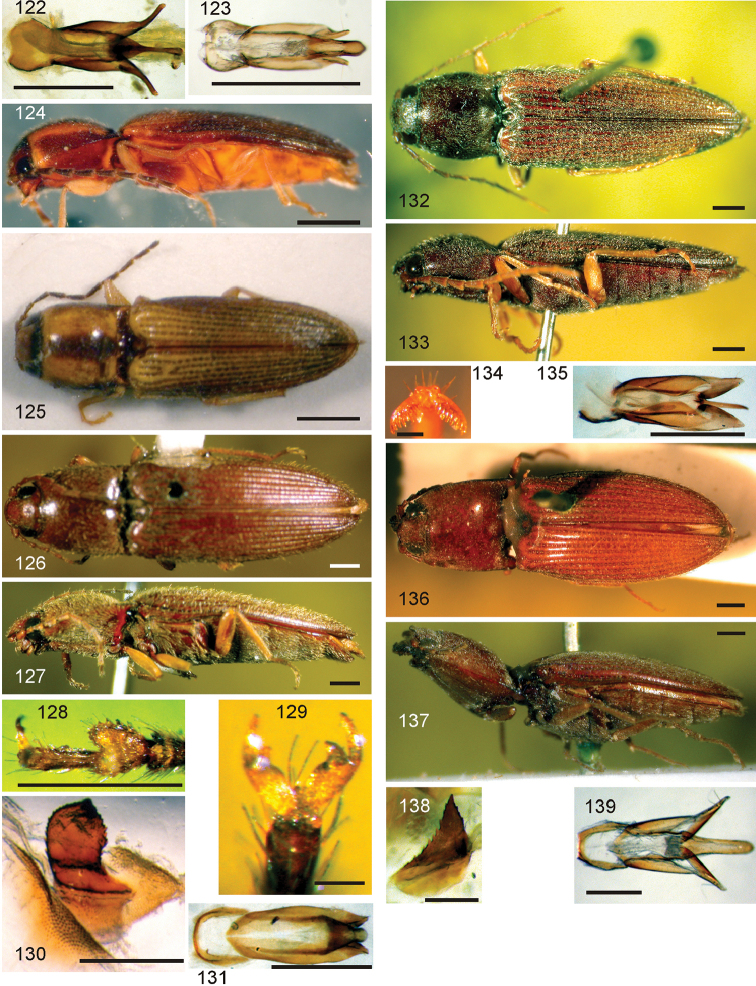
**122** *Odontocardus vitalisi*. Aedeagus **123–125** *Triplonychoidus trivittatus* paralectotype. **123** aedeagus **124, 125** male **126–131** *Esthesopus castaneus* **126, 127** male **128** ventral view of tarsomeres 4&5 **129** tarsal claw **130** proximal sclerite of bursa copulatrix **131** aedeagus **132–135** *Aptopus agrestis* **132, 133** male **134** tarsal claw **135** aedeagus **136–139** *Horistonotus flavidus* **136, 137** lectotype female **138** proximal sclerite of lectotype **139** aedeagus Scale bars: 1 mm for habiti, 0.5 mm for detail photos, 0.1 mm for tarsal claw. Captions reflect the revised classification.

**Figures 140–154.**
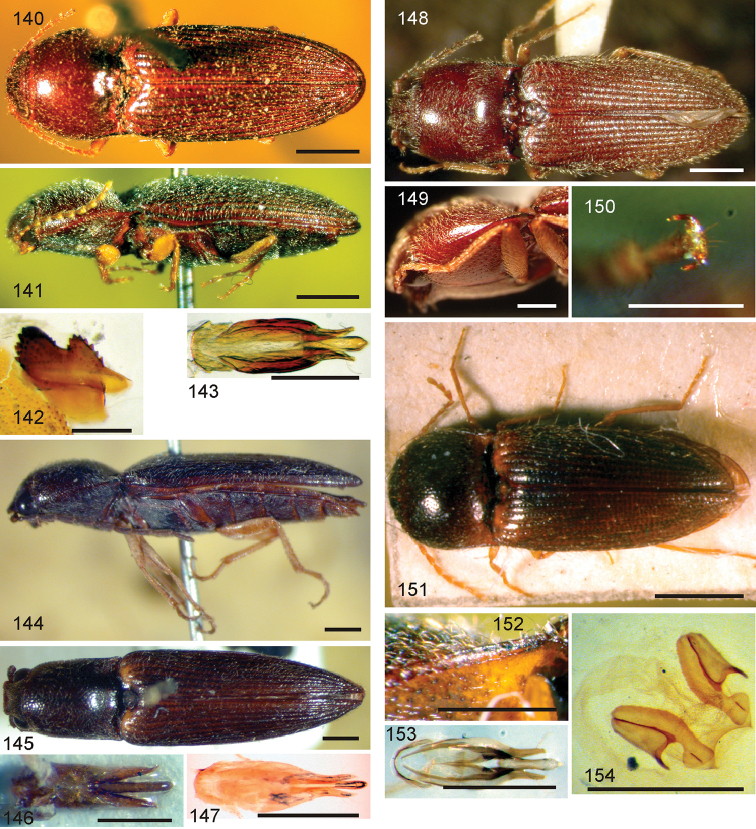
**140–143** *Paraplatynychus mixtus* **140, 141** male **142** proximal sclerite **143** aedeagus **144–146** *Triplonychus acuminatus*, Lectotyp. **144, 145** male **146** aedeagus **147–150** *Cardiodontulus brandti*, male paratype **147** aedeagus **148, 149** adult **150** tarsal claw **151–153** *Craspedostethus rufiventris* **151** female Lectotype **152** lateral view of pronotal hind angle of female Lectotype **153** aedeagus **154** *Craspedostethus culcarius* , bursal sclerites of female labeled as type. Scale bars: 1 mm for habiti, 0.5 mm for detail photos, 0.25 for **142, 143**. Captions reflect the revised classification.

**Figures 155–162.**
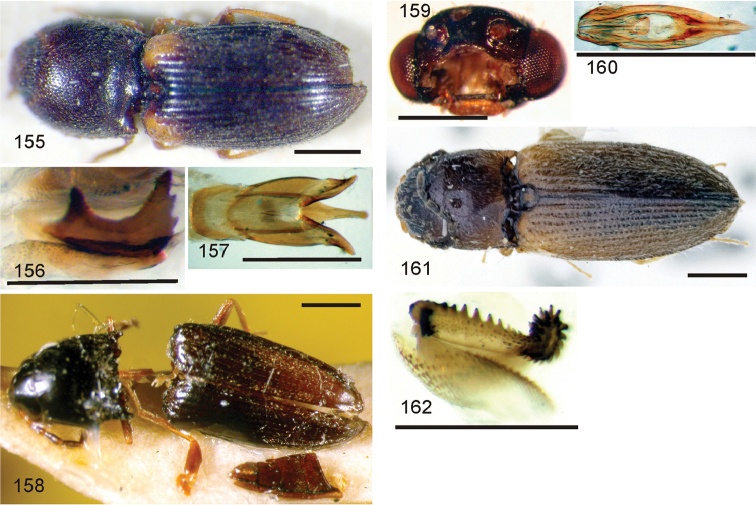
**155–157** *Austrocardiophorus humeralis*. **155** adult **156** proximal sclerite **157** aedeagus **158–160** *Buckelater argutus* **158** male **159** anterior view of head capsule of male **160** aedeagus **161–162** *Ryukyucardiophorus loochooensis* **161** female **162** proximal sclerite. Scale bars: 1 mm for habiti, 0.5 mm for detail photo. Captions reflect the revised classification.

## Revised synonymy of Cardiophorinae

A complete bibliographic synonymy is presented here with references in chronological order to accurately document the nomenclatural history of the group through 2015. All family-group names in synonymy under Cardiophorinae. Several references were unavailable for examination, as is noted in the text. The synonymy began with a draft catalog provided by Prof. Paul Johnson (South Dakota State University, USA) and Schenkling’s most recent (1925) world catalog for all historically and currently recognized cardiophorine names. The *Genera Insectorum* (Schwarz 1906) and the *Biologia Centrali-Americana* (Champion 1895) were also consulted. Since earlier names and applications were sometimes not cited in these works, a general search was made through them to track names and their origins. Following this procedure, the following monographs, reviews, faunal lists and faunal works were also examined (Candèze 1860, 1891a; Gistel 1834, 1848, 1856; Harold 1869; Heyden et al. 1891, 1908; Jakobson 1913; Jacquelin du Val 1859; Kiesenwetter 1858; Lacordaire 1857; Stein and Wiese 1877). In addition, the following lists of genus-group names and the works they cited were also examined (Agassiz 1846; Scudder 1882, Waterhouse 1902, 1912, Lucas 1920).

The following faunal studies and lists were examined to find taxonomic changes within the Cardiophorinae since Schenkling’s 1925 Catalog: *Zoological Record*; Thomson 1864; Blackwelder 1944; Cate 2007; Cobos 1970b; Dajoz 1963; Fleutiaux 1929a, b, 1930, 1931, 1932a, b, c, 1935b, 1947a, b; Golbach 1994; Gurjeva 1966; Leseigneur 1972; Miwa 1934; Neboiss 1956, 1961. Neave (1939) was checked for homonyms of all genus level names.

**Cardiophorinae Candèze, 1859.**Candèze 1859: 4. Type genus: *Cardiophorus* Eschscholtz, 1829. (As Cardiophorites, key only)

—LeConte 1861: 166 (as Cardiophori)

—Thomson 1864: 109 (as Cardiophorina)

—Champion 1895: 415

—Schwarz 1906: 160 (genus level revision, catalog)

—Hyslop 1917: 263 (misspelled as Cardiophorinae)

Aptopina Jakobson, 1913: 760. Type genus: *Aptopus* Eschscholtz, 1829. Synonymy verified by inclusion of Cardiophorina in synonymy under Aptopina.

—Gurjeva 1974a: 111 (as synonym of Cardiophorinae, thoracic characters)

Esthesopinae Fleutiaux, 1919: 76 (incorrectly includes senior synonym, Cardiophorites Candèze, in synonymy). Type genus: *Esthesopus* Eschscholtz, 1829.

—Schenkling: 1925: 218 (as synonym of Cardiophorinae, catalog, distribution)

—Fleutiaux 1932c: 36 (as Esthesopini, tribe of Elaterinae).

Dicronychidae*sensu* Chûjô & Ôhira 1965: 28 (not Fleutiaux). Elevation, in error of DicronychinaeFleutiaux 1919: 101 to family rank [name, based on misapplication of homonym *Dicronychussensu* Laporte (replaced by *Eudicronychus*Méquignon 1931) to Cardiophorinae (not *Dicronychus* Brullé)].

**Cardiophorini Candèze, 1859.**Candèze 1859: 4. Type genus: *Cardiophorus* Eschscholtz, 1829. (As Cardiophorites, key only)

—Candèze 1860: 100

—Champion 1895: 415

—Schwarz 1906: 160 (genus level revision, catalog)

—Heyden et al. 1908: 392 (catalog, tribe includes *Cryptohypnus* and *Hypnoidus*)

—Hyslop 1917: 259 (phylogeny of Elateridae)

—Schenkling 1925: 218 (checklist)

—Miwa 1934: 26 (as synonym of Esthesopinae, spp. of Japanese Empire)

—Stibick 1979a: 181 (classification of Elateridae)

—Gurjeva 1974a: 112 (thoracic characters)

—Dolin 1975: 1632 (wing venation)

—Dolin 1978b: 16 (larvae, USSR)

Nyctorini Semenov-Tian-Shanskij & Pjatakova, 1936: 101. **Syn. n.** Type genus: *Nyctor* Semenov-Tian-Shanskij & Pjatakova, 1936. Described in Elaterinae

—Gurjeva 1974a: 112 (Apparently transferred to Cardiophorinae without comment)

—Dolin 1975: 1632 (as synonym of Cardiophorini)

—Stibick 1979: 181 (as valid without explanation)

### Genera in alphabetical order

***Allocardiophorus*** Ôhira, 1989: 79 (species included: monotypic). Type species: *Paracardiophorus nigroapicalis* Miwa, 1927: 109. Fixed by original designation.

Cate 2007: 194 (catalog, Palaearctic)

***Aphricus*** LeConte, 1853: 501. Type species: *Aphricus californicus* LeConte, 1853: 502. Type species fixed by original monotypy. Described in Cebrionites.

—Lacordaire 1857: 233 (genera of Elateridae, in Campylides)

—Candèze 1863: 471 (species level revision of Elateridae, in Campylides)

—Harold 1869: 1602 (catalog)

—Henshaw 1885: 66 (catalog, in Plastoceridae)

—Candèze 1891a: 217 (catalog, Plastocerites)

—Schwarz 1907: 8 (genus level revision, catalog)

—Woodworth 1913: 197 (checklist, California USA)

—Leng 1920: 166 (catalog)

—Fleutiaux 1940: 103

—Knull 1957: 201 (key to spp.)

—Arnett 1960: 509 (genera of USA, transferred to Cardiophorinae)

—Golbach 1979a: 400 (key to Argentinean genera)

—Golbach 1994: 24 (key to genera of South America)

—Johnson 2002: 168 (genera of USA)

*Patriciella* Van Zwaluwenburg, 1953: 20. **syn. n.** (Replacement name for *Patricia* Van Zwaluwenburg).

—Neboiss 1961: 28 (checklist, Australia)

—Calder 1996: 360 (checklist, Australia, transferred to Cardiophorinae p. 377)

*Patricia* Van Zwaluwenburg, 1947: 113 (species included: monotypic). Type species: *Patricia australica*Van Zwaluwenburg 1947: 114. Type species fixed by original designation. Preoccupied by *Patricia* Fox 1940 (Lepidoptera).

—Neboiss 1956: 64 (checklist, Australia)

***Aptopus*** Eschscholtz, 1829: 32 (species included: *ephippiger*, *tibialis*). Type species: *Aptopus tibialis* Eschscholtz, 1829: 32. Type species fixed by Hyslop 1921: 629. Definition restricted here to exclude species near *Aptopus agrestis* (Erichson).

—Dejean 1836: 99 (catalog)

—Laporte 1840: 251 (as synonym of junior name *Priopus* Laporte, 1840)

—Lacordaire 1857: 193 (as synonym of *Cardiophorus*)

—Candèze 1860: 230 (species level revision of Elateridae)

—Harold 1869: 1553 (catalog)

—Horn 1884: 45 (sp.n., U.S.A)

—Henshaw 1885: 66 (catalog)

—Candèze 1889: 111 (spp.n.)

—Candèze 1891a: 134 (catalog)

—Candèze 1891b: 781 (sp.n., Burma)

—Champion 1895: 418 (spp. of Central America)

—Schwarz 1906: 175 (genus level revision, catalog. including as misspelled as *Apsopus* (p. 180). Misspelling verified by inclusion of the correct reference page for *Aptopus* Eschscholtz)

—Schaeffer 1916 (spp.n.)

—Leng 1920: 175 (catalog)

—Schenkling: 1925: 254 (catalog, distribution)

—Blackwelder 1944: 302 (checklist)

—Arnett 1960: 508 (genera of USA)

—Dolin 1975: 1632 (wing venation)

—Golbach 1979a: 400 (key to Argentinean genera)

—Golbach 1994: 24 (key to genera of South America)

—Aranda 1996: 444 (key to spp. of Argentina)

—Aranda 1998: 130 (genus descr. from S. American spp.)

—Johnson 2002: 168 (genera of USA)

—Aranda 2003: 139 (spp.n.)

—Aranda 2005 (spp. n., key to species of Argentina)

—Aranda 2008 (spp. n. South America)

*Eniconyx* Horn, 1884: 51. Type species: *Eniconyx pullatus* Horn, 1884: 52 Type species fixed by Hyslop 1921: 644.

—Henshaw 1885: 66 (catalog)

—Candèze 1891a: 217 (catalog, in Plastoceridae, misspelled as *Enisonyx*. Misspelling verified by reference to Horn publication and spp. epithets belonging to *Eniconyx*.)

—Waterhouse 1902: 123

—Schwarz 1907: 7 (genus level revision, catalog, misspelled as *Enisonyx*)

—Leng 1920: 166 (catalog, in Plastoceridae)

—Arnett 1960: 508 (as synonym of *Aptopus*)

***Austrocardiophorus* gen. n.** Type species *Cardiophorus humeralis* Fairmaire & Germain, 1860: 5.

***Blaiseus*** Fleutiaux, 1931: 307. Type species: *Blaiseus bedeli* Fleutiaux, 1931: 308. Type species fixed by original monotypy.

—Fleutiaux 1947a: 343 (spp. of French Indochina)

—Gurjeva 1974a: 112 (misspelled as *Blaiseus*, verified by placement in context of other cardiophorine genera)

—Cate 2007: 194 (catalog, Palaearctic)

—Douglas 2009: 86 (spp. of world)

***Buckelater*** Costa, 1973: 33. Type species: *Buckelater argutus* Costa, 1973: 35 (species included: monotypic). Type species fixed by original designation.

—Golbach 1979a: 399 (key to Argentinean genera)

—Golbach 1994: 24 (key to genera of South America)

—Aranda and Cordoba 2007: 3 (male, genus diagnosis)

*Caloderus* Stephens, 1830, see: *Cardiophorus* Eschscholtz

***Cardiodontulus*** Van Zwaluwenburg, 1963: 341. Type species: *Cardiodontulus brandti* Van Zwaluwenburg, 1963: 341 (species included: monotypic). Type species fixed by original designation.

—*Cardiotarsus mjobergi* Carter **is here transferred** to this genus as *Cardiodontulus mjobergi* (Carter, 1939)

***Cardiophorellus*** Cobos, 1970a: 222. Type species: *Cardiophorellus gracilicornis*Cobos 1970a: 223. Type species fixed by original monotypy of nominate subgenus

***Cardiophorellus*: subgenus Parapleonomus** Cobos, 1970a: 222 (species included: monotypic). Type species: *Cardiophorellus inermis* Cobos, 1970a: 222. Type species fixed by original designation.

***Cardiophorus*** Eschscholtz, 1829: 34 (species included: *biguttatus*, *charactericus*, *discicollis*, *ebininus*, *equisiti*, *exaratus*, *latiusculus*, *luzonicus*, *ornatus*, *ruficollis*, *rufipes*, *suturalis*, *thoracicus*). Type species: *Elater thoracicus*Fabricius 1801: 236, now regarded as a synonym of *Cardiophorus gramineus* (Scopoli, 1763). Type species fixed by Westwood 1838: 26.

—Dejean 1833: 91 (catalog)

—Dejean 1836: 103 (catalog)

—Eschscholtz 1836: Table

—Germar 1839: 196 (key to genera of Elateridae)

—Erichson 1840: (species level revision)

—Redtenbacher 1849: 300 (spp. of Austria)

—LeConte 1853: 496 (spp. of USA, also includes *Horistonotus* spp.)

—Lacordaire 1857: 193 (genera of Elateridae)

—Redtenbacher 1858: 495 (spp. of Austria)

—Motschulsky 1858: 59 (misspelled as *Cardiaphorus*. Verified as *Cardiophorus* ref. by mention of *Cardiophorus curiatus*)

—Jacquelin du Val 1859: 141 (genera of Europe)

—Thomson 1859: 104 (genera of Scandinavia)

—Candèze 1860: 106 (species level revision of Elateridae)

—Thomson 1864: 116 (spp. of Scandinavia)

—Harold 1869: 1546 (catalog)

—Candèze 1875: 16 (spp.n.)

—Horn 1871: 301 (spp.n., U.S.A)

—Redtenbacher 1874: 537 (spp. of Austria)

—Henshaw 1885: 66 (catalog)

—Candèze 1889: 105 (spp.n.)

—Candèze 1891a: 121 (catalog)

—Candèze 1891b: 778 (spp.n.)

—Heyden et al. 1891: 205 (catalog)

—Candèze 1892: 805 (sp.nov)

—Champion 1895: 416 (spp. of Central America)

—Candèze 1897: 55 (spp.n.)

—Jäger 1897: 356 (redescription, biology)

—Blanchard 1889: 1 (spp. of N. America)

—Buysson 1891: 134 (note)

—Candèze 1893: 45 (spp.n.)

—Fleutiaux 1895a: 687 (sp.nov)

—Schwarz 1895a: 37 (sp.nov)

—Schwarz 1895b: 40 (key to genera)

—Schwarz 1896: 148 (sp.nov)

—Buysson 1899: 279 (note)

—Candèze 1900: 93 (sp.n.)

—Buysson 1902: 286 (spp. of France and Rhine Valley)

—Schwarz 1902: 259 (sp.n.)

—Schwarz 1903c: 370 (spp.n.)

—Schwarz 1905: 289 (spp.n.)

—Schwarz 1906: 162 (genus level revision, catalog)

—Heyden et al. 1908: 393 (catalog, Europe)

—Buysson 1910: 138 (note)

—Buysson 1911: 22 (spp. of Egypt)

—Buysson 1912: 128 (spp.n., Russia)

—Jakobson 1913: 761 (key, catalog, Europe)

—Woodworth 1913: 197 (checklist, California USA)

—Buysson 1914: 41 (sp.n.)

—Fleutiaux 1918b: 225 (spp.n., Indochina)

—Fleutiaux 1919: 76 (spp.n., east Africa)

—Leng 1920: 175 (catalog)

—Fleutiaux 1921: 14 (spp. record, English Africa)

—Buysson 1924: 207 (spp. of Morocco)

—Cockerell 1925: 9 (sp.n., Eocene fossil)

—Schenkling 1925: 219 (catalog, distribution)

—Méquignon 1931: 207 (note)

—Miwa 1927: 106 (key to genera of Japanese Empire)

—Miwa 1930: 1 (key to genera et spp. of Formosa)

—Miwa 1934: 26 (spp. of Japanese Empire)

—Fleutiaux 1931: 313 (catalog, French Indochina)

—Fleutiaux 1932b: 20 (spp. of Mozambique)

—Carter 1939: 310 (spp. of Australia transferred to *Paracardiophorus*)

—Jagemann 1940: 55 (spp. Europe)

—Normand 1941: 331 (sp.n., N. Africa)

—Blackwelder 1944: 301 (checklist)

—Van Zwaluwenburg 1947: 111 (spp. of Australia)

—Fleutiaux 1947a: 344 (spp. of French Indochina)

—Cobos 1954: 86 (checklist, Sierra Nevada, Spain. Misspelled as *Cardiophorua*. Misspelling verified by cardiophorine context)

—Binaghi 1955: 4 (note)

—Jagemann 1955: 136 (spp. of Czechoslovakia)

—Neboiss 1956: 39 (transferred all Australian spp. to *Paracardiophorus*, checklist, Australia)

—Tsherepanov 1957: 234 (spp. of W. Siberia)

—Cobos 1959: 468 (sp.n., Brazil)

—Arnett 1960: 508 (genera of USA)

—Cobos 1961: 63 (spp. of Morocco)

—Ôhira 1962: 177 (higher classification, larvae)

—Chûjô and Ôhira 1965: 28 (spp. of Japan)

—Dajoz 1963: 165 (spp. of Europe and Mediterranean region)

—Gurjeva 1966: 64 (spp. of central Asia)

—Leiler 1967: 89 (larvae and pupae, Europe)

—Gurjeva 1969: 156 (larval evolution)

—Cobos 1970a: 232 (spp.n., Congo)

—Cobos 1970b: 29 (spp. of Canary Islands)

—Ôhira 1970: 216 (types from Southeast Asia)

—Ôhira 1971: 212 (types from South Asia)

—Lanchester 1971: 35 (spp. of northwestern USA)

—Leseigneur 1972: 130 (spp. of France)

—Laurent 1974: 22 (spp. of South Africa)

—Gurjeva 1974a: 111 (thoracic characters)

—Gurjeva 1974b: 170 (spp.nov, Afghanistan. Misspelled as *Cardiosphorus* in English translation only)

—Dolin 1975: 1632 (wing venation)

—Dolin 1978a: 8 (phylogeny of Elateridae)

—Dolin 1978b: 73 (larvae, USSR)

—Chassain 1979: 194 (spp. of Saudi Arabia)

—Golbach 1979a: 400 (key to Argentinean genera)

—Lohse 1979: 181 (spp. of central Europe)

—Iablokoff-Khnzorian and Mardjanian 1981: 247 (subgen. n.)

—Dolin 1988: 7 (spp. of Ukraine)

—Bousquet 1991: 185 (catalog)

—Kompantseva 1991: 18 (key: larvae of Tajikistan)

—Vats and Chauhan 1991: 11 (spp. of North India)

—Atamuradov 1993: 118 (larvae, central Asia)

—Downie and Arnett 1996: 821 (spp. of northeastern North America)

—Platia and Gudenzi 2000a: 601, 2002: 28 (spp. of Turkey)

—Platia and Gudenzi 2000b: 136 (spp. of Greece);

—Wurst et al. 2001: 547 (spp.n., Yemen)

—Johnson 2002: 168 (genera of USA)

—Cate et al. 2002: 46 (spp.n., Iran)

—Platia et al. 2002: 76 (sp.n., Iran)

—Douglas 2003: 493 (spp. of eastern North America)

—Lucht 2005: 198, 199 (distribution, bionomics)

—Platia and Baviera 2005: 179 (sp.n., Sicily)

—Platia and Gudenzi 2007: 91 (sp.n., Syria)

—Platia 2008a (sp.n., United Arab Emirates)

—Platia 2008b (sp.n., Balearic Islands)

—Platia 2008c: 203 (spp.n., Iran, Tajikistan, Kuwait, sp. removed from synonymy)

—Hawkswood et al. 2009 (sp.n., Madagascar fossil)

—Platia and Gudenzi 2009: 121 (spp.nov, Turkey, Russia)

—Mardjanian and Varandi 2011: 396 (spp.nov, Iran)

—Platia 2010a: 128 (spp.n., Iran)

—Platia 2010b: 36 (spp.n., Turkey, Iran, Jordan, Iraq, Israel)

—Akhter et al. 2011: 478 (sp.n., key to species of Pakistan)

—Platia 2011: 48 (sp.n., Turkey)

—Platia et al. 2011: 214 (spp.n. Turkey)

—Platia 2012a: 203

—Platia 2012b: 136 (spp.n. Yemen)

—Platia 2013: 100 (sp.n. Greece)

—Zapata and Sanchez-Ruiz 2013: 78 (spp.n. Spain)

—Nemeth and Platia 2014: 466 (spp.n., syn. n. Turkey, Afghanistan, Armenia)

—Platia 2014: 72 (sp.n. Yemen)

—Platia and Kakiopoulos 2015: 46 (spp.n. Greece, Turkey)

*Caloderus* Stephens, 1830: 269 (species included: *equisiti*, *ruficollis*, *thoracicus*). Type species: *Caloderus thoracicus* Fabricius, 1801: 236. Type species fixed by Hyslop 1921: 632.

—Laporte 1840: 249 (with *Cardiophorus* in synonymy)

—Westwood 1838: 26 (genera of British Insects, synonymized under *Cardiophorus*)

—Lacordaire 1857: 138 (genera of Elateridae, as synonym of *Cardiophorus*)

—Candèze 1860: 106 (species level revision of Elateridae, as synonym of *Cardiophorus*)

*Paradicronychus* Dolin & Gurjeva, 1975: 116 (*nomen nudum*, species included *inflatus*, *nothus*). No type species designated for this name, published after 1930, therefore name is not available.

—Dolin 1978b: 81 (larvae, USSR)

—Platia 1994: 380 (spp. of Italy, as synonym of *Dicronychus* without comment)

—Cate 2007: 207 (catalog, Palaearctic, as synonym of *Platynychus* without comment)

—**Syn. n.** of *Cardiophorus* here.

***Cardiophorus*: subgenus Coptostethus** Wollaston, 1854: 238. Rank lowered to subgenus by Cobos 1970b: 34. Subgenus rank recognised by: Cobos 1978: 145 (spp. Atlantic Islands)

—Johnson 2001: 58 (Coptostethus (Coptostethus) americanus transferred to *Negastrius*)

—Cate 2007: 202 (catalog, Palaearctic) [see also *Floridelater*].

*Coptostethus* Wollaston, 1854: 238. Type species: *Cardiophorus femoratus* Wollaston, 1854: 240. Type species fixed by original monotypy.

—Lacordaire 1857: 196 (genera of Elateridae)

—Candèze 1860: 104 (misspelled as *Coptosthetus*. Verified by listing of correct spp.)

—Harold 1869: 1546 (catalog)

—Horn 1871: 303 (sp.n., U.S.A)

—Henshaw 1885: 66 (catalog)

—Candèze 1889: 105 (spp.n.)

—Fleutiaux 1891 (comparison to *Globothorax*)

—Candèze 1891a: 121 (catalog)

—Schwarz 1906: 161 (genus level revision, catalog)

—Jakobson 1913: 761 (key, catalog, Europe)

—Leng 1920: 175 (sp. of N. America)

—Schenkling: 1925: 218 (catalog, distribution)

—Blackwelder 1944: 301 (checklist)

—Arnett 1960: 508 (genera of USA)

—Dajoz 1963: 173 (spp. of Europe and Mediterranean region)

—Cobos 1970b: 29 (spp. of Spain: Canary Islands)

—Laurent 1974: 22 (spp. of South Africa)

—Gurjeva 1974a: 112 (thoracic characters)

—Cobos 1978: 1 (sp.n. Portugal: Madeira Islands)

—Golbach 1979a: 400 (without comment, key to Argentinean genera)

—Cobos 1983: 237 (spp.nov Spain: Canary Islands)

—Golbach 1994: 24 (key to genera of South America)

—Aranda 1998: 130 (key to genera, misspelled as *Coptosthetus* on p. 124 only)

—Schimmel 2008: 351 (new combination., spp.nov, Selvage Islands)

—Nemeth and Platia 2014: 481 (spp.n. Spain: Canary Islands)

—Platia and Kakiopoulos 2015: 47 (sp.n. Spain: Canary Islands)

***Cardiophorus*: subgenus Perrinellus** Buysson, 1899: 282 (species included: *argentatus*, *bonnairei*, *bousaadensis*). Type species: *Athous argentatus*Abeille de Perrin 1894: 92 (= *Cardiophorus reitteri* Schwarz). Type species fixed by original designation.

—Waterhouse 1902: 279 (at generic rank)

—Schwarz 1906: 172 (genus level revision, catalog, as subgenus of *Cardiophorus*. Misspelled as *Perinellus* on p. 172. Verified by corroboration: correct species listed)

—Heyden et al. 1908: 394 (catalog Europe, as subgenus of *Cardiophorus*)

—Buysson 1910: 145 (note, at generic rank)

—Buysson 1911: 22 (spp. of Egypt, at generic rank)

—Schenkling: 1925: 220 (catalog, distribution, as subgenus of *Cardiophorus*)

—Jagemann 1940: 55 (spp. of Europe, as subgenus of *Cardiophorus*)

—Cobos 1950: 155 (note, as subgenus of *Cardiophorus*)

—Cobos 1954: 86 (Faunal list, Sierra Nevada, Spain, as subgenus of *Cardiophorus*)

—Dajoz 1963: 165 (spp. of Europe and Mediterranean region, *Perinellus* not recognised as a natural group)

—Gurjeva 1966: 88 (spp. of central Asia, as subgenus of *Cardiophorus*)

—Platia 1994: 354 (spp. of Italy, as subgenus of *Cardiophorus*)

—Wurst et al. 2001: 548 (spp.n., Yemen, as subgenus of *Cardiophorus*)

—Cate 2007: 202 (catalog, Palaearctic, as subgenus of *Cardiophorus*)

***Cardiophorus*: subgenus Lasiocerus** Buysson, 1912: 129. Type species: *Cardiophorus schusteri*Buysson 1912: 128. Type species fixed by original monotypy. Described at genus rank.

—Schenkling: 1925: 237 (catalog, distribution, as subgenus of *Cardiophorus*)

—Dajoz 1963: 165 (spp. of Europe and Mediterranean region, no status assigned)

—Dolin 1988: 10 (spp. of Ukraine, as synonym of *Cardiophorus* without comment)

—Cate 2007: 194 (catalog, Palaearctic, as synonym of *Cardiophorus*)

—Platia 1994: 354 (spp. of Italy, without comment, as subgenus of *Cardiophorus*)

***Cardiotarsus*** Eschscholtz, 1836: published in identification table opposite p.5, without associated spp. Type species: *Cardiotarsus capensis* Candèze, 1860: 226. Type species fixed by Hyslop 1921: 633.

—Dejean 1833: 91 (proposed name as *nomen nudum*: indication not valid because associated species name was unavailable, catalog)

—Dejean 1836: 103 (catalog)

—Germar 1839: 198 (key to genera of Elateridae)

—Lacordaire 1857: 192 (genera of Elateridae)

—Candèze 1860: 225 (species level revision of Elateridae)

—Harold 1869: 1552 (catalog)

—Candèze 1889: 111 (sp.n.)

—Candèze 1891a: 133 (catalog)

—Candèze 1891b: 780 (sp.n.)

—Candèze 1897: 55 (spp.n.)

—Schwarz 1902: 261 (sp.nov)

—Schwarz 1905: 289 (sp.n.)

—Schwarz 1906: 174 (genus level revision, catalog)

—Fleutiaux 1918b: 231 (sp.n., Indochina, now *Odontocardus*)

—Schenkling: 1925: 253 (catalog, distribution)

—Fleutiaux 1919: 97 (sp. record, east Africa)

—Miwa 1927: 105 (key to genera of Japanese Empire)

—Miwa 1930: 4 (key to genera et spp. of Formosa)

—Fleutiaux 1931: 328 (catalog French Indochina)

—Fleutiaux 1932c: 36 (spp. of Mascarene Is.)

—Miwa 1934: 27 (spp. of Japanese Empire)

—Carter 1939: 309 (spp. of Australia)

—Fleutiaux 1947a: 369 (spp. of French Indochina)

—Neboiss 1956: 46 (checklist, Australia)

—Cobos 1970a: 223 (key to genera, Congo)

—Ôhira 1970: 216 (types from Southeast Asia)

—Laurent 1974: 34 (spp. of South Africa)

—Gurjeva 1974a: 112 (thoracic characters)

—Dolin 1975: 1632 (wing venation)

—Kishii 1992: 8 (sp.n., Taiwan)

—Calder 1996: 360 (checklist, Australia)

—Cate 2007: 203 (catalog, Palaearctic)

—*Cardiotarsus mjobergi* Carter is here recommended for transfer to *Cardiodontulus* Van Zwaluwenburg.

***Chileaphricus* gen. n.** Type species, *Aphricus chilensis* Fleutiaux, 1940: 103 (species included: monotypic).

*Coptostethus* Wollaston, 1854, see: *Cardiophorus*: subgenus Coptostethus Wollaston

*Craspedonotus* Schwarz, 1898, see: *Craspedostethus* Schwarz

***Craspedostethus*** Schwarz, 1898b: 414. Replacement name for *Craspedonotus* Schwarz, 1898.

—Waterhouse 1902: 88

—Schenkling: 1925: 252 (catalog, distribution)

—Dajoz 1963: 172 (spp. of Europe and Mediterranean region)

—Cobos 1970a: 227 (as subgenus of *Paracardiophorus*, spp.n., Congo)

—Chassain 1979: 206 (rejecting placement in *Paracardiophorus* by Cobos 1970a based on sclerites of bursa copulatrix, spp. of Saudi Arabia)

—Wurst et al. 2001: 550 (sp.n., Yemen)

—Cate et al. 2002: 52 (sp.n., Iran)

—Cate 2007: 203 (catalog, Palaearctic)

—Platia 2010b: 40 (sp.n., Israel)

—Platia 2012a: 203 (spp.n., Oman)

—Platia 2012b: 144 (sp.n. Yemen)

—Nemeth and Platia 2014: 483 (spp.n. Iran, Afghanistan)

—Platia 2015a: 25 (sp.n. Pakistan)

*Craspedonotus* Schwarz, 1898a: 148 (species included: *rufiventris*, *semirufus*, *minutissimus*). Type species: *Craspedostethus rufiventris* Schwarz, 1898a: 148. Type species fixed by Hyslop 1921: 636. Preoccupied by *Craspedonotus* Schaum, 1863 (Carabidae)

***Dicronychus*** Brullé, 1832: 138 (species included: *obesus*, *messenicus*). Type species: *Elater obesus* Brullé, 1832: 138, now referred to by replacement name *Dicronychus brullei* Platia & Gudenzi, 2003. Type species fixed by Méquignon 1931: 208. Originally described *Dicronychus* as a subgroup of *Elater* (and considered Eschscholtz the author).

—Eschscholtz 1836: Table (at genus rank)

—Lacordaire 1857: 138 (as synonym of *Cardiophorus*, genera of Elateridae)

—Candèze 1860: 106 (as synonym of *Cardiophorus*, species level revision of Elateridae)

—Harold 1869: 1546 (as synonym of *Cardiophorus*, catalog)

—Candèze 1891a: 121 (as synonym of *Cardiophorus*, catalog)

—Heyden et al. 1891: 205 (as synonym of *Cardiophorus*, catalog)

—Champion 1895: 416 (as synonym of *Cardiophorus*, spp. of Central America)

—Schwarz 1897: 9 (note)

—Schwarz 1903c: 365 (sp.n.)

—Schwarz 1905: 282 (sp.n.)

—Schwarz 1906: 162 (as synonym of *Cardiophorus*, genus level revision, catalog)

—Schenkling: 1925: 220 (as synonym of *Cardiophorus*, catalog, distribution)

— Méquignon 1931: 207 (as subgenus of *Cardiophorus* and senior synonym of subgenus Platynychus)

—Fleutiaux 1932a: 170 “Gen *Dicronychus* Brullé, 1832”

—Fleutiaux 1947a: 348 (spp. of French Indochina)

—Arnett 1955: 608 (note on types)

—Binaghi 1955: 4 (as subgenus of *Cardiophorus*)

—Ôhira 1962: 177 (higher classification, larvae)

—Ôhira 1970: 230 (types from Southeast Asia)

—Ôhira 1971: 212 (types from South Asia)

—Leseigneur 1972: 151 (spp. of France)

—Ôhira 1973b: 38 (spp. of Ceylon)

—Laurent 1974: 23 (spp. of South Africa)

—Gurjeva 1974a: 112 (thoracic characters)

—Dolin 1975: 1621 (as synonym of *Cardiophorus*, wing venation)

—Dolin and Gurjeva 1975: 118 (as synonym of *Cardiophorus*, note on larvae)

—Lohse 1979: 184 (spp. of central Europe)

—Iablokoff-Khnzorian and Mardjanian 1981: 247 (subgen. n.)

—Dolin 1988: 10 (as synonym of *Cardiophorus*, spp. of Ukraine)

—Vats and Chauhan 1991: 11 (as synonym of *Cardiophorus*, no formal synonymy, but includes *Cardiophorus* spp. with diagnostic characters of *Dicronychus*, spp. of North India,)

—Platia 1994: 381 (spp. of Italy)

—Lawrence and Newton 1995: 855 (as subgenus of *Cardiophorus* and senior synonym of subgenus Platynychus)

—Cate et al. 2002: 55 (sp.n., Iran)

—Platia and Gudenzi 2003: 27 (spp. of Greece)

—Platia and Gudenzi 2004: 9 (spp. of Turkey)

—Lucht 2005: 198, 199 (distribution, bionomics)

—Cate 2007: 203 (catalog, Palaearctic)

—Platia and Gudenzi 2007: 91 (sp.n., Syria)

—Platia 2010b: 39 (spp.n., Oman, Syria)

—Platia 2011: 48 (sp.n., Syria)

—Platia et al. 2011: 214 (sp.n. Turkey)

—Al Dhafer and Platia 2013: 19 (spp.n. Saudi Arabia)

—Platia 2012b: 142 (spp.n. Yemen)

—Platia and Akrawi 2013: 72 (sp.n. Iraq)

—Nemeth and Platia 2014: 483 (spp.n. Iran, Afghanistan, Turkey, Oman)

—Platia 2014: 72 (sp.n. Yemen)

—Platia 2015a: 23 (sp.n. Pakistan)

—Platia 2015b: 284 (sp.n. Iran)

—Platia and Kakiopoulos 2015: 47 (spp.n. Egypt, Greece, Turkey, Iran)

*Eudicronychus* Méquignon *sensu* Chûjô & Ôhira, 1965: 28. Error verified by inclusion of spp. assigned to *Dicronychus* Brullé.

*Gauroderus* Thomson, 1859: 104 (species included: monotypic). Type species: *Elater cinereus* Herbst, 1784: 114. Type species fixed by original designation.

—Harold 1869: 1546 (as synonym of *Cardiophorus* with *Dicronychus*, catalog)

—Candèze 1891a: 121 (as synonym of *Cardiophorus*, catalog)

—Champion 1895: 416 (as synonym of *Cardiophorus*, spp. of Central America)

—Schwarz 1906: 162 (as synonym of *Cardiophorus*, genus level revision, catalog)

—Heyden et al. 1908: 394 (as junior synonym of *Platynychus*, as subgenus of *Cardiophorus*, catalog Europe)

—Schenkling: 1925: 220 (as synonym of *Cardiophorus*, catalog, distribution)

—Méquignon 1931: 207 (as objective junior generic synonym of *Dicronychus* because type species is a subjective synonym of the type of *Dicronychus*. Treated as subgenus of *Cardiophorus*)

—Miwa 1934: 26 (as synonym of *Dicronychus*, spp. of Japanese Empire)

—Fleutiaux 1947a: 348 (as synonym of *Dicronychus*, spp. of French Indochina)

—Leseigneur 1972: 151 (as synonym of *Dicronychus*)

—Platia 1994: 380 (as synonym of *Dicronychus*)

*Paramecus* Dillwyn, 1829: 32. Type species: *Paramecus cordiger* Dillwyn, 1829 (= *Elater equiseti* Herbst, 1784: 114). Type species fixed by original monotypy. Objective junior generic synonym of *Dicronychus*.

—Tomlin 1914: 17 (as synonym of *Dicronychus*. This paper subjectively synonymised type species under *D. Equiseti* (Herbst))

—Cate 2007: 204 (as synonym of *Dicronychus*)

***Diocarphus*** Fleutiaux, 1947a: 364. **stat. n.**, raised to genus rank. *Phorocardius*: subgenus Diocarphus Fleutiaux, 1947a: 364. Type species: *Phorocardius solitarius* Fleutiaux, 1931: 309. Type species fixed by original monotypy.

***Displatynychus*** Ôhira, 1987: 92.

*Platynychus*: subgenus Displatynychus Ôhira, 1987: 92 (species included: monotypic). Type species: *Cardiophorus adjutor* Candèze, 1875: 17. Type species fixed by original designation.

—Ôhira 1999: 358 (at genus rank)

—Cate 2007: 206 (as subgenus of *Platynychus* without comment, catalog, Palaearctic)

*Eniconyx*Horn 1884, see: *Aptopus* Eschscholtz

***Esthesopus*** Eschscholtz, 1829: 32. Type species: *Esthesopus castaneus* Eschscholtz, 1829: 32. Type species fixed by original monotypy.

—Eschscholtz 1836: table (misspelled as *Esthosopus*, verified by possession of following key characters: 4^th^ tarsomere laminate, claws serrate)

—Germar 1839: 197 (misspelled as *Ethesopus*, verified by mention of correct type species, key to genera of Elateridae)

—Candèze 1860: 274 (species level revision of Elateridae)

—Harold 1869: 1555 (catalog)

—Horn 1884: 41 (spp. of U.S.A)

—Steinheil 1875: 131 (spp. of “Neu-Granada”)

—Henshaw 1885: 66 (catalog)

—Candèze 1891a: 137 (catalog)

—Champion 1895: 439 (spp. of Central America)

—Schwarz 1902: 269 (sp.nov)

—Schwarz 1903a: 75 (spp.n.)

—Schwarz 1906: 179 (genus level revision, catalog)

—Woodworth 1913: 198 (misspelled as *Esthesops* on P. 197 only, checklist, California USA)

—Leng 1920: 175 (catalog)

—Schenkling: 1925: 260 (catalog)

—Blackwelder 1944: 303 (checklist)

—Fleutiaux 1947b: 111 (spp. of Antilles)

—Arnett 1960: 509 (genera of USA)

—Becker 1973: 1531 (diagnostic note)

—Dolin 1975: 1632 (wing venation)

—Dolin 1978a: 8 (phylogeny of Elateridae)

—Golbach 1979a: 400 (key to Argentinean genera)

—Golbach 1994: 24 (key to genera of South America)

—Downie and Arnett 1996: 822 (spp. of northeastern North America)

—Johnson 2002: 168 (genera of USA)

—Cordoba and Aranda 2005: 102 (spp. redescriptions)

—Cordoba and Aranda 2007: 40 (sp.n., Paraguay)

***Floridelater* gen. n.** Type species *Coptostethus americanus* Horn, 1871: 303 (species included: monotypic).

—Johnson 2001: 58. Type species transferred to Negastriinae from Cardiophorinae (*Negastrius americanus* (Horn).

*Gauroderus* Thomson, 1859, see: *Dicronychus* Brullé

*Gastrimargus* Schwarz, 1902, see: *Margogastrius* Schwarz

***Globothorax*** Fleutiaux, 1891: ccxxxii. Type species: *Globothorax chevrolati* Fleutiaux, 1891: ccxxxiii. Type species fixed by original monotypy.

—Waterhouse 1902: 149

—Schwarz 1906: 181 (genus level revision, catalog)

—Schenkling: 1925: 263 (catalog, distribution)

—Blackwelder 1944: 303 (checklist)

—Golbach 1979a: 401 (sp.n., key to species)

—Golbach 1994: 24 (key to genera of South America)

—Aranda 1998: 126, 128, 130 (misspelled as *Globothorax*, verified by reference to correct author and year and appearance in context with other cardiophorine genera, key to genera)

—Rosa 2011: 1 (diagnostic characters, sp.n., key to spp. of Brazil)

*Teslasena* Fleutiaux, 1892: 410. **Syn. n.** Type species, *Anelastes femoralis* Lucas, 1857: 71, fixed by original monotypy. Described in Elaterinae: Physodactylini.

—Schwarz 1897: 9 (note)

—Fleutiaux 1899: 206 (sp.n.)

—Schwarz 1906: 313 (genus level revision, catalog)

—Schenkling: 1927: 509 (catalog, distribution)

—Chassain 2005: 66 (sp.n., Brazil)

—Rosa 2014: 227 (diagnosis, redescription, illustrations, key to species)

***Horistonotus*** Candèze, 1860: 243 (39 species included). Type species: *Horistonotus flavidus* Candèze, 1860: 250. Type species fixed by Hyslop 1921: 650. Definition expanded here to include species near *Aptopus agrestis* (Erichson).

—Harold 1869: 1554 (catalog)

—Steinheil 1875: 131 (spp. of “Neu-Granada”)

—Horn 1871: 302 (sp.n., U.S.A)

—Horn 1884: 33 (spp. of U.S.A)

—Henshaw 1885: 66 (catalog)

—Candèze 1889: 111 (sp.n.)

—Candèze 1891a: 135 (catalog)

—Fleutiaux 1895b: 172 (sp.n.)

—Schwarz 1895b: 40 (key to genera)

—Champion 1895: 428 (spp. of Central America)

—Schwarz 1897: 9 (note)

—Schwarz 1902: 261 (spp.n.)

—Schwarz 1903a: 74 (sp.n.)

—Schwarz 1906: 176 (genus level revision, catalog)

—Woodworth 1913: 198 (misspelled as *Horisonotus*, verified by two references to valid *Horistonotus* spp., checklist, California USA)

—Leng 1920: 175 (catalog)

—Schenkling: 1925: 255 (catalog, distribution)

—Van Dyke 1932: 329 (spp.n., USA)

—Carter 1939: 310 (all sp. of Australia transferred to *Paracardiophorus*)

—Blackwelder 1944: 302 (checklist)

—Fleutiaux 1947b: 111 (spp. of Antilles)

—Arnett 1960: 509 (genera of USA)

—Lanchester 1971: 48 (spp. of northwestern USA)

—Becker 1973: 1531 (diagnostic note)

—Dolin 1975: 1632 (wing venation)

—Golbach 1979b: 411 (sp.n., key to spp. of Argentina)

—Bousquet 1991: 178 (catalog)

—Carpenter 1992: 331 (misspelled as *Horizonotu*)

—Golbach 1994: 24 (key to genera of South America)

—Downie and Arnett 1996: 821 (spp. of northeastern North America)

—Wells 2000: 416 (key to the spp. of USA and Canada)

—Johnson 2002: 168 (genera of USA)

—Aranda 2009: 34 (spp.n., Argentina, redescription)

—Casari 2011: 3 (spp.n., Brazil)

—Cordoba and Aranda 2013: 4 (sp.n. Argentina)

*Lasiocerus* Buysson, 1912, see: *Cardiophorus*: subgenus Lasiocerus Buysson

*Lesnelater* Fleutiaux, 1935, see: *Pachyelater* Lesne

***Margogastrius*** Schwarz, 1903b: 80. Replacement name for *Gastrimargus* Schwarz. **Transferred here to Cardiophorinae.**

—Schwarz 1906: 312 (genus level revision, catalog)

—Schenkling: 1927: 509 (catalog, distribution)

—Rosa: 2014: 224 (phylogeny of Physodactylini, diagnosis, description of type species, figures)

*Gastrimargus* Schwarz, 1902: 309. Type species: *Gastrimargus schneideri* Schwarz, 1902: 310. Type species fixed by original monotypy. Name preoccupied by *Gastrimargus* Spix, 1823.

***Metacardiophorus*** Gurjeva, 1966: 91. **stat. n.**, raised to genus rank. *Cardiophorus*: subgenus Metacardiophorus Gurjeva, 1966: 91. Type species: *Cardiophorus sogdianus* Gurjeva, 1966: 91 (species included: *ineptus*, *sogdianus*). Type species fixed by original designation.

—Cate 2007: 202 (catalog, Palaearctic)

***Mionelater***Becker 1963: 125 (species included: monotypic). Type species: *Mionelater planatus* Becker, 1963: 126. Type species fixed by original designation. Chiapas, Mexico Miocene fossil in amber.

***Neocardiophorus*** Gurjeva, 1966: 95 (species included: *fausti*, *mamajevi*). Type species: *Neocardiophorus mamajevi* Gurjeva, 1966: 95. Type species fixed by original designation.

—Gurjeva 1974a: 112 (thoracic characters)

—Dolin 1978b: 81 (larvae, USSR)

—Iablokoff-Khnzorian and Mardjanian 1981: 246 (note)

—Cate 2007: 205 (catalog, Palaearctic)

—Platia 2008c: 207 (sp.n., Iran).

***Nyctor*** Semenov-Tian-Shanskij & Pjatakova, 1936: 101 (species included: monotypic). Type species: *Nyctor expallidus* Semenov-Tian-Shanskij & Pjatakova, 1936: 102. Type species fixed by original designation.

—Dolin 1975: 1632 (wing venation)

—Cate 2007: 194 (as unexplained synonym of *Cardiophorus*, catalog, Palaearctic)

***Odontocardus*** Fleutiaux, 1931: 332 (species included: *lateralis*, *vitalisi*, *harmandi*). Type species: *Cardiotarsus vitalisi* Fleutiaux, 1918b: 231. Type species fixed by original designation.

—Fleutiaux 1947a: 376 (spp. of French Indochina)

—Gurjeva 1974a: 112, (misspelled as *Odontocarduus*, verified by reference to cardiophorine genera)

***Pachyelater*** Lesne, 1897b: 117 Replacement name for *Parelater* Lesne.

—Lesne 1906: 172 (male association, sp.n., key to spp.) Transferred here to Cardiophorinae.

—Fleutiaux 1918a: 170 (sp.n., E. Africa)

—Fleutiaux 1921: 17 (distribution, Kenya, not placed in any subfamily)

—Schenkling 1927: 503 (catalog, distribution, in Denticollinae)

—Fleutiaux 1929b: 241 (catalog, list of types, not placed in any subfamily)

—Douglas 2011: 22 (syn. n., phylogeny)

*Lesnelater* Fleutiaux, 1935a: 116 (species included: *madagascariensis*, *dubius*, *singularis*, *unicus*). Described in Physodactylinae from males assigned to *Pachyelater* by Lesne 1906: 172. Type species *Lesnelater madagascariensis* Fleutiaux (= *Pachyelater madagascariensis* Lesne, 1897 **syn. n.**), fixed by original designation (although this combination was not used in original publication).

—Fleutiaux 1935b: 242 (sp.n., Angola)

—Arnett 1955: 612 (note on type species)

—Douglas 2011: 22 (as synonym of *Pachyelater*, phylogeny)

*Parelater*Lesne 1897a: 102. Type species *Pachyelater madagascariensis* Lesne, 1897: 102. Type species fixed by original monotypy. Not originally placed in any subfamily by Lesne, but possible proximity to Cebrionidae, Cardiophorinae and Physodactylinae mentioned. Preoccupied by *Parelater* Candèze, 1882: 70 (Coleoptera: Elateridae), not *Parelater* Candèze, 1865: 29 as according to Neave 1939: 607.

***Paracardiophorus*** Schwarz, 1895b: 40 (species included: *musculus*, *sequens*, *pullatus*, *granarius*, *erythrurus*, *subaeneus*, *fuscipennis*, *humeralis*, *australis*, *longicornis*). Type species: *Cardiophorus musculus* Erichson, 1840: 299. Type species fixed by Hyslop 1921: 660. Genus membership revised here to include North American spp. and exclude Australian and S. American spp.

—Schwarz 1902: 259 (sp.nov)

—Buysson 1902: 290 (As ‘group’ of *Cardiophorus*)

—Waterhouse 1902: 265

—Schwarz 1903d: 390 (sp.n.)

—Schwarz 1906: 172 (genus level revision, catalog)

—Heyden et al. 1908: 394 (catalog Europe, as subgenus of *Cardiophorus*)

—Jakobson 1913: 761 (key, catalog, Europe)

—Schenkling: 1925: 249 (catalog, distribution)

—Fleutiaux 1928: 254, (misspelled *Paracarpiophonus* verified by published correction, Fleutiaux 1929a: 23.

—Miwa 1927: 106 (key to genera of Japanese Empire)

—Miwa 1930: 6 (key to genera and spp. of Formosa)

—Fleutiaux 1932b: 21 (spp. of Mozambique)

—Miwa 1934: 26 (spp. of Japanese Empire)

—Carter 1939: 309 (spp. of Australia)

—Jagemann 1940: 55 (spp. Europe)

—Blackwelder 1944: 302 (checklist)

—Jagemann 1955: 152 (spp. of Czechoslovakia)

—Arnett 1955: 614 (note on types, misspelled as *Paracapiophonus*, verified by reference to Fleutiaux’s (different) misspelling of *Paracardiophorus*)

—Neboiss 1956: 40 (checklist, Australia)

—Tsherepanov 1957: 244 (spp. of W. Siberia)

—Neboiss 1961: 18 (checklist, Australia)

—Leseigneur 1972: 156 (spp. of France)

—Dajoz 1963: 172 (spp. of Europe and Mediterranean region)

—Cobos 1970a: 225 (sp.n., Congo)

—Ôhira 1973b: 38 (spp. of Ceylon)

—Gurjeva 1974a: 112 (thoracic characters)

—Dolin 1975: 1632 (wing venation)

—Dolin 1978b: 73 (larvae, USSR)

—Chassain 1979: 210 (sp.n., Saudi Arabia)

—Golbach 1979a: 400 (key to Argentinean genera)

—Lohse 1979: 186 (spp. of central Europe)

—Iablokoff-Khnzorian and Mardjanian 1981: 246 (note)

—Dolin 1988: 9 (spp. of Ukraine)

—Golbach 1994: 24 (key to genera of South America)

—Calder 1996: 360 (checklist, Australia)

—Ôhira 1997: 1 (note)

—Lucht 2005: 198-199, (misspelled *Paracardiophorus*, verified by association of type species name, author and range.)

—Platia and Gudenzi 2007: 94 (sp.n., Greece)

—Cate 2007: 205 (catalog, Palaearctic)

—Platia 2008c: 208 (sp.n., Russia)

—Pathwardhan and Athalye 2010: 510 (sp.n., India)

—Platia 2010a: 128 (sp. transfer from *Cardiophorus*)

*Paradicronychus* Dolin & Gurjeva, 1975, see: *Cardiophorus* Eschscholtz

*Paramecus* Dillwyn, 1829, see: *Dicronychus* Brullé

***Paraplatynychus*** Fleutiaux, 1931: 315.

*Platynychus*: subgenus Paraplatynychus Fleutiaux, 1931: 315 (species included: *mixtus*, *costatus*, *fouqueti*, *incostatus*). Type species: *Platynychus mixtus* Fleutiaux, 1931: 326. Type species fixed by original designation.

—Ôhira 1970: 230 (at genus rank without comment, types from Southeast Asia, with new combination *Paraplatynychus costatus* (Fleutiaux, 1931) from Platynychus (Paraplatynychus) costatus.

—Fleutiaux 1947a: 348 (as subgenus of *Dicronychus*, spp. of French Indochina)

*Parelater* Lesne, see: *Pachyelater* Lesne

*Patriciella* Van Zwaluwenburg, see: *Aphricus* LeConte

*Patricia* Van Zwaluwenburg, see: *Aphricus* LeConte

*Perrinellus* Buysson, see: *Cardiophorus*: subgenus Perrinellus Buysson

***Phorocardius*** Fleutiaux, 1931: 308 (species included: *astutus*, *bifidus*, *florentini*, *magnus*, *melanopterus*, *solitarius*, *unguicularis*,). Type species: *Cardiophorus florentini* Fleutiaux, 1895a: 687. Type species fixed by original designation.

—Miwa 1934: 26 (spp. of Japanese Empire)

—Fleutiaux 1947a: 363 (spp. of French Indochina)

—Ôhira 1971: 207 (types from South Asia)

—Gurjeva 1974a: 112 (thoracic characters)

—Cate 2007: 206 (catalog, Palaearctic)

—Platia 2015: 184 (sp.n. Maldives)

***Platynychus*** Motschulsky, 1858: 58 (species included: *pictus*, *curiatus*, *mauritanicus*, *indicus*, *nebulosus*, *crucifer*, *axillaris*). Type species: *Platynychus indicus* Motschulsky, 1858: 59. Type species fixed by Hyslop 1921: 665. This fixation is valid and non-reversible, although disputed by Méquignon 1930: 96.

—Motschulsky 1858: 59 (misspelled as *Platynchus*, described under correct spelling in earlier paragraph)

—Harold 1869: 1546 (partially as synonym of *Cardiophorus*, catalog)

—Candèze 1891a: 121 (partially as synonym of *Cardiophorus* , catalog)

—Buysson 1902: 329 (as subgenus of *Cardiophorus*, including *Elater cinereus*, senior synonym of type species of *Dicronychus*)

—Heyden et al. 1908: 394 (as subgenus of *Cardiophorus*, catalog Europe)

—Schenkling: 1925: 219 (as subgenus of *Cardiophorus*, catalog, distribution)

—Schenkling 1925: 238 (as synonym of *Dicronychus*, catalog, distribution)

—Fleutiaux 1931: 315 (catalog French Indochina)

—Méquignon 1931: 207 (as synonym of *Dicronychus*, and *Dicronychus* there considered a subgenus of *Cardiophorus*, also misspelled as *Platynychus*, on p 208. verified by use of correct spelling elsewhere in note and reference to correct publication date, note)

—Fleutiaux 1932b: 25 (spp. of Mozambique)

—Miwa 1934: 26 (spp. of Japanese Empire)

—Jagemann 1940: 55 (as subgenus of *Cardiophorus*, spp. Europe)

—Fleutiaux 1947a: 348 (spp. of French Indochina, with diagnostic characters for this and *Dicronychus*)

—Fleutiaux 1947a: 348 (as subgenus of *Dicronychus*, spp. of French Indochina)

—Jagemann 1955: 147 (spp. of Czechoslovakia)

—Van Zwaluwenburg 1957: 62 (spp. of Micronesia)

—Van Zwaluwenburg 1963: 339 (spp. of Papua New Guinea)

—Dajoz 1963: 171 (but with *Platynychnus cinereus* (valid name of *Dicronychus* type species) incorrectly named as type species, spp. of Europe and Mediterranean region)

—Chûjô and Ôhira 1965: 28 (as subgenus of *Dicronychus*,)

—Gurjeva 1966: 92 (as subgenus of *Cardiophorus*, spp. of central Asia)

—Cobos 1970a: 223 (as subgenus of *Cardiophorus*, key to genera, Congo)

—Ôhira 1970: 230 (as synonym of *Dicronychus*, moved one *Platynychus* sp. to *Dicronychus* and elevated *Paraplatynychus* to genus rank without comment. These transfers are considered possible evidence of acceptance of synonymisation of *Platynychus* under *Dicronychus*)

—Leseigneur 1972: 151 (as synonym of *Dicronychus*, spp. of France)

—Ôhira 1973a: 32 (as subgenus of *Dicronychus*, spp. of Ryukyu Arch.)

—Ôhira 1973b: 38C (as synonym of *Dicronychus*, made *Dicronychus comptus* (Candèze) new combination from Cardiophorus (Platynychus) comptus)

—Dolin and Gurjeva 1975: 118, (misspelled as *Platynychus*, verified by use as subgenus of *Cardiophorus*, which contains sp. “Cardiophorus (Platynichus) cinereus (Herbst)”)

—Chassain 1979: 195 (spp. of Saudi Arabia)

—Lohse 1979: 184 (as synonym of *Dicronychus*, spp. of central Europe)

—Iablokoff-Khnzorian and Mardjanian 1981: 246 (as synonym of *Dicronychus*, note)

—Ôhira 1987: 92 (description of new subgenus of *Platynychus*)

—Dolin 1988: 10 (as synonym of *Cardiophorus*, spp. of Ukraine)

—Cate 2007: 206 (catalog, Palaearctic)

***Ryukyucardiophorus*** Ôhira, 1973a: 32 (species included: monotypic). Type species: *Paracardiophorus loochooensis* Miwa, 1934: 255. Type species fixed by original designation.

—Platia and Gudenzi 1999: 23 (new species, Turkey)

—Cate 2007: 207 (catalog, Palaearctic)

—Platia 2015: 182 (sp.n. Maldives)

*Teslasena* Fleutiaux, see: *Globothorax* Fleutiaux

***Triplonychoidus*** Schwarz, 1906: 181 (species included: *trivittatus*, *parvulus*). Type species: *Triplonychus trivittatus* Champion, 1895: 427. Type species fixed by Hyslop 1921: 672.

—Hyslop 1921: 672 (misspelled as *Triplonychoides*, verified by reference to correct author year, and page number)

—Schenkling: 1925: 263 (misspelled as *Triplonychoides*, catalog, distribution)

—Blackwelder 1944: 303 (misspelled as *Triplonychoides*, checklist)

—Golbach 1994: 24 (misspelled as *Triplonychoides*, key to genera of South America)

***Triplonychus*** Candèze, 1860: 236 (species included: *ephippiger*, *acuminatus*, *longicollis*, *cayennensis*, *ventralis*, *plagiatus*, *lebasii*, *costatus*, *rufus*, *debilis*). Type species: *Triplonychus acuminatus* Candèze, 1860: 238. Type species fixed by Hyslop 1921: 672.

—Harold 1869: 1553 (catalog)

—Candèze 1891a: 135 (catalog)

—Champion 1895: 426 (spp. of Central America)

—Schwarz 1906: 180 (genus level revision, catalog)

—Schenkling: 1925: 262 (catalog, distribution)

—Blackwelder 1944: 303 (checklist)

—Golbach 1979a: 400 (key to genera of South America)

—Golbach 1994: 24 (key to genera of South America)

—Rosa 2011: 3 (diagnostic characters, spp.n., Brazil, key to spp. of Brazil)

***Tropidiplus*** Fleutiaux, 1903: 251. Type species: *Tropidiplus tellinii* Fleutiaux, 1903: 251. Type species fixed by original monotypy.

—Schwarz 1906: 174 (as synonym of *Craspedostethus*, genus level revision, catalog)

—Waterhouse 1912: 310

—Fleutiaux 1932b: 22 (spp. of Mozambique, apparently ignoring synonymy by Schwarz)

—Cobos 1970a (as synonym of *Paracardiophorus*, subgenus Craspedostethus)

—Chassain 1979: 206 (rejecting placement in *Paracardiophorus* based on sclerites of bursa copulatrix, spp. of Saudi Arabia)

—Cate 2007: 203 (as synonym of *Craspedostethus*, catalog, Palaearctic)

***Zygocardiophorus*** Iablokoff-Khnzorian & Mardjanian, 1981: 247 (species included: *nigratissimus*, *alienus*). **Stat. n**. raised to genus rank. Type species, *Cardiophorus nigratissimus* Buysson, 1891: 134, type species fixed by original designation. Described as subgenus of *Cardiophorus*.

—Platia 1994: 354 (misspelled as *Zigocardiophorus*, verified by correct page and date of original publication, as subgenus of *Cardiophorus*)

## Supplementary Material

XML Treatment for Austrocardiophorus

XML Treatment for Chileaphricus

XML Treatment for Paracardiophorus

XML Treatment for Floridelater

XML Treatment for Allocardiophorus

XML Treatment for Aphricus

XML Treatment for Aptopus

XML Treatment for Blaiseus

XML Treatment for Buckelater

XML Treatment for Cardiodontulus

XML Treatment for Cardiophorellus: subgenusCardiophorellus

XML Treatment for Cardiophorellus: subgenusParapleonomus

XML Treatment for Cardiophorus: subgenusCardiophorus

XML Treatment for Cardiophorus: subgenusCoptostethus

XML Treatment for Cardiophorus: subgenusPerrinellus

XML Treatment for Cardiotarsus

XML Treatment for Craspedostethus

XML Treatment for Dicronychus

XML Treatment for Diocarphus

XML Treatment for Displatynychus

XML Treatment for Esthesopus

XML Treatment for Globothorax

XML Treatment for Horistonotus

XML Treatment for Margogastrius

XML Treatment for Metacardiophorus

XML Treatment for Mionelater

XML Treatment for Neocardiophorus

XML Treatment for Nyctor

XML Treatment for Odontocardus

XML Treatment for Pachyelater

XML Treatment for Paraplatynychus

XML Treatment for Phorocardius

XML Treatment for Platynychus

XML Treatment for Ryukyucardiophorus

XML Treatment for Triplonychoidus

XML Treatment for Triplonychus

XML Treatment for Tropidiplus

XML Treatment for Zygocardiophorus

## APPENDIX I

### Specimens examined

Specimens coded and identification information sources used for species used in phylogenetic analysis. Entries include information in the following sequence: species name, photo codes of specimens coded (Appendix II, M/F indicate sex), type # for type specimens examined (Appendix III), references to publications other than the original description used to identify specimens coded (format = lit.: name), names appearing on determination labels of non-type specimens (format = det.: name), an indication of whether the species is the type of its genus, and the subfamily to which the species belongs.

**Outgroups**

*Adrastus pallens* (Fabricius, 1792), M: P134, F: P135, lit.: Leseigneur 1972, det: Wellschmeid 1962, non-type species (Elaterinae: Adrastini)

*Agriotes sputator* (Linnaeus, 1758), T069 (T070 to T072 not conspecific) M: P2C2; F: P2B6, P2C1, type species (Elaterinae: Agriotini)

*Agrypnus murinus* (Linnaeus, 1758), M: P072; F: P073, lit.: Leseigneur 1972, det.: none, type species (Agrypninae)

*Athous vittatus* (Fabricius, 1792), M: P104, P105; F: P106, P107, lit.: Leseigneur 1972, det.: none, type species, (Dendrometrinae)

*Berninelsonius hyperboreus* (Gyllenhal, 1827), M: P203; F: P204, lit.: Stibick 1979b, det.: none, type species, (Dendrometrinae: Hypnoidini)

*Elater ferrugineus* Linnaeus, 1758, M: P046; F: P047, T066, T067, lit.: Leseigneur 1972, det.: Anonymous, type species, (Elaterinae)

*Hypnoidus riparius* (Fabricius, 1792), M: P133; F: P131, P132, lit.: Stibick 1979b, det.: Olexa ‘73, type species, (Dendrometrinae: Hypnoidini)

*Tropihypnus bimargo* Reitter, 1896, M: P2D2; F: P2D1, lit.: Stibick 1968, det.: Gurjeva, type species, (Dendrometrinae)

**Physodactylinae**

*Margogastrius schneideri* Schwarz, 1902, F: T058, T059, det.: none, type species

*Pachyelater madagascariensis* (Lesne, 1897), M: T024; F: T026, T027, lit.: Douglas 2011, det.: types, type species

*Teslasena femoralis* Lucas, 1857, M: P061, T039, lit.: Fleutiaux 1892, det.: Fleutiaux, type species

**Negastriinae**

*Agrypnella eburnea* Champion, 1895, M: T008, P025, det.: E.C. Becker 1985, type species

*Agrypnella squamifer* Candèze, 1895, F: P029, det.: none

*Arhaphes diptychus* Candèze, 1860, M: P015; F: P016, det.: E. Candèze, type species,

*Cardiohypnus mirabilis* (Candèze, 1860), M: P028a, c; F: P028b, T009, lit: Fleutiaux 1947a, det.: none, type species

*Fleutiauxellus maritimus* (Curtis, 1840), M: P185; F: P186, lit.: Leseigneur 1972, det.: Dolin 1969, type species

*Migiwa curatus* Candèze, 1875, M: P179; F: P180, lit.: Kishii 1956, det.: unknown, non-type species

*Monadicus* sp., M: P181; F: P182, lit.: Candèze 1860, det.: none, non-type species

*Negastrius americanus* (Horn, 1871), M: P157, F: P156, T095, det.: none, non-type species

*Negastrius pulchellus* Fabricius, 1801, M: P045; F: P013, P014, lit.: Leseigneur 1972, det. Olexa 73, type species

*Neoarhaphes americanus* Champion, 1895, M: P187; F: P188, lit.: Costa 1966, det.: none, non-type species

*Neohypdonus gentilis* (LeConte, 1866), M: P177; F: P178, lit.: Stibick 1990, det.: none, type species

*Oedostethus femoralis* LeConte, 1853, M: T016, P012; F: P010, lit.: Stibick 1990, det.: P. Bélanger, type species

*Paradonus pectoralis* (Say, 1836), M: P175; F: P176, lit.: Stibick 1990, det.: none, type species

*Pronegastrius humeralis* (Candèze, 1863), F: P183, P184, lit.: Ôhira 1963, det.: [From Ôhira Coll.], type species

*Quasimus minutissimus* Germar, 1817, M: P017; F: P018, T081, lit.: Leseigneur 1972, det.: Olexa ‘73, type species

*Rivulicola variegatus* (Macleay, 1872), M: P021a, b; F: P022, det.: Anonymous, type species

*Yukoana carinicollis* Lewis, 1894, M: P191; F: P192, lit.: Kishii 1970, det.: Ôhira 1967, non-type species

*Zorochros demustoides* Herbst, 1806, M: P2C4; F: P2C3, T082, lit.: Leseigneur 1972, det.: none, type species

**Cardiophorinae**

*Aphricus californicus* LeConte, 1853, M: P034, T020, lit.: Knull 1957, det.: Douglas 2004, type species

*Aphricus chilensis* Fleutiaux, 1940, M: T036; F: none, det.: none, non-type species

*Aptopus agrestis* Erichson, 1840, M: P142, P143; F: none, det.: none, non-type species

*Aptopus pullatus* (Horn, 1884), M: P7A1–3; F: P036, T017, T018, lit.: Schaeffer 1916, det.: none, type species

*Blaiseus bedeli* Fleutiaux, 1931, M: T034; F: none, det.: none, type species

*Blaiseus nothoafricanus* Douglas, 2009, M: P170, P171, lit.: Fleutiaux 1931, det.: none, non-type species

*Buckelater argutus* Costa, 1973, M: P098–102, T045– T049; F: none, det.: none, type species

*Cardiodontulus brandti* Van Zwaluwenburg, 1963, M: T001, T002; F: none, det.: none, type species

*Cardiophorellus gracilicornis* Cobos, 1970, M: T028, T029, det.: none, type species

Cardiophorus (Coptostethus) brunneipennis Wollaston, 1864, M: P155; F: P154, lit.: Cobos 1970b, det.: Platia 2002, non-type species

Cardiophorus (Metacardiophorus) sogdianus Gurjeva, 1966, M: T056; F: none, det.: none, type species

Cardiophorus (Perrinellus) angustatus Fleutiaux, 1933, M: P059, P060; F: none, T038; F: none, det.: none, non-type species

Cardiophorus (Perrinellus) argentatus Abeille de Perrin, 1894, M: P069, T043, lit.: Buysson 1899, det.: none, type species

Cardiophorus (Zygocardiophorus) nigratissimus Buysson, 1891, M: P149, P151; F: P150, P152, lit.: Platia and Gudenzi 2002, det.: Platia 2002, type species

*Cardiophorus brevis* Candèze, 1887, M: P168; F: P169, lit.: Champion 1895, det.: none, non-type species

*Cardiophorus cardisce* (Say, 1834), M: P6A5, P2A12, P2C6, P2B1, P158, P159; F: P5A2, P5A3, FAB8, T074, lit.: Douglas 2003, det.: Douglas 2003, non-type species

*Cardiophorus convexulus* LeConte, 1853, M: P167, P1H3; F: P1H1, P3H3, T062, lit.: Douglas 2003, det.: Douglas 2003, non-type species

*Cardiophorus convexus* (Say, 1823), M: P1A5, P1C10, P3B6, PBA1, PBA2, F: P1F4, P1F6, P4A3, P5D1, PBA3, PBA4, T063, lit.: Douglas 2003, det.: Douglas 2003, non-type species

*Cardiophorus gramineus* (Scopoli, 1763), M: PBB1; F: P020, P023, P141, T012, T013, lit.: Leseigneur 1972, det.: Anonymous, type species

*Cardiophorus inflatus* Candèze, 1882, F: T005; M: none, det.: none, non-type species

*Cardiophorus luridipes* Candèze, 1860, M: P165; F: P164, T065, lit: Blanchard 1889, det.: Blanchard 1889, non-type species

*Cardiophorus* sp. undescribed, Congo, M: PBC4; F: C5, lit.: Candèze 1860, det.: none, non-type species

*Cardiophorus togatus* Horn, 1871, M: P163, P1G9, P1G10; F: P5B2, T064, lit.: Douglas 2003, det.: Douglas 2003, non-type species

*Cardiotarsus capensis* Candèze, 1860, P144–146 & 2M &3F, T075, det.: none, type species

*Cardiotarsus mjobergi* Carter, 1939, M: P172; F: P173, det.: none, non-type species

*Cardiotarsus* sp., M: HDCU222, HDCU223

*Craspedostethus rufiventris* Schwarz, 1898, M: P053; F: T060, T061, det.: none, type species

*Dicronychus cinereus* (Herbst, 1784), M: PBB2; F: P153, T080, lit.: Leseigneur 1972, det.: Platia 2002, type species

*Esthesopus castaneus* Eschscholtz, 1829, M: P049; F: P050, P051, T076, lit.: Candèze 1860, det.: Becker ‘59, Hayek ‘85, type species

*Esthesopus parcus* Horn, 1884, M: P7B2, P7B4; F: P7B3, P116, P117, T090, det.: none, non-type species

*Globothorax chevrolati* Fleutiaux, 1891, F: T037; M: none, det.: none, type species

*Horistonotus flavidus* Candèze, 1860, M: P097; F: T050 (=P095), T051 (=P096), det.: none, type species

*Horistonotus simplex* LeConte, 1863, M: P7A5, P7A6; F: P7A4, P7B1, P160, T088, lit.: Wells 2000, det.: none, non-type species

*Neocardiophorus mamajevi* Gurjeva 1966, M: T055; F: none, det.: none, type species

*Nyctor expallidus* Semenov-Tian-Shanskij & Pjatakova, 1936, M: T052, T053; F: T054, det.: none, type species

*Odontocardus vitalisi* Fleutiaux, 1918, M: P067; F: P068, T042, lit.: Fleutiaux 1947a, det.: Fleutiaux, type species

*Paracardiophorus humeralis* (Fairmaire & Germain, 1860), M: PBD2; F: PBD3, P190, HDCU215, det.: Candèze, von Hayek, non-type species

*Paracardiophorus musculus* (Erichson, 1840), F: PBB3; M: PBB4, T077–T079, lit.: Leseigneur 1972, det.: J. Mertlik, type species

*Paracardiophorus subcruciatus* Carter, 1939, M: PBA5; F: PBA6, P189, type not found, det.: none, non-type species

*Patriciella australica* Van Zwaluwenburg, 1947, M: T044, det.: none, type species

*Phorocardius florentini* Fleutiaux, 1895a, M: P056; F: P057; sex?: T035, lit.: Fleutiaux 1947a, det.: none, type species

*Phorocardius solitarius* Fleutiaux, 1931a, F: T033, lit.: Fleutiaux 1947a, det.: none, type species

*Platynychus adjutor* Candèze, 1875, M: P003, F: P002, T093, lit.: Ôhira 1987, det.: none, type species

*Platynychus indicus* Motschulsky, 1858, F: T041; M: none, det.: none, type species

*Paraplatynychus mixtus* Fleutiaux, 1931a, M: P065, P066; F: P064, T094, lit.: Fleutiaux 1947a, det.: Fleutiaux, type species

*Ryukyucardiophorus loochooensis* (Miwa, 1934), M: P272, P273; F: P271, det.: Ôhira 1971, type species, (not in phylogeny)

sp. undescribed, New Zealand, M: P005, lit.: Broun1893, Douglas 2003, det.: none, non-type species

*Triplonychoidus trivittatus* (Champion, 1895), M: T010, T011, det.: none, type species

*Triplonychus plagiatus* (Erichson, 1840), M: P148 & 6 others; F: P147, T083, Candèze 1860, det.: Douglas 2005, non-type species

*Tropidiplus tellinii* Fleutiaux, 1903, M: T040; F: P063, det.: von Hayek 1965, type species

## APPENDIX II

### Non-type specimens

Label data from non-type specimens of species coded for phylogenetic analyses, including information in the following order: scientific name and author, photo code number, identification code number from voucher label (begins with HDCU), label data, the sex of the specimen (M/F), and the coden representing the specimen depository. Text from multiple labels, beginning with uppermost label, are listed in a separate set of quotation marks, separated by semicolons. Text from each line of a label with multiple lines of text is separated by a “/”. Notes about the appearance of the label appear in brackets before quotation.

*Adrastus pallens* (Fabricius), P134, HDCU138, “Germany/ Holstein, Plön/ 26. Vii. 1964/ G. G. E. Scudder”, [partly handwritten] “Adrastus/ pallens F. 1967/ Wellschmeid det.”, M, CNCI; P135, HDCU139, [partly handwritten] “22.VII.1924 Finl./ Kuolemajärvi/ M.Ivaschinzeff”, [partly handwritten] “Adrastus F#/ pallens Fabr./ Dr. Wellschmeid det. 1962”, F, CNCI

*Agriotes sputator* (Linnaeus), P2C2 “Sydney, N.S./ VI–7–1965/ W.J.Brown”, M, CNCI; P2B6 “Sydney, N.S./ VI–7–1965/ W.J.Brown”, F, CNCI; P2C1 “North Sydney/ N.S. VI–19–1965/ W.J. Brown”, F, CNCI

*Agrypnella eburnea* Champion, P025, HDCU026, “Santarem, Para/Brazil IV–27–/1963 F.Werner”; [partly handwritten] “Agrypnella/ nr. eburnea/ MCZ has 3/ Det. E.C. Becker 1985” [partly handwritten] “Agrypnella/ eburnea/ BMNH –Homotype/ Det.HDouglas 2004” M, CNCI

*Agrypnella squamifer* Candèze, P029, HDCU033, [blue disc, handwritten] “Santar/ em”; [handwritten] “squamifer/ Cdz./ (named from type).”; [partly handwritten] “mentioned/ B.C.A.III.(1)415”, F, NHM

*Agrypnus murinus* Linnaeus, P072, HDCU076, [Partly handwritten] “Suisse-Lausanne/ Vidy/ 6 VI 42/ J. Aubert”, M, CNCI; P073, HDCU077, “France, Landes/ Parentis/ VI. 1977/ Vuillaume”, F, CNCI

*Aphricus californicus* LeConte, P034, HDCU038, “Congress Jct./ Ar. 7–28–33/ R.H.Beamer”; [partly handwritten] “Aphricus/ californicus/ LeC./ Det.HDouglas 2004”, M, CNCI

*Aptopus agrestis* Erichson, P142, HDCU146, “Faz.Aceiro/ Jatai, Goiás-Brasil/ X. 1962/ Exp.Dep.Zool.”, M, MZSP; P143, HDCU147, “Faz.Aceiro/ Jatai, Goiás-Brasil/ X. 1962/ Exp.Dep.Zool.”, M, MZSP

*Aptopus pullatus* (Horn), P036, HDCU040, “Baboquivari, Mts./ Ariz. VII–24–41/ E.L. Todd”; [partly handwritten] “Aptopus ♀?/ pullatus (Horn)/ Det.Hdouglas 2003” F, SEMC

*Arhaphes diptychus* Candèze, P015, HDCU015, “Coll. R.I.Sc.N.B/ Inde/ Tenasserim/ Thagata/ Fea. Apr. 1887”; “Collection/ E. Candèze”; [handwritten] “Arrhaphes/ diptychus Cd. Dét. E. Candèze”, F, ISNB; P016, HDCU016, same data, M, ISNB

*Athous argentatus* Perrin, P069, HDCU073, [male symbol]; [handwritten] “Jerusalem/ D David”; MUSEUM PARIS/ Collection Léon Fairmaire/ 1906”, M, MNHN

*Athous vittatus* (Fabricius), P104, HDCU108, [partly handwritten] “CZECHOSLOVAKIA/ Mor. Bor. 24.v.1963/ Moravicany/ leg.V.Korbel”, M, CNCI; P105, HDCU109, same data, M, CNCI; P106, HDCU110, same data, F, CNCI; P107, HDCU111, same data, F, CNCI

Cardiophorinae sp., P005, HDCU005, “Rika Valley/ Clarence River/KA 12 Mar 69/ R.J.B. Power” “Swept grass Coprosoma” “Brounaeolus Hyslop” “N Z Arthropod/ Collection, NZAC/ Private Bag 92170/ AUCKLAND/ New Zealand” “nr Brounaeolus/ det. R.A. Leschen”, M, NZAC

*Berninelsonius hyperboreus* Gyllenhal, P203, HDCU207, “Y.T., British Mts./ “June Cr.”, 320m/ 3km NW Firth R./ 69°13’ , 140°05’/ J.M.Campbell”; “25.VI.1984/ 84–32 , misc./ beetles ex/ around camp”, M, CNCI; P204, HDCU208, same data, “29.VI.1984/ 84–37 , sifting/ Salix & Alnus/ litter”, F, CNCI

*Cardiohypnus mirabilis* Candèze, P028, HDCU029, 6 specimens on a pin labelled 6X “Haldwani Div./ Kumaon,/ India. H.G.C.”; 1X “H.G.Champion Coll./ B.M. 1953–156.”, now 4 on pin and 4 labels, NHM; P028a, HDCU030, same data, M, NHM; P028b, HDCU031, same data, F, NHM; P028c, HDCU032, top specimen of remaining 4 on pin, M, NHM

*Cardiophorus brevis* Champion, P168, HDCU172, “Mex: Oaxaca/ 20 mi. W. Pinotepa/ Nacional/VI.23.1983, M, CNCI; P169, HDCU173, “Mex: Oaxaca/ 20 mi. W. Pinotepa/ Nacional/VI.23.1983, F, CNCI

*Cardiophorus cardisce* (Say), P158, HDCU162, “Canada, ON, Lanark Co./ Almonte, on sand 16.V.04/ Hdouglas, Mlarivee, M?, CNCI; P159, HDCU163, “Canada, QC, Co. Pontiac, Bristol Mines, 45°30’ 00” N 76°21’00”W, 20.IV.03 SandDune/Pit, on sand , 11 AM HDouglas, Mlarivee”, M, CNCI

*Cardiophorus convexulus* LeConte, P167, HDCU171, [handwritten] “ON, Sandbanks/ PP. VI.16.2000/ Beating, H.Douglas”; Cardiophorus/ convexulus LeC./ Det.Hdouglas 2002”, M, CNCI

Cardiophorus (Coptostethus) brunneipennis Wollaston, P154, HDCU158, [Spain, handwritten] “IS. TENERIFE/ MASS. TENO/ Mt. DE L’AGUA/ 5.2.1991 m.800/ I. Gudenzi”; “COPTOSTETHUS/ brunneipennis Wol/ det. Platia 2002”, F, CNCI; P155, HDCU159, same data, M, CNCI

*Cardiophorus gramineus* Scopoli, P020, HDCU020, “Schleissheim/ 28.5.04”; München/ k.Ku zer”; [handwritten] “Cardiophorus/ gramineus Scop.”, F, CNCI; P023, HDCU024, [handwritten] “ROMA: E.U.R./ 16.V.’58.PARENTI”, F, CNCI; P141, HDCU145, [handwritten partly illegible] “Cerva U________”; [green square]; “BROOKLYN/ MUSEUM/ COLL. 1929”; USNM 2036286”, F, USNM

*Cardiophorus luridipes* Candèze, P164, HDCU168, “CALIFORNIA:/ Riverside Co.,/ 16 May 1924/ W. Benedict”, F, CNCI; P165, HDCU169, “CALIFORNIA:/ Riverside Co.,/ 16 May 1924/ W. Benedict”; [partly handwritten] “Cardiophorus/ luridipes/ Cand./ Det.Hdouglas 2003”, M, CNCI

*Cardiophorus nigratissimus* Buysson, P149, HDCU153, “Turkey-Gaziantep/ 18 km NW Kilis/ Kara Deresi river”; “m 420, 30.V.2002/ C.Giusto-S.Zoia”; “CARDIOPHORUS/ nigratissimus Buys./ det Platia 002”, M, CNCI; P150, HDCU154, same data, F, CNCI; P151, HDCU155, same data, M, CNCI; P152, HDCU156, same data, F, CNCI

*Cardiophorus* sp. P166, HDCU170, “Rep. Of Congo/Voka/V.1978 G. Onore”, F, CNCI

*Cardiophorus togatus* Horn, P163, HDCU167, “MEXICO: Nuevo Leon/ 37 km Linares, 1545 m/ 20 March 1991, Brooks/ Leschen. #29. Ex: beating”; [Partly Handwritten] “Cardiophorus/ togatus/ Horn/ Det.Hdouglas 2005”, M, SEMC

Cardiophorus (Perinellus) angustatus Fleutiaux, P059, HDCU063, “MAHATSINJO/ près Tananarive”, M, MNHN; P060, HDCU064, “MAHATSINJO/ près Tananarive”, M, MNHN

*Cardiotarsus capensis* Candèze, P144, HDCU148, “Winburg, OFS./ Aug., 1918./C.W. Mally./SN. 2543.”; [partly hadwritten] “ac.c.2450/ Ag Dp S Afr”; “NATIONAL COLL./ OF INSECTS/ Pretoria, S.Afr” M, SANC; P145, HDCU149, same data, M, SANC; P146, HDCU150, same data, F, SANC

*Cardiotarsus mjobergi* Carter, P172, HDCU176, “Australia, Qld. Mt./ Glorious 27°19'S,/ 152° 45'E, 1–5.XII.1997/ T. Hiller Malaise”, M, CNCI; HDCU177, same data, F, CNCI

*Cardiotarsus* sp. (nr. philautus), HDCU222 “Pietermaritzburg,/ Natal, S. Afr./ 23.XI.–5.XII.70/ H. & M. Townes”, F, CNCI; HDCU223, same data, M, CNCI

*Craspedostethus rufiventris* Schwarz, P053, HDCU057, “Kamerun/ Conradt”; “Coll. Kraatz”, M, Eberswalde; P054, HDCU058, “Kamerun/ Conradt”; “Coll. Kraatz”, M, Eberswalde

*Dicronychus cinereus* (Herbst), P153, HDCU157, [Italy, partly handwritten] “RAVENNA/ P. S. VITALE/ 16vi84/ LEG. CALLEGARI”; “DICRONYCHUS/ cinereus (Hbst.)/ det. Platia 002”, f, CNCI

*Elater ferrugineus* (Linnaeus), P046, HDCU050, unlabelled, M, CNCI; P047, HDCU051, [handwritten] “L. ferugi/ neus. 20.6/ Tero. Ital”; “343”, F, CNCI

*Esthesopus castaneus* Eschscholtz, P049, HDCU053, [handwritten] “Brazil”; “Sammlung/ CI Müller”; [partly handwritten] “Esthesopus/ castaneus Esch./ E.C. Becker 1959”, M, CNCI; P050, HDCU054, [handwritten] “Fry/ Rio. Jan/”; [partly handwritten]; “Fry Coll./ 1905. 100.”; “Esthesopus/ castaneus Er/ C.M.F. von Hayek det. 1985”; [handwritten] “Mandibles open”, F, NHM; P051, HDCU055, [handwritten] “Bahia”; “Coll. Janson./ Ex Mniszech.”; [handwritten] “Esthesopus/ castanueus Esch./ Cand. Cdz./ ex. Coll. De Mniszech.””, F, NHM

*Esthesopus parcus* Horn, P116, HDCU120, “AZ, Santa Cruz Co./ Peña Blanca Campgr./ 6 Aug 2002/ H. Douglas BL”, F, CNCI; P117, P161, HDCU165, same data, F, CNCI; P162, HDCU166, same data, M, CNCI; HDCU121, “AZ, Pima Co., Green Valley, on tree bark, 860m, 5 AUG/ 2002 H.Douglas”, ?, CNCI;

*Fleutiauxellus maritimus* (Candèze), P185, HDCU189, [partly handwritten] “Grünwald/ 23.5.49.”; “München/ Bühlmann”; [handwritten] “Hypnoidus/ maritimus Cand.”, M, CNCI; P186, HDCU190, Zakarpatny,/ Skope/ Pes Na Gorah / vii. 1966”; [partly handwritten] “Negastrius/ maritimus (Cand.)/ W. Dolin det. 1969”, F, CNCI

*Globothorax chevrolati* Fleutiaux, P058, HDCU062, “261”; [handwritten] “Brésil”; “Collection Chevrolat”; F, MNHN

*Horistonotus flavidus* Candèze P097, HDCU101, “Poá/ SP Brasil/ 10.XII.1966/ E.X.Rabello Col.”, M, MZSP;

*Horistonotus simplex* LeConte P160, HDCU164, “AZ Santa Cruz Co./ Pena Blanca Campgr./ 6 Aug 2002/ H.Douglas BL”, F, CNCI;

*Hypnoidus riparius* Fabricius P131, HDCU135, [partly handwritten]”Jilmeica/Boh. 16.V.48/A. Olexa”; [handwritten] “Hypnoidus/ riparius/ F./ Det. Olexa ‘73”, F, CNCI; P132, HDCU136, [partly handwritten] “Le Sancy/ P. Ole D./ 1.VII.72/ G. Minet”, F, CNCI; P133, HDCU137, [partly handwritten] “Le Sancy/ P. Ole D./ 1.VII.72/ G. Minet”, ?, CNCI

*Migiwa curatus* (Candèze), P179, HDCU183, [partly handwritten] “Mt. Fuji. JAPAN/ 3.VIII.1964/ M. Suda leg.”; [handwritten] “Negastrius/ curatus/ [name also written in Japanese characters], M, CNCI; P180, HDCU184, “Mt.Koya, Nara-ken/ Japan 17–VII–55/ S. Mizobe”, F, CNCI

*Monadicus* sp., P181, HDCU185, “Argentina, Prov.Bs.As/ Bs.As., San Isidro/ 10–15.I.1982/ H & A Howden”, M, CNCI; P182, HDCU186, F, same data

*Negastrius americanus* (Horn), P156, HDCU160, “USA, FL, St. Theresa,/ 7.V.2000, sifting sand &/ grass, sheltered beach,/ H.Douglas”, F, CNCI; P157, HDCU161, same data, M, CNCI

*Negastrius pulchellus* (Linnaeus), P013, HDCU013, “Jarov/ Boh.v.54./A.Olexa” [handwritten] “Hypnoidus/ pulchellus/ L./ Det.Olexa 73”, F, CNCI; P014, HDCU014, “589”; “Fennia/ Ik Ollila/ 12/6 1932/ K Lahtivirta” [handwritten] “Hypnoidus/ pulchellus L.” F, CNCI; P045, HDCU049, [handwritten] “Europe”, M, CNCI

*Neoarhaphes americanus* (Champion), P187, HDCU191, “Paraiso CZ/ PanApr 22 11/ EASchwarz”, M, CNCI; P188, HDCU192, “COSTA RICA. Punt./ 3 km N. Guacimal/ 800m 26.V.1979/ H & A Howden”, F, CNCI

*Neohypdonus gentilis* (LeConte), P177, HDCU181, “Saskatoon/ Saskatchewan/ VI–22–1974/ E.J.Kitely”, M, CNCI; P178, HDCU182, same data, F, CNCI

*Odontocardus vitalisi* Fleutiaux, P067, HDCU071, [partly handwritten] “MUSEUM PARIS/ Laos/ ou Cambodge/ Bonette 1911”, M, MNHN; P068, HDCU072, same data, “Cardiotarsus/ Vitalisi/ Fleut./ FLEUTIAUX det.”; F, MNHN

*Oedostethus femoralis* LeConte, P010, HDCU010, “CANADA Québec/ Brome Co.; Glen-/ Sutton 24.VII.1985/ Larochelle, Larivière”; [handwritten] “Oedostethus/ femoralis LeC./ Dét.: P. Bélanger, 1987”, F, CNCI; P011, HDCU011, same data, F, CNCI; P012, HDCU012, “CANADA Québec/ Brome Co.; Glen-/ Sutton 24.VII.1985/ Larochelle, Larivière”, M, CNCI

*Paracardiophorus humeralis* Fairmaire & Germain, P190, HDCU194, [handwritten] “CACHAGUA/ W. Aconcagua/ 18–IX–1974/ Col.P.VIDAL”, F, CNCI; TQ 1, HDCU215, [purple, partly handwritten] “Coll. R.I.Sc.N.B/ santiago/ ex. Coll. Mnizech” “Collection/ E. Candèze”; “(Bitactus cdz.)/ Humeralis/ Frm./ Chili. C. mn”; [partly handwritten] “Cardiophorus humeralis Fairm./ dét. E. Candèze”; [partly handwritten] “Paraplatynychus/ C.M.F. von Hayek/ det. 1960”; [handwritten] “cardiophorus/ humeralis/ no6i5”, F, ISNB

*Paracardiophorus subcruciatus* Carter, P189, HDCU193, “Australia, Qld. Mt./ Glorious 27°19'S,/ 152° 45'E, 1–5.XII.1997/ T. Hiller Malaise”, F, CNCI

*Paradonus pectoralis* (Say), P174, HDCU178, “USA, NY, Essex Co./ Crown Pt, splashing gravel beach under L. Champlain/ bridge, 19.VII.03 Hdouglas”, F, CNCI; P175, HDCU179, same data, M, CNCI; P176, HDCU180, “Canada, ON, Carleton,/ Crown Pt. 45°31’00”N–/ 76°08’00”W, 8.VI.03,/ sand/rock beach hand/ coll. J. Holland Leg.”, F, CNCI

*Phorocardius florentini* Fleutiaux, P056, HDCU060, “Tonkin/ Florentin”; “MUSÉUM PARIS/ Coll. E. FLEUTIAUX”, M, MNHN; P057, HDCU061, same data, F, MNHN

*Platynychus adjutor* Candèze, P001, HDCU001, [handwritten] “KU.79” “Japan./G.Lewis./1910–320., M, BM(NH); P002, HDCU002, on Card, under card [difficult to read] “Nowaba/ 6.80”; “DATA/ under card” Japan./ G. Lewis/ 1910–320.”, F, BM(NH); P003, HDCU003, “Yoyogi/ (Tokyo)/ 9–vi–1953/ A.Kato”; “9”; “Adjutor Cand/ ex Ôhira”, M, BM(NH)

*Paraplatynychus mixtus* Fleutiaux, P064, HDCU068, “MUSEUM PARIS/ HAUT-TONKIN/ CAO BANG/ CHRISSEMENT VILLAIN/ 1925”; [partly handwritten] “mixtus fleut./ FLEUTIAUX det.”; “paraplatynychus/ C.M.F. von Hayek/ det. 1965”, F, MNHN; P065, HDCU069, “Hué”; [handwritten] “Fl 200 mx”, M, MNHN; P066, HDCU070, “Hué”; [handwritten], M, MNHN

*Pronegastrius humeralis* (Candèze), P183, HDCU187, “Mimune(Mie)/ 1955–VI.18–21/ H.Ôhira.Coll”, F, CNCI; P184, HDCU188, same data; [handwritten] “Negastrius/ (Pronegastrius)/ humeralis/ Cand.”, F, CNCI

*Quasimus minutissimus* (Germar), P017, HDCU017, “Zbraslav/ Boh. C. VI53./ A. Olexa”; [handwritten] “Quasimus/ minutissimus/ Germ./ Det. Olexa ‘73”, M, CNCI; P018, HDCU018, same data, F, CNCI

*Rivulicola variegatus* Macleay, P021a, HDCU021, Specimen on left: [partly handwritten] “N.S. Wales/ Minto (W.W.F.)/ on/ apple trunk/ 16–VI–1903”, M, NHM; P021b, HDCU022, Specimen on right, same data, M, NHM; P022, HDCU023, [handwritten] “Kuranda/ 26–6–38/ C.G.C.”; [handwritten] “kuranda”; “A.E. Clarke/ Collection./ B.M.1957–24”; “♀ genitalia in/ water soluble resin/ Dimethyl Hydrantoin/ Formaldyhyde)”; [handwritten] “abdomen missing 19 III 85.”, F, NHM; P024, HDCU025, same data [handwritten] “Cryptohypnus/ variegatus Macl”, F, NHM

*Ryukyucardiophorus loochooensis* (Miwa), P271, “S. Ryukyu Is.:/ Ishigaki I./ Omoto-dake, 100-250m, 22.v.1964”; “Malaise Trap/ J.L. Gressitt”; “Paracardiophorus/ loochooensis/ Det H. Ôhira 1971”, F, BPBM; P272, same data, M, BPBM; P273, same data, M, BPBM

*Teslasena femoralis* Fleutiaux, P061, HDCU065, “MUSEUM PARIS/ GOYAZ à CUYUBA/ DE CASTELNAU 6–47”; [white disc, handwritten] “6/47”; [partly handwritten] “Teslasena/ FLEUTIAUX det.”, M, MNHN

*Triplonychus plagiatus* Erichson, P147, HDCU151, [handwritten] “35935”; [handwritten] “Brasil/ Puru”;”Fry Coll./ 1905.100.”; [handwritten] “35935=/ Brazil Para/ Higgins” [partly handwritten] “Triplonychus/ plagiatus/ Er. Det.Hdouglas 2005”F, NHM; P148, HDCU152, “Coll./ Janson.”; “Ega,/ Amazons./ Bates.” [partly handwritten] “Triplonychus/ plagiatus/ Er. Det.Hdouglas 2005”M, NHM

*Tropidiplus tellinii* Fleutiaux, [handwritten] P063, HDCU067, “Bir Donan/ 20.X.49”; [partly handwritten] “Tropidiplus/ telinii Fleut/ C.M.F.von Hayek det/ 1965”.F, MNHN

*Yukoana carinicollis* (Lewis), P191, HDCU195, [handwritten] “Mt. Mikuni/ 14–V–1966/ K.Baba”; [partly handwritten] “Yukoana/ carinicollis/ (Lewis, 1894)/ H.Ôhira Det. 1969”, M, CNCI; P192, HDCU196, [handwritten] “Kurokawa/ N. Echigo/ 21.–V, 1967/ K. Baba”; [handwritten] “Y. carinicollis/ Det. Ôhira 1967”, F, CNCI

## APPENDIX III

### Type specimens

Label data from type specimens of species coded for phylogenetic analyses. Entries include information in the following order: Type code (number attached to photos), scientific name, kind of type specimen (A = allotype, H = holotype, L = lectotype, N = neotype, P = paratype, PL = paralectotype, S = syntype, T = type of unknown kind), label data and any lectotype designation (text from multiple labels, beginning with uppermost label, are listed in a separate set of quotation marks, separated by semi-colons. Text from each line of a label with multiple lines of text is separated by a “/”. Notes about the appearance of a label appear in square brackets before quotation), the sex of the specimen (M/F), and the coden representing the specimen depository.

T001, *Cardiodontulus brandti* Van Zwaluwenburg, H, “NEW GUINEA: PAPUA/ Kiunga, Fly River/ IX–10–17–1957”; “Wm. W. Brandt/ Collector”; “♂”; [red] “Holotype”; “Cardiodontulus/ brandti/ ♂/ Van Zwal./ Holotype/ No.”; [pink] “HT–6047/ BISHOP MUSEUM”, M, BPBM

T002, *Cardiodontulus brandti* Van Zwaluwenburg, P, [genitalia in microvial, and left wing and urosternites 3–7 mounted on a card] “NEW GUINEA: PAPUA/ Kiunga, Fly River/ IX–10–17–1957”; “Wm. W. Brandt/ Collector”; “♂”; [yellow] “Paratype”; “Cardiodontulus/ brandti/ ♂/ Van Zwal.”, M, BPBM

T005, *Cardiophorus inflatus* Candèze, L, Lectotype designated here. [handwritten] “Mantchouria”; “Collection/ E. Candèze”; [handwritten] “n.sp./ inflatus/ Cdz/ Mandchuria”; [partly handwritten] “Cardiophorus/ inflatus cd./ dét. E. Candèze”; “♀”; [partly handwritten] “Holotype. Cardiophorus inflatus/ C.M.F.von Hayek. Cand/ det., 1957”; [red] “Holotype”; and with the author’s red designation label “LECTOTYPE/ Cardiophorus/ inflatus/ Candèze desig./ Douglas, 2006” [with ♀ genitalia on slide on pin]. Label mailed separately to ISNB in 2006., F, ISNB

T008, *Agrypnella eburnea* Champion, H, [circular with red margin] “TYPE”; [handwritten] “Santarem”; “Coll./ Janson”; “Janson coll./ 1903–130.”; [handwritten] “Agrypnella/ eburnea, Ch./ type”, M, NHM

T009, *Cardiophorus mirabilis* Candèze, L, Lectotype designated here. [circular with blue margin] “SYN/ TYPE”; [handwritten] “Cardiophorus/ mirabilis/ Inde or. Cdz”; and with the author’s red designation label “LECTOTYPE/ Cardiophorus/ mirabilis/ Candèze desig./ Douglas 2015”, ?, NHM

T010, *Triplonychus trivittatus* Champion, L, Left hand specimen on card labeled: [disk with blue border] “SYN_/ TYPE”; “Tolé,/ Panama./ Champion.”; “♂”; [partly handwritten] “B.C.A.Coll.III[1]./ Triplonychus/ trivittatus/ Ch.”; [red label, partly handwritten] “LECTOTYPE Spcm on/ left/ Triplonychus/ trivittatus/ Champion/ desig. P.J. Johnson 2001”, M, NHM

T011, *Triplonychus trivittatus* Champion, PL, Right hand specimen [formerly left] on card labeled: [disk with blue border] “SYN_/ TYPE”; [disk with red border] “TYPE”; “Bugaba/ 800–1500 ft./ Champion”; “♂”; [partly handwritten] “B.C.A.Coll.III[1]./ Triplonychus/ trivittatus/ Ch.”, M, NHM

T012, *Elater thoracicus* Fabricius, PL, Pin unlabeled, in box with card: “Elater thoracicus F./ 2 syntypes/ UNIVERSITES/ ZOOLOGISKE MUSEUM/ UNIVERSITETSPARKEN 15/ DK–2100 KØBENHAVN Ø”, ?, ZMUC

T013, *Elater thoracicus* Fabricius, L, Lectotype designated here. Pin unlabeled, in box with card: “Elater thoracicus F./ 2 syntypes/ UNIVERSITES/ ZOOLOGISKE MUSEUM/ UNIVERSITETSPARKEN 15/ DK–2100 KØBENHAVN Ø”; and with the author’s red designation label “LECTOTYPE/ Elater/ thoracicus/ Fabricius desig./ Douglas 2015”, ?, ZMUC

T016, *Oedostethus femoralis* LeConte, L, Lectotype designated here, [green disc without writing]; [red label, partly handwritten] “Type/ 2461”; [handwritten] “Oedostethus/ femoralis/ Kansas. Lec.” “; and with the author’s red designation label “LECTOTYPE/ Oedostethus/ femoralis/ LeConte desig./ Douglas 2015”, M, MCZC

T017, *Eniconyx pullatus* Horn, L, Lectotype designated here. “Ari”; [partly handwritten red] “LectoTYPE/ 3405”; [HW] “Eniconyx/ pullatus/ Horn”; [partly handwritten, red] “MCZ/ SynType/33774”; and with the author’s red designation label “LECTOTYPE/ Eniconyx/ pullatus/ Horn desig./ Douglas 2015”, M, MCZC

T018, *Eniconyx pullatus* Horn, PL, “Ari”; [partly handwritten blue] “PARA-TYPE/ 3405”; and with the author’s orange label “PARALECTOTYPE/ Eniconyx/ pullatus/ Horn label/ Douglas 2015”, M, MCZC

T020, *Aphricus californicus* LeConte, H, [gold disc without writing]; [red] “Type./ 2623”; [handwritten] “Aphricus/ californicus/ S.D. Lec.”, M, MCZC

T024, *Lesnelater madagascariensis* Fleutiaux, L, Lectotype designated here. “Madagascar; Ambohibeloma.”; [handwritten] “Pachyelater/ madagascariensis/ ♂ presummé, type/ syn./ P. Lesne vid. 1906”; MUSÉUM PARIS/ 1952/ COLL A. OBERTHUR”; and with the author’s orange label “LECTOTYPE/ Lesnelater/ madagascariensis/ Fleutiaux label/ Douglas 2015”, M, MNHN

T026, *Pachyelater madagascariensis* Lesne, PL, “Madagascar; Ambohibeloma.”; [handwritten] “Pachyelater/ madagascariensis/ P. Lesne vid. 06”; MUSÉUM PARIS/ 1952/ COLL A. OBERTHUR” [with 6 antennomeres attached mounted on card attached to pin]. Abdomen on card, genitalia on separate glass slide HD–01, F, MNHN

T027, *Pachyelater madagascariensis* Lesne, PL, “Madagascar; Ambohibeloma.”; [handwritten] “Pachyelater/ madagascariensis/ Lesne/ P. Lesne vid. 06”; MUSÉUM PARIS/ 1952/ COLL A. OBERTHUR”. Abdomen on card, genitalia on separate glass slide HD–02, F, MNHN

T028, *Cardiophorellus gracilicornis* Cobos, P, “Odzala/ Congo/ Octobre”; “MUSÉUM PARIS/ MISSION/ A. DESCARPENTRIES/ ET. A. VILLIERS/ 1963–1964”; [red] “PARATYPUS/ A. COBOS”, M, MNHN

T029, *Cardiophorellus gracilicornis* Cobos, P, “Odzala/ Congo/ Octobre”; “MUSÉUM PARIS/ MISSION/ A. DESCARPENTRIES/ ET. A. VILLIERS/ 1963–1964”; [red] “PARATYPUS/ A. COBOS”, M, MNHN

T033, *Phorocardius solitarius* Fleutiaux, L, Lectotype designated here. [handwritten] “Tonkin/ Zhan Moi/ Vitalis Juin ##”; [red] “TYPE”; “MUSÉUM PARIS/ Coll. E. FLEUTIAUX”; [partly handwritten] “Phoroc/ solitarius/ Fleut. Type/ COLLECTION FLEUTIAUX”; [handwritten] “griffes très/ __lement bifides/4’ art, tarses/ tronqué/ carrément_”; and with the author’s red designation label “LECTOTYPE/ Phorocardius/ solitarius/ Fleutiaux desig./ Douglas 2015”. Body on card with abdomen, genitalia on separate glass slide, HD–03., F, MNHN

T034, *Blaiseus bedeli* Fleutiaux, L, “RÉG. DE LUC-NAM/ (TONKIN) L.BLAISE”; “MUSEUM PARIS/ (COLL. PH.FRANÇOIS)/ COLL. L. BEDEL 1922”; “TYPE”; [partly handwritten] “Blaiseus/ Bedeli Fleut./ type/ FLEUTIAUX det.”; “LECTOTYPE/Blaiseus/ bedeli/ Fleutiaux desig./Douglas 2006”, M, MNHN

T035, *Cardiophorus florentini* Fleutiaux, L, Lectotype designated here. “Tonkin/ Florentin”; [red] “Type”; “MUSÉUM PARIS/ Coll. E. FLEUTIAUX”; [handwritten] “Cardiophorus/ Florentini/ Fleut. Type”; [partly handwritten] “Fleut., Ann./ Soc. Ent. Fr./ 1894. P. 687/ Collection FLEUTIAUX”; [partly handwritten] “C. Florentini/ Fleut. type/ Collection FLEUTIAUX”; and with the author’s red designation label “LECTOTYPE/ Cardiophorus/ florentini/ Fleutiaux desig./ Douglas 2015”, ? , MNHN

T036, *Aphricus chilensis* Fleutiaux, L, Lectotype designated here. “TYPE”; [handwritten] “chili/ coll. Vienna”; [partly handwritten] “aphricus/ chilensis/ type/ COLLECTION FLEUTIAUX”. Head and thorax in capsule on separate pin labeled: [handwritten] “found loose/ in box/ CHM. 3.81”; and with the author’s red designation label “LECTOTYPE/ Aphricus/ chilensis/ Fleutiaux desig./ Douglas 2015”, M, MNHN

T037, *Globothorax chevrolati* Fleutiaux, L, Lectotype designated here. “Type”; “Collection Chevrolat”; [handwritten] “Globothorax/ chevrolati/ Fleut. type/ Bresil”; [partly handwritten] “Globothorax/ chevrolati/ Fleut. type/ Bresil/ Collection FLEUTIAUX”; [partly handwritten] “Fleut. E.R./ Soc. Ent. Belge/ 1891 p233/ Collection FLEUTIAUX”; and with the author’s red designation label “LECTOTYPE/ Globothorax/ chevrolati/ Fleutiaux desig./ Douglas 2015”, F, MNHN

T038, Cardiophorus (Perrinellus) angustatus Fleutiaux, T, “MAHATSINJO/ près Tananarive”; [handwritten] “Mahatsinjo/ Près Beforona”; “Type”; [partly handwritten] “Cardiophorus/ s.g. Perrinellus/ angustatus/ type/ Collection FLEUTIAUX”, M, MNHN

T039, *Anelastes femoralis* Lucas, L, Lectotype designated here. “MUSEUM PARIS/ GOYAZ À CUYUBA/ DE CASTELNAU 6–47”; [handwritten] “anelastes/ femoralis Lucas”; [handwritten] “type”; [partly handwritten] “type de/ Lucas/ FLEUTIAUX det.” “; and with the author’s red designation label “LECTOTYPE/ Anelastes/ femoralis/ Lucas desig./ Douglas 2015”, M, MNHN

T040, *Tropidiplus tellinii* Fleutiaux, L, Lectotype designated here. [handwritten] “Cheren/ Eritrea XI”; [partly handwritten] “Syntype/ Tropidiplus/ tellini Fleut. 1903./ C.M.F.von Hayek det/ 1964”; [partly handwritten] “Tropidiplus/ tellinii Fleut/ C.M.F.von Hayek det/ 1963”; and with the author’s red designation label “LECTOTYPE/ Tropidiplus/ tellinii/ Fleutiaux desig./ Douglas 2015”, M, MNHN

T041, *Platynychus indicus* Motschulsky, L, Lectotype designated here. [handwritten] “Platynychus/ indicus/ Motsch./ Ind.or”; [handwritten] “Ind. Or.”; [red rectangle]; and with the author’s red designation label “LECTOTYPE/ Platynychus/ indicus/ Motschulsky desig./ Douglas 2015”, F, ZMUM

T042, *Cardiotarsus vitalisi* Fleutiaux, L, Lectotype designated here. [handwritten] “Pnom Penh/ Vitalis”; “Type”; [partly handwritten] “Cardiotarsus/ Vitalisi Fleut./ type/ Ann. Soc. Ent. Fr. 1918/ Collection FLEUTIAUX”; and with the author’s red designation label “LECTOTYPE/ Cardiotarsus/ vitalisi/ Fleutiaux desig./ Douglas 2015”; [with part of antennae on card], ?, MNHN

T043, *Athous argentatus* Abeille de Perrin, L, Lectotype designated here. [handwritten] “Jaffa/ bic”; [handwritten] “athous/ argentatus/ ab.”; and with the author’s red designation label “LECTOTYPE/ Athous/ argentatus/ Abeille de Perrin desig./ Douglas 2015”, M, MNHN

T044, *Patricia australica* Van Zwaluwenburg, P, “SirGraham/ MooreID/ Waustralia”; “20Feb1945/ Bmalkin”; “♂”; [partly handwritten] “Patricia australica/ ♂ Van. Z”; [yellow, partly handwritten] “PARATYPE/ CNCI No. 13633”, M, CNCI

T045, *Buckelater argutus* Costa, P, [handwritten] “Barueri/ SP, Brasil/ 21.XI.1955/ K.Lenko col./ 7270”. Wing on microslide labeled: [handwritten]: “Costa, 73”; [red, partly handwritten] “Buckelater/ argutus/ paratipo/ C Costa det 1973”, M, MZSP

T046, *Buckelater argutus* Costa, P, [handwritten] “Barueri/ SP, Brasil/ 21.XI.1955/ K.Lenko col./ 7270”. Legs and antennae on microslide labeled: [handwritten]: “Costa, 73”; [red, partly handwritten] “Buckelater/ argutus/ paratipo/ C Costa det 1973”, M, MZSP

T047, *Buckelater argutus* Costa, P, Body in microvial, labelled: [partly handwritten] “K. Lenko-Leg.”; [handwritten] “21.XI.1955/ Barueri/ S. Paolo/ 7270”. Mouthparts and genitalia on 2 microslides labeled: [handwritten]: “Costa, 73”; [red partly handwritten] “Buckelater/ argutus/ paratipo/ C Costa det 1973”, M, MZSP

T048, *Buckelater argutus* Costa, P, [head thorax and abdomen on point]; [handwritten] “Ponta Grolla/ XII–1938/ Camargo col”. Mouthparts and genitalia on microslides labeled: [handwritten]: “Costa, 73”; [red, partly handwritten] “Buckelater/ argutus/ paratipo/ C Costa det 1973”, M

T049, *Buckelater argutus* Costa, P, [point-mounted]; [handwritten] “Serra Caraça–1380m/ MG–Brasil XI–961/ Kloss, Lenko,/ Martins & Silva col.”; [red, partly handwritten] “Buckelater/ argutus/ paratipo/ C Costa det 1973”, M, MZSP

T050, *Horistonotus flavidus* Candèze, L, Lectotype designated here. [purple, partly handwritten] “Coll. R. I. Sc. N. B./ Bresil NOVO FRIBURGO”; “Collection/ E. Candèze”; [handwritten] “Flavidus/ N.Frib. cde”; [partly handwritten] “Horistonotus/ flavidus Cd./ dét. E. Candèze”; and with the author’s red designation label “LECTOTYPE/ Horistonotus/ flavidus/ Candèze desig./ Douglas 2015”, F, ISNB

T051, *Horistonotus flavidus* Candèze, PL, [purple, partly handwritten] “Coll. R. I. Sc. N. B./ Bresil NOVO FRIBURGO”; “Collection/ E. Candèze”; [partly handwritten] “Horistonotus/ flavidus Cd./ dét. E. Candèze”; and with the author’s orange label “PARALECTOTYPE/ Horistonotus / flavidus / Candèze label/ Douglas 2015”, F , ISNB

T052, *Nyctor expallidus* Semenov-Tian-Shanskij, H, [partly handwritten] “Nyctor expallidus/ Typ. ♂. M./ A. Semenov-Tian-Shansky det.V.22”; [red disk]; [hand written, approx.] “Farad. / Sumakov / [red] “Holotypus”, M, ISNB

T053, *Nyctor expallidus* Semenov-Tian-Shanskij, P, [handwritten, approx.] “ Farad/ Sumakov 1907”/ [red] “Paratypus”, M, ZMAS

T054, *Nyctor expallidus* Semenov-Tian-Shanskij, A, “Nyctor expallidus/ m. Typ.♀/ A. Semenov-Tian-Shansky det. V.22”; “Remedek 12.VI.1905/ _. [illegible, probably U. Beckmann]”; [red] “Allotypus”, F, ZMAS

T055, *Neocardiophorus mamajevi* Gurjeva, P, [handwritten, approx.] “Kizipkut / 2. Kuldmzhuk-Tau / Mamaev 20.III.961”; [handwritten, abbreviation for “sandy ridge”] “Peschan. Gora.”; [red, partly handwritten] “Paratypus/ Neocardiophorus/ mamajevi Gurjeva”, M, ZMAS

T056, Cardiophorus (Metacardiophorus) sogdianus Gurjeva, P, [handwritten, approx.] “Guzar, Peidzhi-keetk ob/ Bromstein S.V. 956”; [red, partly handwritten] “Paratypus/ Cardiopho-/ rus (metacardiophorus)/ sogdianus Gurjeva”, M, ZMAS

T058, *Gastrimargus schneideri* Schwarz, L, Lectotype designated here. [body and ovipositor on card] “Africa or./ Micindani/ EX COLL. F. SCHNEIDER”; “Coll.Schwarz”; “Dtsch.Entomol./ Institut Berlin”; [red] “LECTOTYPE ♀”; [handwritten, glued onto next label] “Margogastrius/ Schneideri/ Schw.”; [handwritten on blue label, partly covered] “Schneideri/ Schw.”; [partly handwritten] “Margogastrius/ schneideri/ Schwarz/ C. Girard vid. 1974”; [partly handwritten] “S/F. !?/ Cardiophorinae/ C. Girard det. 1974”; and with the author’s red designation label “LECTOTYPE/ Gastrimargus / schneideri/ Schwarz desig./ Douglas 2006”, F, DEIC

T059, *Gastrimargus schneideri* Schwarz, PL, “Africa or./ Micindani/ EX COLL. F. SCHNEIDER”; “Coll.Schwarz”; “Dtsch.Entomol./ Institut Berlin”; [handwritten] “Margogastrius/ Schneideri/ Schw.”; [red] “PARALECTOTYPE ♀”; “Margogastrius/ schneideri/ Schwarz/ C. Girard vid. 1974”; [partly handwritten] “S/F. !?/ Cardiophorinae/ C. Girard det. 1974”; and with the author’s orange label “PARALECTOTYPE/ Gastrimargus/ schneideri/ Schwarz label/ Douglas 2006”, F, DEIC

T060, *Craspedostethus rufiventris* Schwarz, L, Lectotype designated here. [green, handwritten] “Kameroun”; “Coll. Schwarz”; [red] “Lectotypus”; [handwritten] “rufiventris/ Schw.”; “Dtsch.Entomol./ Institut Berlin”; [partly handwritten] “Lectotype/ Craspedostethus/ rufiventris Schw./ C.M.F. von Hayek/ det. 1963”; “DEI/ Eberswalde”; [handwritten] “Craspedostethus/ rufiventris/ Schw.”; and with the author’s red designation label “LECTOTYPE/ Craspedostethus/ rufiventris/ Schwarz desig./ Douglas 2015”, F, DEIC

T061, *Craspedostethus rufiventris* Schwarz, PL, [green, handwritten] “Kameroun/ Kraals”; “Coll. Schwarz”; [red] “Paralectotypus”; [handwritten] “Dtsch.Entomol./ Institut Berlin”; [partly handwritten] “Paralectotype/ Craspedostethus/ rufiventris Schw./ C.M.F. von Hayek/ det. 1963”; “DEI/ Eberswalde”; and with the author’s orange label “PARALECTOTYPE/ Craspedostethus/ rufiventris/ Schwarz label/ Douglas 2015”, F, DEIC

T062, *Cardiophorus convexulus* LeConte, L, “#1157 Maine”, “convexulus”, “true type of convexulus J.C. Brimley”; “LECTOTYPE/ Cardiophorus/ convexulus/ LeConte, desig./ Douglas 2002”, F, MCZC

T063, *Elater convexa* Say, N, “ILL” “convexus/ (Say) fide Walsh”; “NEOTYPE/ Elater convexa/ Say Desig./ Douglas 2002”, M, MCZC

T064, *Cardiophorus togatus* Horn, L, “Tex”; “female” (symbol); “LectoType 3331”; “C. togatus Horn” “MCZ type 33727”; “LECTOTYPE/ Cardiophorus/ togatus/ Horn, desig./ Douglas 2002”, F, MCZC

T065, *Cardiophorus luridipes* Candèze, L, Lectotype designated here. [blank square]; [green, handwritten] “Calif/ Mufeiim”; “405.”; [handwritten] “Cardiophorus luridipes”; “Collection/ Chevrolat”; [partly handwritten] “typique Cand./ Mon./ Collection Fleutiaux”; [red] “Type”; and with the author’s red designation label “LECTOTYPE/ Cardiophorus/ luridipes/ Candèze desig./ Douglas 2015” , F, MNHN

T069, *Elater sputator* Linnaeus, T, [Handwritten] “I5 Sputator”; “24”, ?, LSUK

T070 to T072, *Elater sputator* Linnaeus, T, unlabeled, ?, LSUK

T073, *Elater sputator* Linnaeus, L, Lectotype designated here with the author’s red designation label “LECTOTYPE/ Elater/ sputator/ Lin. desig./ Douglas 2015”, ?, LSUK

T074, *Elater cardisce* Say, N, [pink disc = Middle States: Maryland; Delaware; New York; New Jersey; Pennsylvania; Connecticut?; Rhode Island?]; “C. cardisce/ Coney Is./ Say”; “NEOTYPE/ Elater/ cardisce/ Say, desig./ Douglas 2002”, M, MCZC

T075, *Cardiotarsus capensis* Candèze, L, Lectotype designated here. [green, handwritten] “Cardiotarsus/ capensis mini./ L. ad Cap. Bon. Sp. D. -at”; [handwritten, partly illegible] “T__ge”; [handwritten] “Cardiotarsus/ capensis Cdze/ see Candèze”; “Janson coll./ ex Dejean./ 1903–130.”; [handwritten] “dr 314”; [partly handwritten] “♀ genitalia/ see slide Coll./ TYPE.”; and with the author’s red designation label “LECTOTYPE/ Cardiotarsus/ capensis/ Lacordaire desig./ Douglas 2015” , F, NHM

T076, *Esthesopus castaneus* Eschscholtz, L, Lectotype designated here. [handwritten] “Castaneus./ E./ Brasilia./ Rio Jan.”; [handwritten] “Esthesopus”; and with the author’s red designation label “LECTOTYPE/ Esthesopus/ castaneus/ Eschscholtz desig./ Douglas 2015”, F, ZMUM

T077, *Cardiophorus musculus* Erichson, L, Lectotype designated here. [handwritten] “165.”; [handwritten] “16599”; [microslide with handwritten label] “♀ genitalia/ musculus Er.”; [red] “Lektotypus/ Nr.”; [partly handwritten] “Lectotype/ Cardiophorus/ musculus Er./ C.M.F. von Hayek./ det. 1960”; [handwritten] “musculus/ Er./ Blankherb. Kn./ Austr. Dahl”; and with the author’s red designation label “LECTOTYPE/ Cardiophorus/ musculus/ Erichson desig./ Douglas 2015”, F, ZMHB

T078, *Cardiophorus musculus* Erichson, PL, “♀”; [handwritten] “16599”; [red] “Paratypus/ Nr.”; [partly handwritten] “Cardiophorus/ musculus Er./ C.M.F. von Hayek./ det. 1960”; “Hist.-Coll. (Coleoptera)/ Nr. 16599/ Cardiophorus musculus Er./ Austria, Dahl/ Zool. Mus. Berlin”, F, ZMHB

T079, *Cardiophorus musculus* Erichson, PL, “♂”; [handwritten] “gibbicollis/ Mg.s.Dhl”; [handwritten] “16599”; [red] “Paratypus/ Nr.”; [partly handwritten] “Cardiophorus/ musculus Er./ C.M.F. von Hayek./ det. 1960”; “Hist.-Coll. (Coleoptera)/ Nr. 16599/ Cardiophorus musculus Er./ Austria, Dahl; Zool. Mus. Berlin”, M, ZMHB

T080, *Elater cinereus* Herbst, L, Lectotype designated here. [female genitalia on card]; “♀”; [handwritten] “16647”; [handwritten] “Berlin”; [handwritten] “cinereus/ Hbt Larch. Er./ pilosus Payk./ Equiseti Gyll”; “Hist.-Coll. (Coleoptera)/ Nr. 16641/ Cardiophorus cinereus Hrbst./ Berolin; Zool. Mus. Berlin”; [partly handwritten] “Dicronychus/ cinereus (Herbst)/ J.Chassain det. 82”; and with the author’s red designation label “LECTOTYPE/ Elater/ cinereus/ Herbst desig./ Douglas 2015”, F, ZMHB

T081, *Elater minutissimus* Germar, L, Lectotype designated here. “17525”; [handwritten, partly illegible] “minutissimus/ Germ. Faun./ des ?? Sp?????”; [handwritten] “minutis/ firmus/ Tirol/ Italia”; “Hist.-Coll. (Coleoptera); Nr. 17525; Cryptohypnus minutissimus/ Germ./ Italia-Tyrol/ Zool. Mus. Berlin”; and with the author’s red designation label “LECTOTYPE/ Elater/ minutissimus/ Germar desig./ Douglas 2015”, ?, ZMHB

T082, *Elater demustoides* Herbst, T, “17509”; [handwritten] “Europa”; [handwritten] “dermestoides/ Herbst”; [red] “LECTOTYPE ♂/ L.Leseigneur 1968”; [partly handwritten] “Zorochrus/ dermestoides Hbst/ Leseigneur det. 1968”. With previously dissected ♂ genitalia on glass slide labeled: [handwritten] “Zorochrus/ dermestoides/ Herbst/ genitalia ♂”; [red] “LECTOTYPE ♂/ no 17509/ L.Leseigneur 1968”, M, ZMHB

T083, *Triplonychus acuminatus* Candèze, L, Lectotype designated here. [male genitalia on card attached to pin]; [circular with blue margin] “SYN-/ TYPE”; [circular with red margin] “Type”; “Type”; “Cayenne.”; [handwritten, green] “LacorDaire”; [green card]; “Coll.Janson/ Ex Laferte”; [handwritten, green] “Cayennensis var/ Cayenne. Bugt.”; [handwritten] “H. acuminatus/ CDZ”; [handwritten] “Triplonychus/ acuminatus/ Type Cdze”; and with the author’s red designation label “LECTOTYPE/ Triplonychus/ acuminatus/ Candèze desig./ Douglas 2015”, M, NHM

T088, *Horistonotus simplex* LeConte, L Lectotype designated here. [green disc]; [red, partly handwritten] “Type 2399”; [handwritten] “Horistonotus/ simplex Lec; and with the author’s red designation label “LECTOTYPE/ Horistonotus/ simplex/ LeConte desig./ Douglas 2015”.”, M?, MCZC

T090, *Esthesopus parcus* Horn, L, Lectotype designated here. [handwritten] “E./ parcus/ Horn”; “LectoTYPE/ 3345”; [red] M.C.Z./ Type/ 33734”; “Ariz”; and with the author’s red designation label “LECTOTYPE/ Esthesopus/ parcus/ Horn desig./ Douglas 2015”, F, MCZC

T092, *Cebrio speratus* Fall, H, [partly handwritten] “Hope Ark/ 6/18/26”; [partly handwritten] “Type/ speratus”; [red] “M.C.Z./ Type/ 24339”; “H.C. Fall/ COLLECTION”, M, MCZC

T093, *Cardiophorus adjutor* Candèze, L, Lectotype designated here. [circular with blue margin] “SYN-/ TYPE”; “Japan./ G. Lewis./ 1910–320”; [partly handwritten] “SYNTYPE/ Cardiophorus/ adjutor Cand 73/ C.M.F. von Hayek/ 1985 O.T.O”; and with the author’s red designation label “LECTOTYPE/ Cardiophorus/ adjutor/ Candèze desig./ Douglas 2015”, F, NHM

T094, *Platynychus mixtus* Fleutiaux, L, Lectotype designated here. “Hué”; [partly handwritten] “mixtus Fleut/ Type/ Fairmaire Cand./ pars–Fleut. 1889/ Collection FLEUTIAUX”; [red] “TYPE”; [handwritten] “Fl 201 mx”; [partly handwritten] “♀ genitalia/ See slide Coll./ No. TYPE”; [partly handwritten] “Paraplatynychus/ C.M.F. von Hayek/ det. 1965”; ♀ genitalia removed to glass microslide labeled: [partly handwritten] “mixtus Fleut./ type/ fairmairei Cand/ pars Fleut 1889/ Hué”; and with the author’s red designation label “LECTOTYPE/ Platynychus/ mixtus/ Fleutiaux desig./ Douglas 2015”, F, MNHN

T095, *Coptostethus americanus* Horn, H, [handwritten] “Coptostethus/ americanus/ Horn”; [red] “HoloTYPE/ 3335”; [red] M.C.Z./ Holotype/ 33729; “La.”, ? , MCZC

T096, P170, HDCU174, *Blaiseus nothoafricanus* Douglas, H, “Lydenburg dist./ Transvaal”; “Janson coll./ 1903–130.”; [red] “HOLOTYPE/ Blaiseus/ nothoafricanus/ Douglas 2006” (NHM), M, NHM

T097, P171, HDCU175, *Blaiseus nothoafricanus* Douglas, P, [handwritten] “Ayres”; [handwritten] “P.B. Spei/ Transvaal”; “Fry Coll./ 1905–100”; [yellow] “PARATYPE/ Blaiseus/ nothoafricanus/ Douglas 2006,” with genitalia in microvial attached to pin, M, NHM

## APPENDIX IV

### Phylogenetic matrix

0000000000000000000000000000000000000000000000000000000000000000000000000000000000000000000000000001111111111111111111111111111111111111111111111111111111111111111

0000000001111111111222222222233333333334444444444555555555566666666667777777777888888888899999999990000000000111111111122222222223333333333444444444455555555556666

1234567890123456789012345678901234567890123456789012345678901234567890123456789012345678901234567890123456789012345678901234567890123456789012345678901234567890123

Adrastus

000001001000?10000220000001020210100001000000001110100102100010011000011000001011001201011001002001000000102101110100?000131000000010??10100?000?00????0??0?0???010

Agriotes

010000001000?00000011000001020210100101101000010112000102001100011000010000010001000101000001000011002000002001110000?10000?000100110??01?10??10?00????20?0?0???010

Agrypnel

010001001010010000010011202120000101001111010002101011102012110121100111001000?1????00000000000011100100000000100011201000200200010?0?????00?00??00???????0?????000

Agrypnus

010001001000?00000001010001100110100200100000010112100112012010001000010000010010000101011011010001000000102?00000100?1000310000011010?0??10?011000????0??0?0???110

Aphricus

00010000121011001000100000?10??10000000001010011102110100012000011010010000001110000101111000000101000000100?00100102010002000???????????????????????????????????1?

AphriChi

00010101101001001011100000?10??100000000110100121?2000102100010011010010000001110101101010000000001000000001?00100102000013000?????????????????????????????????????

AphricNZ

01110101120001000001000000?11??10010001000010012102100100112100011000010000001110000100010000000?01000000100101101112010013000?????????????????????????????????????

AptopusA

000000021210010011000000000111100100001111001012101000000012100001001000001000010100101010000003100000000100???100102000002000?????????????????????????????????????

AptopusP

0001000010100100110000000001210001000000000100121010000000121100010000000010000101001010100000021000000000021010001020100020001000000?0101000100?00???1100011210110

Arhaphes

000001001010010000431000001200101101011100000010113210100012010011000010001000110??1000001001000001000000000101000102000012000??000010??1111?000?01101?0??0?0???000

Athous__

010001001000010010001000000200120100000100000001112000102000110011000010000010001000100011001400000000000100121110000?1000310000001122?001000211010????0??0?0???011

Berninel

000001021010000010001000001020110100001111000010112000102001010011000010000100001000000010000000101000000102001001100?00010?1000000120?00100?001200????0??0?0???000

BlaiseuB

00000000020101001022100000?10??10100001000110111120000102012000001010000000011010011111010000200001000010000001101102000113?01?????????????????????????????????????

BlaiseuT

00000010000101001022100000?10??0000000000001011110200010211201001101001000000101001?00001000000000100??10???021100002000103?01?????????????????????????????????????

Buckelat

000100002000?1000000000000?12??001000010100010111010000001121000210010010010011101?1101000001200000000100002?00101112000002000?????????????????????????????????????

Cardiodo

00010101101001001100000000?12??00100001010001011101000000012100001000001001010010000101000000201100000000001101101112000002010?????????????????????????????????????

Cardiohy

010001001010010010011010001121000101001100000011111010100012101101000110001001?1???1000000100000111001000002?0000110200001200201000000?01110?000?00????0??0?0???000

Cardphrl

00000000000101001001000000111100000000000010001110000000001200000100000000100001???1101010000000110000000100101100112010012000?????????????????????????????????????

CardAngu

000000001010010010?00000001121000000001000010012000000000012000011000000001001010101100010000000001000000000001000002010012010?????????????????????????????????????

CardPerr

00010000100001001000000000?1???00100000000?100?210200??0?012010011???00000?011?????????010000000??000???0?0?????????2010002000?????????????????????????????????????

CardBrev

0000000110000000100000010011210001000010000000121000001000121000010000100010010101011010100000010010000001001011001020100120010001000?0100000100?00????1000111000?0

Coptoste

00000?00101001001000000000111100010000100000001200100000011210000100000001?0???????????0100000010000000000001010001020100020001100?00?0000000100?00???11001111?0110

CardCard

0000000010100100100010001011210101000010011000121000000000121000110001100010110101001010000010001010001000020000001021000120101100000?1000000100?00???1100000???210

CardCvxl

0000000110000100100010000011210001000000000000120010000000121000010000000010010101011010100000001000000000020010001020100120001001100?1000000100?00???1100001001010

CardCvxs

0000000010100100100000000011210001000010000000121014000000121000010000000000110101001010100000001000000000001010000020100020000000100?0100000100?00???1100001000010

CardGram

0000000020110100100000010011210001000000000000120000000001120000010000000000010101011010000000000000000000021010001020100120000100100?0100000100?00???0100011101010

CardInfl

0001000?1000010010000000001121000100001?10000012000000000?1200000100000000?000??0??????000000000??????????????????????????????000010???????????0?00???1100001001010

CardLuri

000000001010010010001000101121010100000000001012100000000012100011000010001001010101101000000000101000100000100000002031012?000100000?1000000100?00???0100000???2?0

Zygocard

000100002000000010001000001111000100001000000012100000000012000011000011101001110??11010100000000010001000001010001021000120100100100?1000000100?00???11000111?01?0

Metacard

?00?0000000001000001100000?11??10100001000010012100000100012100011000010001001210101101010000000001000100000101100102000012000?????????????????????????????????????

CardspC_

0000000110000100100010000111210001000010000010121010000000120000010000000000000101001010000000001100000000021010001020000120010000000?0000000100?00???1100000???0?0

CardToga

0000000110000100100010000011110001000000000000121000000000120000010000000010110101011010100000001000000000001010001020100020001100100?100?000100?00???1100001101010

CartCape

0001010110000100100000000011210001000010000010121010000000120000010000000000000101001010000002000000000000001011001020000020000001100?0000001100?00???11000110110?0

CartMjob

01010101121001001100100000?12??001000011000010121010000001121000010000000010002101011010100002011000000000011011001020000020000000100?0001000200?10???0101000???010

CartPhil

00000000100001001000000000?12??001000010000000?2100000000012100001000000000001?100001010110002000010000000001010??1020100020001000100?0??????100?00???11100110110?0

Craspedo

00000100121001001100000000112110010000101100101110100000001210000100000100100001010110100000000010000?000000101001102000002001?100100?????10???0?00????0??0?0???0?0

Dicronyc

0000000110100100100010000011210001000010000000120000000000121000010000000010000101001010100000011000001000001010001020100120000001100?1000000100?00???11000010110?0

Displaty

00000001101001001100?0000011210001000010111010120010000000121000010000000010010101001010000000?11000000000001011000020100020101100100?0000000100?00???1100010???1?0

ElaterFe

001001000000?00000001001001000110100000101000010012100102000000011000010000000001000111001000000001002000100001100100?0000320000011010?00101?011000????00?0?0???010

Esthesop

00000001101101001100000000?11??0010000100010101010100000001210000101000000100011010?1010000012011100050001001011001020000120000100100?1?01000100?10????100000???010

EsthParc

00000010100001001100000000?00??001000010111010101010010001121010010000000010000100011010100012011000050000021210001120000020100000100?0100000100?00???11?30?1001010

Fleutiau

0000100010100100101111000011001101010000110000111120101020120000110000100010000100001000100010000010001000101011011020000131000100000??101001100?01000?0??0?0???0?0

Globotho

????00?020000100??11100000?01??001000000000111101001000000121000010010100?100101010010101000?00110100?????????????????????????0001100???????0??0?00???0102000???0?0

Horiston

00000001101101001100000000011??00100001111001011101000000012000001000000001001110101111000000001110000000000101101102000012000010010???????????0?10???0100000???010

HoriSimp

00000001101001001000000000011??001000010110010121010000000121000010000100010010110001110100000011000000001021011001120000120000000000?0001000200?10???0100000???010

Hypnoidu

01000100101000001000101000102011010100111100000111200010200110001100001000000?001000100000000000111000000000?00100100?0000100000000020?00?00?000?00????0??0?0???000

Lesnelat

000000001000?1000001100000010??100000000???1011110210010011201001100000000001021110?10101000000000?004100000?000001020100020000?1100101001000100?00????0??0?0???0?0

Margogas

?00?0002000001001?22100000011110010000?????10112100????0?01100?0110000100??0????????????10000000?0100??????????????????????????????????????????????????????????????

MigiwaCu

00000000101001000000110000110011010100111000001111221110201200000100001000000001???110001100000000100000000110110011210001300001000010?01101?000?01000?0??0?0???0?0

Monadicu

00000100100000000022111000210011010110?01000001111201110211201002000001000100001?0?1100011001300011001000002001000102000003?000000000??00101?001101010????0?0???0?0

NegaAmer

00010000101001010001100000?02??1010000001101012103201010211210001100001001?0???????????0100000000010041001001000001020000020001000000??001000210?00????0??0?0???010

Negastri

0100010010100100001101000011001101010011100000?111200110201210000110011000100021????0000000000000110010000001?010111200001300001000010?00100?000?00????0??0?0???000

Neoarhap

00000000?01001101143100000110011010?0111000000111022111020121100100001100010001110010000010012000010000000000?11000020100030000000000??01120?000?01010?0??0?0???0?0

Neocardi

100?000010000100100?100000?12??10100000000010012012000100012000011000010001001010101000010000000001000000001101000102000002000?????????????????????????????????????

Neohypdo

000000011010010010011000001100110101000111000011112011102012100001000110001000211???10001100000000100000000010110011200001311001000010?11101?000?01000?0??0?0???0?0

Nyctor__

00000000100001000001000000?10??10000000011110012002?0000001200001000001000100101???11010100000000010041001010011001020000020000000000??100000100?00????1?00?0???1?0

Odontoca

????0?01101001001100000000111100010000000000101210100000001210000100000000100001010010101000?2011000000000010010001020000120011000100?000?000100?00???01000110010?0

Oedostet

000000001010010010001000001100110101001000000011112011102012010001000010001000211??11000010000010010000000010011001120000131100100000??11101?000?01000?0??0?0???000

ParacHum

00000100101001001001000000?11??00100000010001011110000000012000001000000001001010100101010000000101000000000?001001020000020000000000?0001001200?10???0101000???0?0

Paracard

00000000101001001000100010?12??10100001000001012100000000112100001000010001010010101101000000000100000100000101000102031002?000100100?1000000100?00???0100010???100

Paracsub

00000101201001001000000000?12??0010000100000101110000000001210000100010100100011010110100000100011000000000?1011011120000120000100000?0001001200?10???0101000???010

Paradonu

010001001010010010111000001100110101000111000011110001102012010010000010001000?1100?100000000000001001001101000101112000001?00110000???0?100???0?00????0??0?0???000

Paraplat

00000101101001001100100000?11??001000010000010111010000000121000010110000010011110011010100010011000100000021010001020000020000100000?00??000100?10???1101000???1?0

Patricie

00000001020001001011100000?10??1000000000111011211011010011210000100001100101101???1101010000000101003?00101?01100102010013001?????????????????????????????????????

Phorocar

000000001010010010?00001001111000100001000100012101000100112000001000000000011010100101010000001100000000000?000001020100020000100100?1000000100?00???11000010011?0

PhorDioc

00000002101001001000?00000112100010000110000101210100000?01211000100000000100101????10100000000111000?????????????????????????1100100?1000000100?00???1100010???1?0

PlatIndi

00000000101001001000100001111101010000100010101210100000001200000100001000101101010110100000?00100100?????????????????????????1100100?0100000100?00???11000111010?0

Pronegas

0???1100101001001011110000110011010100110000001111221110201200000100001001?0???????????00000020000100???????10??????20000132?00000000??01100?000?00????0??0?0???0?0

Quasimus

010001001010010000110000002100110101101001000011102001101012101110000010001000?1????000000000200011001000000?00001102000012000010000???????????0?01101?0??0?0???010

Rivulico

00000100100001000001011000?12??00101001000110012101010102012100121000110001000?1?10110000000000010100100010010100110200001300011000000?01110?000?00???????0?????000

Teslasen

?00?0000000001001000000000?00??00000000001111110100000100012100001001010000001110100101010001001001004000102???????????????????????????????????????????????????????

Tripchus

00010001220101001100100100?11??00100000010111010101100000012100001011000001001010100101010001002100010000002000000102000002?000001100?????00???0?10???0102000???0?0

Tripoide

00010000101001001100000100?11??00100001000000012101000000012100001001000001000110101101000000201000000000102101100102100002000?????????????????????????????????????

Tropidip

000000001000010010000000001120010100001000001012101100100012100001011000001001010100101010000010?1011010000?0100000020100020000100100?1000001100?00???0100000???1?0

Tropihyp

000001001010010010011011001020110101001111000000110010101012010001100010000000211000100000000000111001000002000100100?0001111000001010????00?000?00????0??0?0???000

YukoanaC

000001021010010010001000002100110101101101000011000001102012110110000010001000210?0?000001000200011001000100?00001102000002?000100??0??11101?000?00??1?0??0?0???0?0

Zorochro

00001100101001001011110000110011010110111000001111201110201201000100011000100021???1000000001000101001000000?001011020000030001101000??01101?000?00????0??0?0???000
